# Electron-injection-induced global aromaticity enables stable open-shell nanopillars with intense mid-infrared magnetic circular dichroism

**DOI:** 10.1039/d6sc01782g

**Published:** 2026-06-01

**Authors:** Kongchuan Wu, Shicheng Dong, Qiqi Chen, Yuanfeng Pan, Jun Zhu, Jianbin Lin, Hui-Jun Zhang

**Affiliations:** a Department of Chemistry, Fujian Key Laboratory of Chemical Biology, College of Chemistry and Chemical Engineering, Xiamen University Xiamen 361005 Fujian China meghjzhang@xmu.edu.cn; b Guangxi Key Laboratory of Petrochemical Resource Processing and Process Intensification Technology, School of Chemistry and Chemical Engineering, Guangxi University Nanning Guangxi 530004 China; c Guangdong Basic Research Center of Excellence for Aggregate Science, School of Science and Engineering, The Chinese University of Hong Kong (Shenzhen) Longgang Shenzhen Guangdong 518172 China jun.zhu@cuhk.edu.cn

## Abstract

Open-shell species are central to spintronics and infrared optoelectronics, but remain challenging to stabilize in discrete molecular systems. Herein, we report that electron injection into pillar-shaped, radially π-conjugated [4]cyclonaphthodithiophene diimides ([4]C-NDTIs) triggers global aromaticity, yielding radical species with notable stability and optical properties. These globally aromatic radicals exhibit record-high mid-infrared (MIR) absorption (*ε* up to 10^5^ M^−1^ cm^−1^), strong near-infrared (NIR) chiroptical activity (*g*_CD_ up to 2.4 × 10^−2^), and exhibit MIR magnetic circular dichroism (MCD) with gMCD up to 5.0 × 10^−3^ T^−1^. In solution, the radical monoanion exhibits superior kinetic stability (*τ*_1/2_ = 4.3 days) compared to the diradical dianion (*τ*_1/2_ = 1.5 days), revealing a balance between aromatic stabilization and charge destabilization. These properties persist even upon C_60_ encapsulation, underscoring the robustness of the aromaticity-based design. This work establishes electron-injection-induced global aromaticity as a general strategy for stabilizing open-shell species while unlocking long-wavelength chiroptical and magneto-optical functionalities.

## Abstract 2

Electron injection into [4]C-NDTI nanopillars generates a globally aromatic open-shell system that exhibits intense MIR absorption and strong chiroptical activity in the NIR region , which open new avenues for infrared optoelectronics.

## Graphical abstract

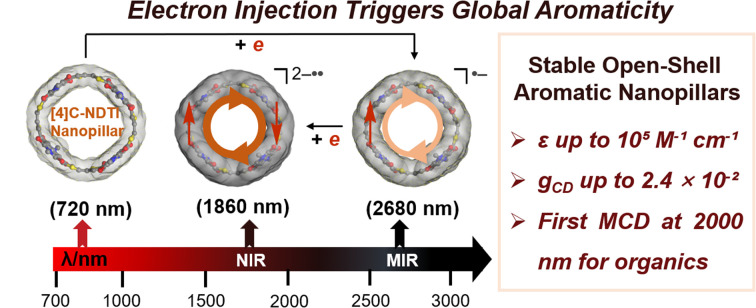

## Introduction

The mid-infrared (MIR) spectral region (*ca.* 2–5 µm) is a technological frontier for sensing, thermal imaging, and secure communications.^1–3^ The development of these technologies demands molecular systems that exhibit intense molecular absorption,^4^ high environmental stability, and even chirality to provide additional functions like circular dichroism (CD) detection and spin-polarized charge transport.^5,6^ While π-conjugated organic molecules are promising candidates due to their structural tunability and solution processability, their strong electronic absorptions (*ε* ≈ 10^3^–10^5^ M^−1^ cm^−1^) are typically confined to the visible and near-IR (NIR) regions, originating from intense π–π* transitions.^7^ By contrast, MIR absorption in organic systems largely relies on weak vibrational modes (*ε* < 100 M^−1^ cm^−1^), a fundamental limitation that has precluded the development of efficient organic MIR chromophores. Moreover, the intrinsically weak chiroptical response^8^ and minimal Zeeman splitting^9^ in this low-energy window have rendered techniques like CD and magnetic circular dichroism (MCD)^10^ nearly inaccessible for classical organic conjugated frameworks, thereby creating a fundamental scientific challenge for advanced infrared photonics and spintronics.

A promising route to enhance low-energy absorption involves populating polaronic or bipolaronic states *via* chemical or electrochemical reduction/oxidation of π-conjugated backbones.^11^ This approach suppresses dominant π–π* transitions and introduces lower-energy electronic absorptions. However, extending this absorption into the MIR region while retaining stability remains a formidable challenge.^12^ Moreover, imparting strong and stable chiroptical activity to such open-shell intermediates is exceptionally rare, as most radical ions suffer from rapid degradation^13^ and diminished anisotropy factors at longer wavelengths.

Fully conjugated π-macrocycles,^14–16^ especially those with radial conjugation,^17,18^ offer a compelling structural platform to address these challenges. Unlike their planar counterparts, which often struggle to balance electronic tunability with kinetic stability, radially conjugated macrocycles employ geometric strain to create a highly responsive electronic environment. Their cyclic architectures promote charge delocalization, enhance thermodynamic stability, and can exhibit global aromaticity.^19–22^ This property is known to dramatically influence the electronic structure and optical properties.^23–26^ Yet, most known macrocycles lack stable open-shell states, built-in chirality tunability, and the long-sought ability to undergo predictable aromaticity switching upon electron injection,^27–29^ which are key features needed to achieve strong and tunable MIR chiroptical responses.

**Fig. 1:**
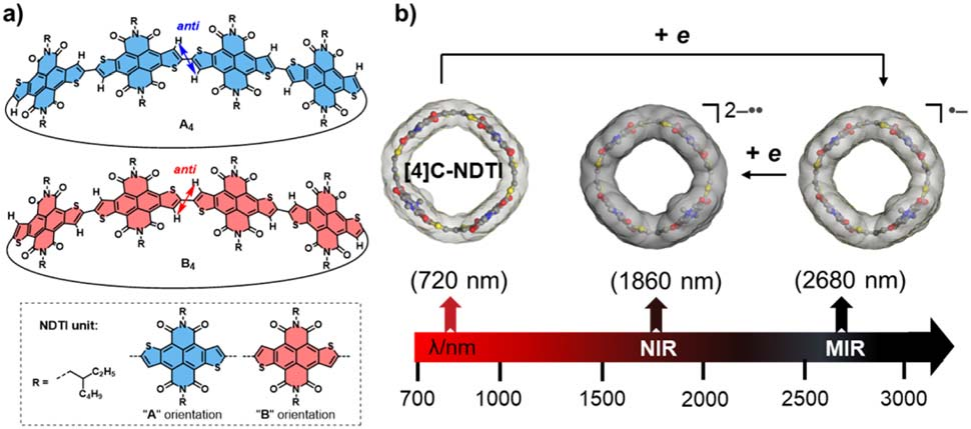
(a) Chemical structures of the enantiomeric pair A_4_ and B_4_ of [4]C-NDTI. A and B denote the orientation of each NDTI unit; the inter-unit torsional angles are very small (2.8–4.4°). (b) Featured optical response of neutral, radical anion, and diradical dianion states of [4]C-NDTI (top-view of the A_4_ crystal structure; simplified as a circle).

We recently reported the synthesis of one type of pillar-shaped, radially π-conjugated macrocycle [4]cyclo-naphthodithiophene diimide ([4]C-NDTIs, Fig. 1a, representative enantiomers A_4_ and B_4_).^30,31^ Comprising electron-deficient NDTI^32^ units with thiophene–thiophene linkage, these architectures exhibit extended conjugation, well-defined pillar topology, and positive electrostatic potentials on the convex surface.^33,34^ Herein, we go beyond synthesis and demonstrate that stepwise chemical reduction of enantiopure A_4_/B_4_ generates stable radical anions (A_4_˙^−^/B_4_˙^−^) and diradical dianions (A_4_^2−^˙˙/B_4_^2−^˙˙) that exhibit high molar extinction coefficients (*ε* up to 10^5^ M^−1^ cm^−1^) extending deep into the MIR region (>2500 nm, Fig. 1b). Importantly, these charged species retain the inherent chirality of the parent macrocycle, enabling observation of intense, mirror-image CD signals across the NIR-MIR window. Density functional theory (DFT) calculations reveal that electron injection triggers a topological aromatic transition, transforming a locally conjugated neutral ring into globally aromatic open-shell species. This electronically driven aromaticity switch not only explains the extraordinary optical stability but also facilitates the unprecedented detection of MCD responses in the MIR regime for an organic molecule. Finally, we show that this aromaticity-based optical regulation is robust enough to persist even when an electron acceptor (C_60_) is encapsulated within the macrocyclic cavity, underscoring the dominance of the host's frontier orbitals.

## Results and discussion

**Fig. 2:**
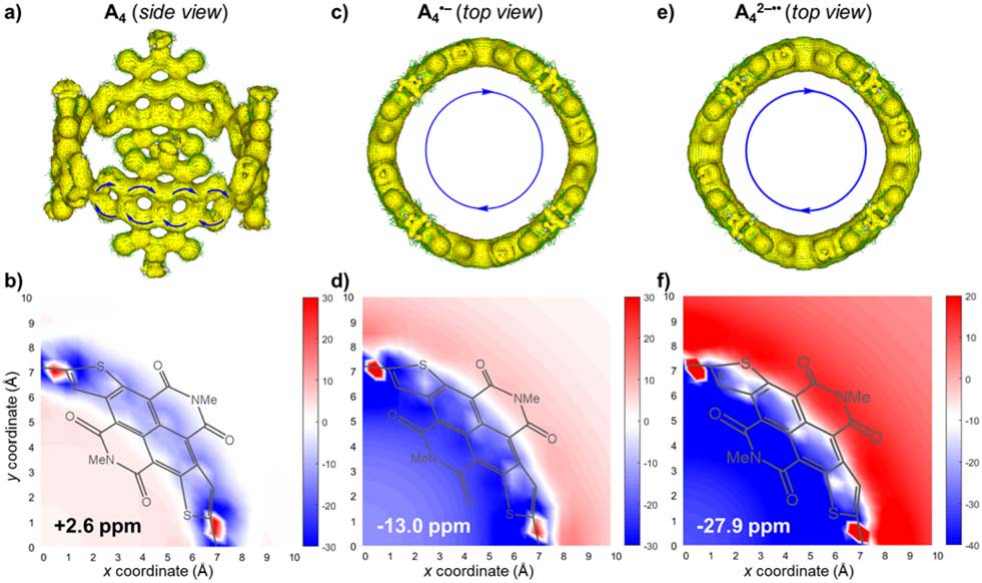
(a, c and e) ACID plots (isovalue: 0.032; arrows indicate the direction of the ring current) and (b, d and f) NICS-grid contour plots (values in ppm) in the cross-sectional *x*–*y* plane of the nanopillar molecule for A_4_, A_4_˙^−^, and A_4_^2−^˙˙. The color scale represents the computed magnetic shielding strength along the cylindrical axis (*z*-direction). Key NICS(1)_*zz*_ values are indicated on the plots. Note: The 2-ethylhexyl substituent on [4]C-NDTI was modeled as a methyl group to streamline the calculations.

We initiated our studies by evaluating the electronic structures of all six topological isomers of [4]C-NDTIs (A_4_, B_4_, A_2_B_2_, ABAB, A_3_B, and AB_3_) using DFT calculations (Table S1 and Fig. S1–S5). Taking A_4_ as a representative, we first analysed its π-electron delocalization pattern (Fig. 2). Anisotropic current-induced density (ACID) plots reveal that ring currents are strongly localized within each individual NDTI core, with markedly weaker electronic communication across the thiophene–thiophene linkers (Fig. 2a and S15). This indicates a predominantly localized π-delocalization pattern rather than a fully global cyclic circuit. Consistently, nucleus-independent chemical shift (NICS) calculations in the cross-sectional *x*–*y* plane (the plane perpendicular to and bisecting the nanopillar's principal axis) show pronounced negative (shielding, Fig. S12) regions directly over each NDTI unit and only slightly positive values (NICS(1)_*ZZ*_: +2.6 ppm) near the central cavity (Fig. 2b, S3 and Table S2). Quantitative electron density of delocalized bonds (EDDB) analysis (Fig. S4) further confirms that the delocalized π-electron density is largely confined to individual NDTI units, with minimal inter-unit contribution.

Complementary DFT calculations elucidate how electron injection reshapes the nanopillar's aromaticity. Upon one-electron reduction (A_4_˙^−^), the NICS distribution in the cross-sectional plane shows enhanced shielding in the central region (Fig. 2b and Table S4, NICS(1)_*ZZ*_: −13.0 ppm), reflecting the emergence of a global ring current associated with the delocalized spin. The emergence of a global ring current in A_4_˙^−^ addresses a fundamental question in open-shell aromaticity. Unlike closed-shell Hückel aromatics (4*n* + 2) or triplet-state Baird aromatics (4*n*),^16,35^ odd-electron radicals lack a unified aromaticity rule.^36^ Recent studies suggest that radical ions can exhibit higher geometric aromaticity (HOMA index) than their neutral parents,^37^ a counterintuitive notion that aligns with our observation. Upon two-electron reduction, the transformation becomes even more pronounced. The NICS contour plot of A_4_^2−^˙˙ displays a strong, contiguous shielding area spanning the entire interior of the nanopillar, accompanied by deshielding regions outside the ring (Fig. 2c and Table S7). This “inside-shielding and outside-deshielding” pattern is characteristic of a global aromatic ring current (NICS(1)_*ZZ*_: −27.9 ppm), arising directly from the coherently delocalized π-electron system of the diradical dianion. Energy comparisons confirm that the resulting species (A_4_^2−^˙˙) adopts an open-shell singlet ground state with significant diradical character (Table S5). Furthermore, the calculated diradical index (*y*_0_) exceeding 0.6 provides additional evidence for this feature (Table S6). Geometric bond length parameters, spin population analysis, and the fully delocalized nature of frontier molecular orbitals (Fig. S8, S10 and S11) reveal completely delocalized electronic structures for both A_4_˙^−^ and A_4_^2−^˙˙. Furthermore, systematic DFT calculations on the complete set of topological isomers confirm that this electron-injection-induced transition is a general feature of the [4]C-NDTI architecture (Fig. S6 and S7). In all cases, both one- and two-electron reduction lead to the formation of open-shell aromatic species, whose NICS profiles consistently exhibit the characteristic shielding pattern of a global ring current. This aromaticity switching from closed-shell local to open-shell global is reminiscent of the Hückel–Baird reversal in excited states but occurs here in ground-state radicals through redox control.

**Fig. 3:**
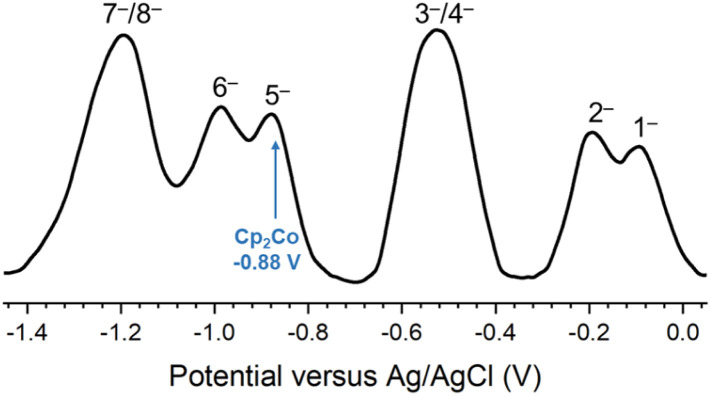
Square wave voltammogram of B_4_ in CH_2_Cl_2_ in the presence of NBu_4_PF_6_ (0.1 M) with Ag/AgCl as the reference electrode. The arrow indicates the reduction potential of Cp_2_Co. At least six reduction events are resolved, and the overall pattern is consistent with the macrocycle accepting up to eight electrons (the 3rd/4th and 7th/8th couples are unresolved).

With B_4_ in hand, we first probed its redox properties by square-wave voltammetry (SWV). The SW voltammogram (Fig. 3) reveals reduction features consistent with up to eight sequential one-electron reductions in the range of 0.0 to −1.4 V (*vs.* Ag/AgCl), confirming the multi-electron-accepting ability of the nanopillar. The first reduction occurs at −0.09 V, which is markedly less negative than that of the NDTI monomer (*E*_red_^1^ = −0.49 V and *E*_red_^2^ = −0.89; Fig. S24).^38^ This enhanced electron affinity may result from the extended conjugation and electronic coupling in the nanopillar architecture.

**Fig. 4:**
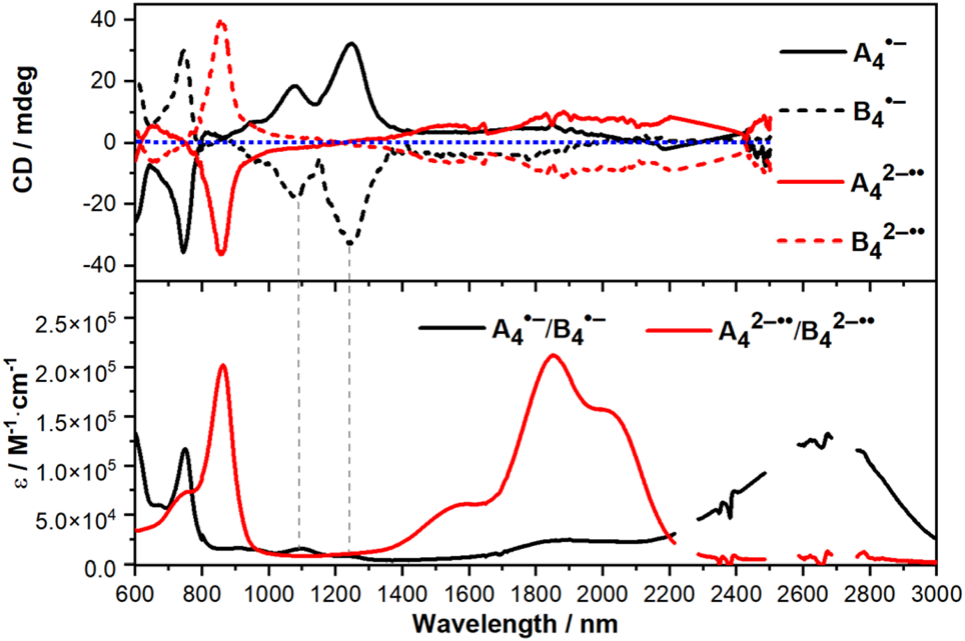
Electronic absorption and CD spectra of A_4_˙^−^/B_4_˙^−^ (black) and A_4_^2−^˙˙/B_4_^2−^˙˙ (red) in CH_2_Cl_2_ (5.0 × 10^−6^ M). For the absorption spectra, solvent absorption peaks (∼2200, 2500, and 2700 nm) are omitted for clarity. Measurements of CD could not be extended to the strongest absorption maximum at ∼2650 nm due to the instrumental cut-off at 2500 nm.

We then monitored the chemical reduction of both enantiomers, A_4_ and B_4_, by UV-vis-NIR-MIR spectroscopy using cobaltocene (CoCp_2_, *E*^0^ = −0.88 V *vs.* Ag/AgCl) as the reductant. Addition of 1 equiv. of CoCp_2_ to either enantiomer resulted in the decay of the neutral-state absorptions (530 and 598 nm) and the emergence of a sharp peak at 760 nm together with a broad MIR band extending to 3000 nm (*λ*_max_ = 2680 nm, Fig. 4). These spectral features are diagnostic of the radical anions (A_4_˙^−^/B_4_˙^−^). Further addition of CoCp_2_ (>1 equiv.) caused a blue shift of these bands and gave rise to new absorptions at 1860 nm (strong) and 2010 nm (shoulder), consistent with the formation of the open-shell diradical dianions (A_4_^2−^˙˙/B_4_^2−^˙˙). Notably, no further spectral evolution was observed even with a large excess of CoCp_2_ (up to 4 equiv.), indicating a substantial kinetic or thermodynamic barrier to reduction beyond the two-electron stage. For comparison, reduction of the NDTI monomer with 1 equiv. CoCp_2_ produced only the radical anion NDTI˙^−^ (Fig. S27), characterized by structured absorptions between 600 and 920 nm, with no further spectral evolution upon addition of more reductant.

More interestingly, the intense long-wavelength absorption bands described above are accompanied by equally prominent and mirror-symmetric circular dichroism (CD) signals (Fig. 4). Such strong CD activity extending into the NIR and even MIR region is exceptionally rare for organic molecules, as anisotropy factors typically diminish at lower energies. The radical anions A_4_˙^−^ and B_4_˙^−^ display pronounced mirror-image CD spectra featuring three intense bands between 700–1300 nm. For the diradical dianions A_4_^2−^˙˙ and B_4_^2−^˙˙, an exceptionally broad, mirror-symmetric CD signal spans 1250–2500 nm, which represents a rare observation for purely organic systems in this spectral region. All the reduced species exhibit high dissymmetry factors (*g*_CD_) above a wavelength of 700 nm (Fig. S38), reaching 2.3 × 10^−2^ at 1230 nm for the radical anions (A_4_˙^−^/B_4_˙^−^) and 2.4 × 10^−2^ at 857 nm for the diradical dianions (A_4_^2−^˙˙/B_4_^2−^˙˙). These values rank among the highest reported for organic π-conjugated systems in the NIR region,^6,39^ highlighting their potential for advanced chiroptical applications.

**Fig. 5:**
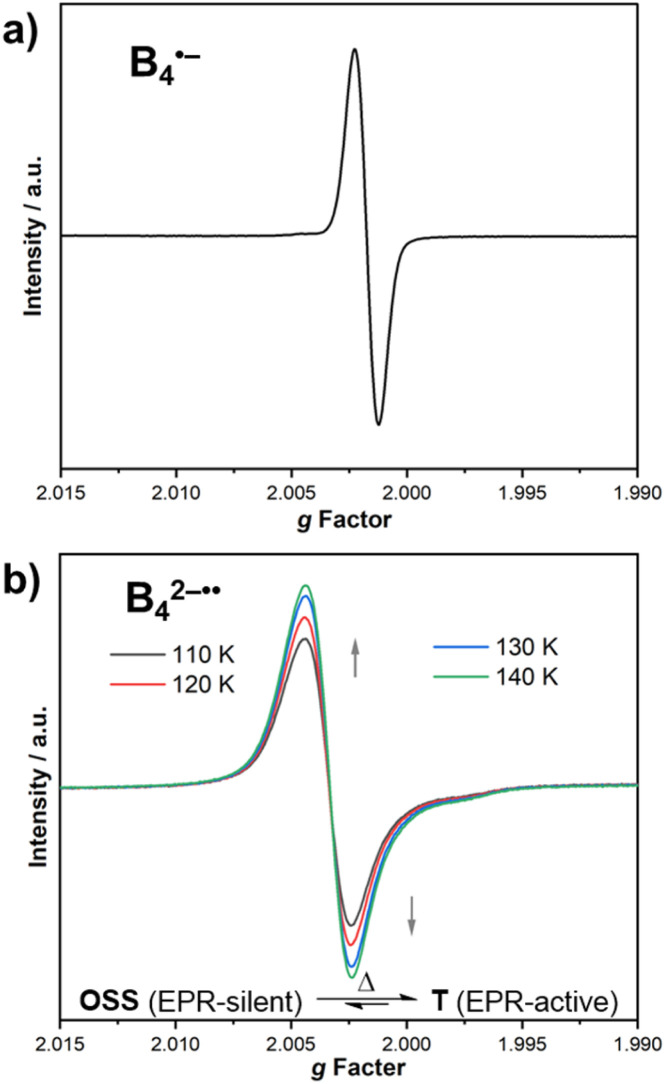
(a) EPR spectrum of B_4_˙^−^ at 110 K in CH_2_Cl_2_; (b) variable-temperature EPR spectra of B_4_^2−^˙˙ in CH_2_Cl_2_, showing thermal population of the triplet state (OSS: open-shell singlet state; T: triplet state).

Electron paramagnetic resonance (EPR) spectroscopy provided direct evidence for the spin delocalization of the reduced species. The radical anion B_4_˙^−^ exhibited a narrow, isotropic single-line signal with a *g*-factor of 2.0017 (Fig. 5a). This value approaches that of a free electron and contrasts with the broader signal typically observed for localized organic radical anions (*e.g.*, the monomeric NDTI˙^−^, *g* = 2.0032, Fig. S32), indicating highly delocalized spin density over the entire π-conjugated nanopillar architecture. For the diradical dianion B_4_^2−^˙˙, DFT calculations establish the energetic landscape of its spin states (Fig. S10 and Table S5). The closed-shell singlet is calculated to be the highest in energy, with the triplet state intermediate and the open-shell singlet serving as the ground state, confirming significant diradical character. The open-shell singlet lies only slightly below the triplet state (Δ*E*_T–S_ ≈ 1.7 kcal mol^−1^). Although a pure open-shell singlet diradical is normally EPR-silent due to spin-pairing, the small Δ*E*_T–S_ allows thermal population of the paramagnetic triplet state even at low temperature, thereby enabling EPR detection (Fig. 5b). A marked increase in signal intensity upon warming from 110 to 140 K is consistent with thermal population of the triplet state. Moreover, the measured spectrum exhibits a substantially broadened signal (*g* = 2.0033), which directly reflects the strong spin–spin coupling and exchange interaction between the two delocalized unpaired electrons characteristic of this diradical system.

**Fig. 6:**
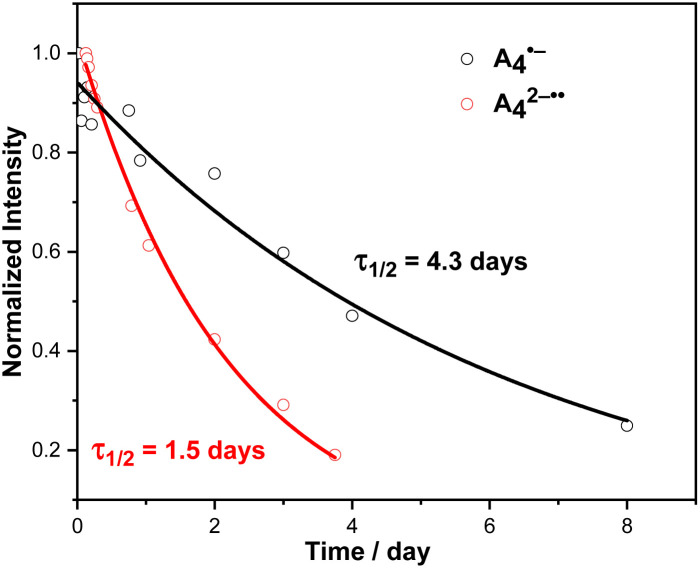
Plot of optical density decay of A_4_˙^−^ (at 2580 nm) and A_4_^2−^˙˙ (at 1860 nm) in deaerated CH_2_Cl_2_ (5.0 × 10^−6^ M) at room temperature.

To evaluate the kinetic stability of the generated radical species, the time-dependent decay of the NIR absorption maxima (2580 nm for A_4_˙^−^ and 1860 nm for A_4_^2−^˙˙) was monitored. The absorbance at these wavelengths exhibited a clear exponential decay over time, regenerating neutral/monoanion species, which was accurately fitted to a first-order kinetic model (Fig. 6 and S29–S31). The resulting half-lives (*τ*_1/2_) were determined to be 4.3 days and 1.5 days, respectively. The stability ordering reflects a balance between two opposing effects of electron injection: while two-electron reduction enhances global aromaticity, it also increases electron density, heightening susceptibility to quenching. The accelerated decay observed for the dianion is therefore attributable to its higher charge density, which increases its susceptibility to quenching by residual protic impurities or trace oxygen. Notably, these half-lives are remarkably longer than those of common naphthalene diimide-based radical anions, which are typically minutes to hours under similar conditions.^40^ This notable kinetic persistence is primarily ascribed to the global aromaticity of the system,^41,42^ which effectively delocalizes both spin and charge density, providing a robust electronic barrier against common degradation pathways.

**Fig. 7:**
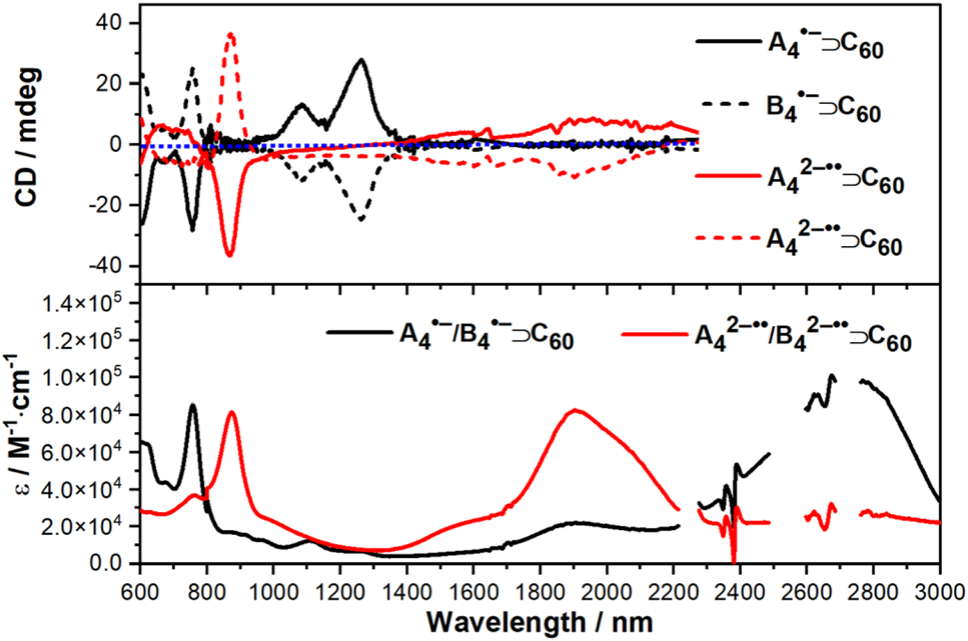
Electronic absorption and circular dichroism (CD) spectra of A_4_˙^−^⊃C_60_/B_4_˙^−^⊃C_60_ (black) and A_4_^2−^˙˙⊃C_60_/B_4_^2−^˙˙⊃C_60_ (red). In the absorption spectra, solvent absorption peaks (∼2200, 2500, and 2700 nm) are omitted for clarity. The CD spectra are displayed over a shorter wavelength range (to 2300 nm) than the absorption spectra.

The well-defined cavity of the [4]C-NDTI nanopillar strongly binds fullerenes,^30,31^ forming a stable 1 : 1 complex [4]C-NDTI⊃C_60_ with a binding constant of ∼1.9 × 10^8^ M^−1^. To examine whether the electron-injection-induced aromaticity of the host is perturbed by a potent, encapsulated electron acceptor, we studied the chemical reduction of A_4_/B_4_⊃C_60_. Stepwise addition of CoCp_2_ to the complex afforded absorption spectra identical to those of free A_4_ and B_4_ (Fig. 7 and S26). Specifically, 1 equiv. of reductant generated a characteristic sharp band at 760 nm and the broad MIR absorption of A_4_˙^−^ and B_4_˙^−^, while 2 equiv. produced the diagnostic features of A_4_^2−^˙˙ and B_4_^2−^˙˙ at 1860 and 2010 nm. Notably, no spectral signatures of the fullerene radical anion C_60_˙^−^ (*e.g.*, bands near 934 and 1078 nm) were observed.^43^ The assignment is further corroborated by EPR spectroscopy (Fig. S33), which shows that the signals of B_4_˙^−^⊃C_60_ and B_4_^2−^˙˙⊃C_60_ closely resemble those of the pristine reduced nanopillars. These results provide clear evidence that the added electrons are exclusively localized on the radially conjugated π-system of the host, demonstrating that the global aromatic transition is governed by the frontier orbitals of the [4]C-NDTI scaffold itself, independent of a highly reducible guest. This exclusive localization of electrons on the host scaffold, which aligns perfectly with recent theoretical predictions,^44^ experimentally validates that the global aromatic transition is an intrinsic property of the [4]C-NDTI framework. On the other hand, despite C_60_'s high electron affinity, its electronic structure remains shielded within the complex, revealing a strategy to prevent C_60_ reduction.^45^

**Fig. 8:**
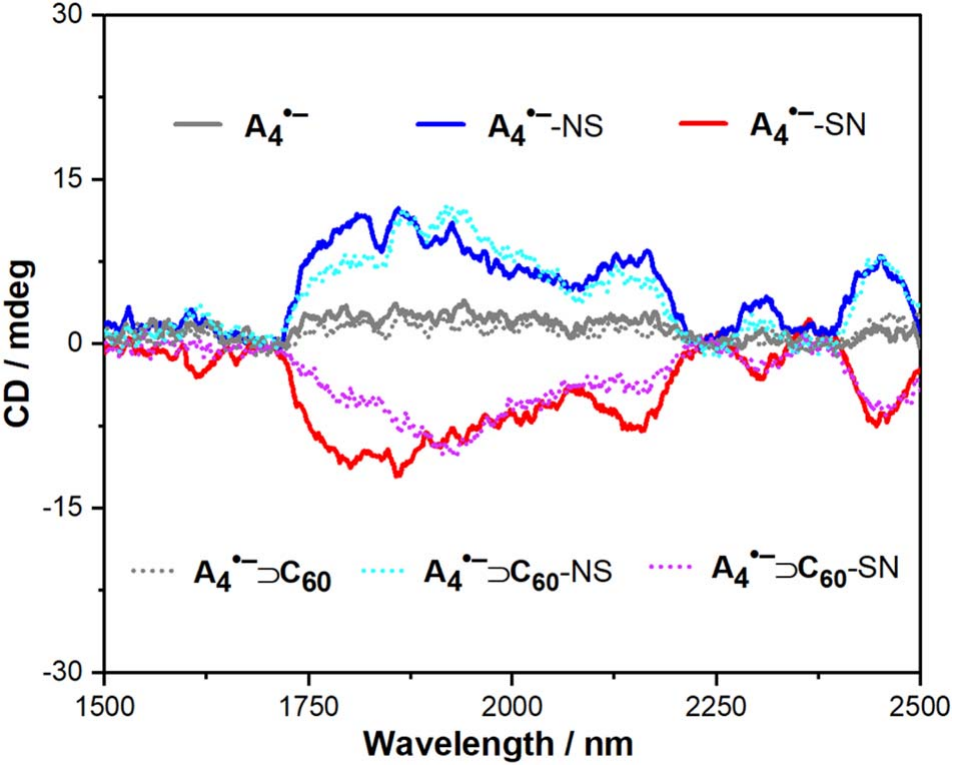
CD spectra of A_4_˙^−^ and A_4_˙^−^⊃C_60_ in CH_2_Cl_2_ at room temperature under 1.6 T in the NIR and MIR band.

The robust radical-based states prompted us to explore their rarely accessed magneto-optical response. The magneto-optical properties of A_4_˙^−^ and its host–guest complex A_4_˙^−^⊃C_60_ were investigated under an external magnetic field (1.6 T). As illustrated in Fig. 8, altering the magnetic field direction (NS-SN) induced a pronounced and reproducible sign inversion of the CD signal in the 1600–2500 nm range, directly demonstrating the magneto-optical response. The magneto-circular dichroism (MCD) spectra (Fig. S42), derived as [CD(NS)-CD(SN)]/2,^9,10,46^ exhibit unidirectional Gaussian-shaped bands characteristic of faraday terms arising from either field-induced state mixing 
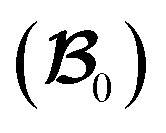
 or the spin-degenerate ground state 
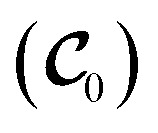
 of the radical anion.^47^ This behavior is consistent with Michl's perimeter model, which describes the electronic structure of aromatic π-systems as derived from a higher-symmetry parent perimeter.^48^ Although the absorption maximum for A_4_˙^−^ lies at approximately 2680 nm, CD measurements under field could not be extended beyond 2500 nm due to instrumental cut-off. The MCD spectrum of B_4_˙^−^ was also measured (Fig. S41 and 42) and found to be essentially identical to that of A_4_˙^−^, confirming that the MCD is independent of molecular chirality. To our knowledge, this constitutes the first observation of MCD at around 2000 nm in a discrete organic molecular system.^49,50^ The dissymmetry factors *g*_MCD_ reach 4.6 × 10^−3^ T^−1^ for A_4_˙^−^ and 5.0 × 10^−3^ T^−1^ for A_4_˙^−^⊃C_60_ at around 1900 nm (Fig. S43). The magnitude of these *g*-factors signifies a substantial magneto-optical response in the infrared regime, which is notable for organic radicals that typically exhibit weak spin–orbit coupling. This preliminary discovery decisively showcases that stable organic radical anions can serve as a promising platform for achieving long-wavelength magneto-optics,^51,52^ highlighting the unique potential of these nanopillar-based radical systems.

## Conclusion

In conclusion, we have established electron-injection-induced global aromaticity as a design principle for stabilizing open-shell species while unlocking long-wavelength optical functionalities. This concept is realized in pillar-shaped, radially π-conjugated [4]C-NDTIs. Both the radical monoanions ([4]C-NDTI˙^−^) and diradical dianions ([4]C-NDTI^2−^˙˙) exhibit exceptionally intense and broadly tunable absorption, achieving record-high molar extinction coefficients in the MIR region. The topological aromatic transition triggered by electron injection generates globally aromatic species with exceptional kinetic stability. The combination of intense long-wavelength absorption, inherent chirality, and open-shell character enables both CD in the NIR/MIR region and the measurements of MCD (*g*_MCD_ up to 5.0 × 10^−3^ T^−1^) in the MIR region. The persistence of these properties upon C_60_ encapsulation underscores the robustness of this design. This work expands the conceptual framework of aromaticity into the open-shell regime and establishes a molecular platform for advanced infrared optoelectronics, chiral sensing, and magneto-optical devices.

## Author contributions

K. W. and Y. P. synthesized and characterized [4]C-NDTIs and the corresponding radical species. S. D. performed all DFT calculations. K. W. conducted UV-vis-NIR absorption, CD, EPR, and MCD spectroscopy. Q. C. repeated the MCD experiments and analysed the data. H.-J. Z. and J. L. conceived the project. H.-J. Z. and J. Z. supervised the work.

## Conflicts of interest

There are no conflicts to declare.

## Supplementary Material

SC-OLF-D6SC01782G-s001

## Data Availability

The authors confirm that the data supporting the findings of this study are available within the article and its supplementary information (SI). Supplementary information: experimental procedures, synthesis and characterization, computational details, and additional spectroscopic data (UV-vis-NIR, CD, EPR, MCD, kinetic stability, control experiments). See DOI: https://doi.org/10.1039/d6sc01782g.
