# Supplementary material for: Electron-injection-induced global aromaticity enables stable open-shell nanopillars with intense mid-infrared magnetic circular dichroism
Source: Chem Sci. 2026 Jun 1. Online ahead of print. doi: 10.1039/d6sc01782g (PMC13286027; doi:10.1039/d6sc01782g)
Supplement: SC-OLF-D6SC01782G-s001 [file SC-OLF-D6SC01782G-s001.pdf]

Supplementary Information (SI) for Chemical Science.
This journal is © The Royal Society of Chemistry 2026

This version of the Supporting Information, published 18/06/26, replaces the version published previously on 01/06/26 to include additional data (<S²> values, wavefunction stability, diradical index, α/β orbital labels, and TD-DFT explanation).

Supporting Information for

Electron-Injection-Induced Global Aromaticity Enables Stable Open-Shell Nanopillars with Intense Mid-Infrared Magnetic Circular Dichroism

Kongchuan Wu‡, Shicheng Dong‡, Qiqi Chen, Yuanfeng Pan, Jun Zhu,* Jianbin Lin, and Hui-Jun Zhang*

I. General Methods and Materials 2

II. Computational Details and Supplementary DFT Results 3

III. Electrochemical Properties 29

IV. UV-Vis-NIR Absorption Spectra: Chemical and Electrochemical Reduction 30

V. Electron Paramagnetic Resonance (EPR) Spectroscopy: Spin Delocalization and Diradical Character 34

VI. Chiroptical Properties: CD and MCD Spectra 36

VII. Cartesian Coordinates 40

VIII. Reference 103

I. General Methods and Materials

General methods

Unless otherwise specified, reactions were performed using glovebox techniques under an argon atmosphere. Anhydrous dichloromethane (DCM) was obtained by distillation over calcium hydride. All other reagents were purchased from commercial suppliers and used as received. Mass spectrometry analyses were performed using a MALDI-TOF mass spectrometer and *trans*-2-[3-(4-*tert*-butylphenyl)-2-methyl-2-propenylidene]malononitrile (DCTB) as the matrix. UV-vis-NIR absorption spectra were recorded on an Agilent Cary 5000 spectrophotometer with superb photometric performance in the 175-3300 nm range. Cyclic voltammetry (CV) measurements were performed on a Bio-Logic SP-200 potentiostat (EC-LAB software). Circular dichroism (CD) spectra was acquired on a JASCO J-1700 spectropolarimeter (a full-spectrum scan from 163 to 2500 nm in a single experiment). Magnetic circular dichroism (MCD) spectra were recorded on the same JASCO J-1700 instrument equipped with a 1.6 T permanent magnet. Electron paramagnetic resonance (EPR) spectra were measured on a Bruker EMXplus-9.5/12 spectrometer. For UV-vis-NIR, CD, and MCD measurements, samples were prepared in DCM at a concentration of 5.0×10^{-6} M. Absorption and CD spectra were recorded at room temperature using a 10 mm path length quartz cuvette and placed in a 10 mm colorimetric dish for room temperature spectrum testing. For MCD measurements, a 5 mm path length quartz cuvette was used. And MCD is measured on the same spectrometer while a 1.6 T magnet was put into the sample cell under two conditions: (1) the magnetic direction was parallel with the incident light (N−S, +1.6 T); (2) the magnetic direction was antiparallel with the incident light (S−N, -1.6 T). The *g*-factor of MCD (g_{MCD}) is calculated according to the formula: g_{MCD} = MCD/(32980×A×B), where A is the value in the absorption spectrum related to measuring the MCD spectrum, and B is the strength of the magnetic field. The difference between the *g*-factor of CD and MCD is that the *g*-factor of MCD requires normalization of the magnetic field. **[4]C-NDTI** and the radical anions were synthesized as described in our previous paper.[1]

Sample preparation

[4]C-NDTI$^{\bullet-}$ solution: In a glovebox, a stock solution of **[4]C-NDTI** (5.0×10^{-6} M in DCM) was prepared by dissolving 0.036 mg of the compound in 3 mL of anhydrous DCM in a sample vial. Separately, a stock solution of $CoCp_2$ (2.5×10^{-3} M in DCM) was prepared by dissolving 2.84 mg of $CoCp_2$ in 6 mL of anhydrous DCM in another vial. To generate the radical anion, 3 mL of the **[4]C-NDTI** stock solution was treated with 6 μL of the $CoCp_2$ stock solution. The mixture was shaken gently and allowed to stand for 2 min before measurement to afford the **[4]C-NDTI$^{\bullet-}$** solution.

[4]C-NDTI$^{2-\bullet\bullet}$ solution: The dianion radical species was generated by treating 3 mL of the **[4]C-NDTI** stock solution (5.0×10^{-6} M in DCM) with 12 μL of the $CoCp_2$ stock solution (2.5×10^{-3} M). The mixture was shaken gently and allowed to stand for 2 minutes to obtain the **[4]C-NDTI$^{2-\bullet\bullet}$** solution.

[4]C-NDTI⊃C_{60} solution: In a 5 mL sample vial, 0.072 mg of **[4]C-NDTI** (3.0×10^{-5} mmol)

and 0.022 mg (3×10^{-5} mmol) of C_{60} were combined and dissolved 0.5 mL of toluene, yielding an indigo-blue solution. The solvent was then removed under vacuum. The solid residue was transferred into a glovebox, redissolved in 6 mL of anhydrous DCM, and stirred until homogeneous to afford the final **[4]C-NDTI⊃C$_{60}$** solution (5×10^{-6} M). MALDI-TOF MS calcd. For $C_{196}H_{145}N_8O_{16}S_8^+$, $[M+H]^+$: 3123.86, found: 3123.33.

[4]C-NDTI$^{\bullet-}$⊃C$_{60}$ solution: In a glove box, 3 mL of the **[4]C-NDTI⊃C$_{60}$** stock solution (5.0×10^{-6} M in DCM) was treated with 6 μL of the $CoCp_2$ stock solution (2.5×10^{-3} M in DCM). The mixture was shaken gently and allowed to stand for 2 minutes, yielding the reduced **[4]C-NDTI$^{\bullet-}$⊃C$_{60}$** solution.

[4]C-NDTI$^{2-\bullet\bullet}$⊃C$_{60}$ solution: 3 mL of the **[4]C-NDTI⊃C$_{60}$** solution (5.0×10^{-6} M in DCM) was treated with 12 μL of the $CoCp_2$ stock solution (2.5×10^{-3} M in DCM). After shaking and standing for 2 minutes, the **[4]C-NDTI$^{2-\bullet\bullet}$⊃C$_{60}$** solution was obtained.

II. Computational Details and Supplementary DFT Results

Computational details

All the calculations were performed using the Gaussian 16 software package.[2] Geometry optimization of the compounds was conducted at the (U)M06-2X[3](D3)[4] density functional theory level using the basis set 6-31+g(d)[5] for all atoms. In addition, Hessian calculations for obtaining the vibrational frequencies were performed at the same level of theory as that for the geometry optimization to check whether the optimized geometrical structure is an energy minimum (with no imaginary frequency). The stability of the wavefunction was checked for all optimized structures using the "stable=opt" keyword. No occurrence of the word "instability" was found in the output file (except for the closed-shell singlet states of dianionic systems). Instead, the following standard messages were displayed: "*The wavefunction is stable under the perturbations considered. The wavefunction is already stable.*" Density matrices of natural atomic orbitals (NAO) were obtained using the NBO 7.0 program.[6] Aromaticity evaluation was carried out by nucleus-independent chemical shifts (NICS, including NICS-grid and NICS-scan plots)[7] at (U)CAM-B3LYP[8]/6-31+g(d) level, anisotropy of the current-induced density (ACID)[9] and electron density of delocalized bonds (EDDB, using the RunEDDB script program)[10] calculations at (U)CAM-B3LYP/6-311++g(d,p) level. In addition, optimized structures were visualized by the *CYLview* program.[11] Spin densities were drawn by combining *Multiwfn 3.8*[12] and *VMD*[13]. For TD-DFT, M06-2X is used for UV/vis absorption simulation calculation with dichloromethane solvent, and the UV/vis spectra was drawn in the *Multiwfn 3.8* program.

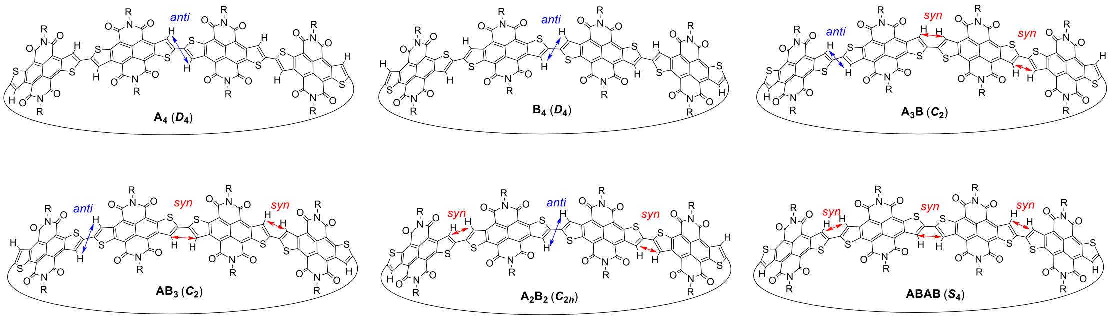

Figure S1. Structural depiction of six isomers (**A_4**, **A_2B_2**, **A_3B**, **AB_3**, **ABAB**, **B_4**) of **[4]C-NDTI**.

Table S1. Relative energies (kcal·mol^{-1}) of **A_4**, **A_2B_2**, **A_3B**, **AB_3**, **ABAB**, and **B_4**. The basis set: M06-2X-(D3)/6-31+G(d). (The 2-ethylhexyl substituent on **[4]C-NDTI** was modeled as a methyl group to streamline the calculations.)

Compound Name	**A_4**	**A_2B_2**	**A_3B**	**AB_3**	**ABAB**	**B_4**
Relative Energy	0.0	+4.5	+4.3	+4.3	+8.1	0.0

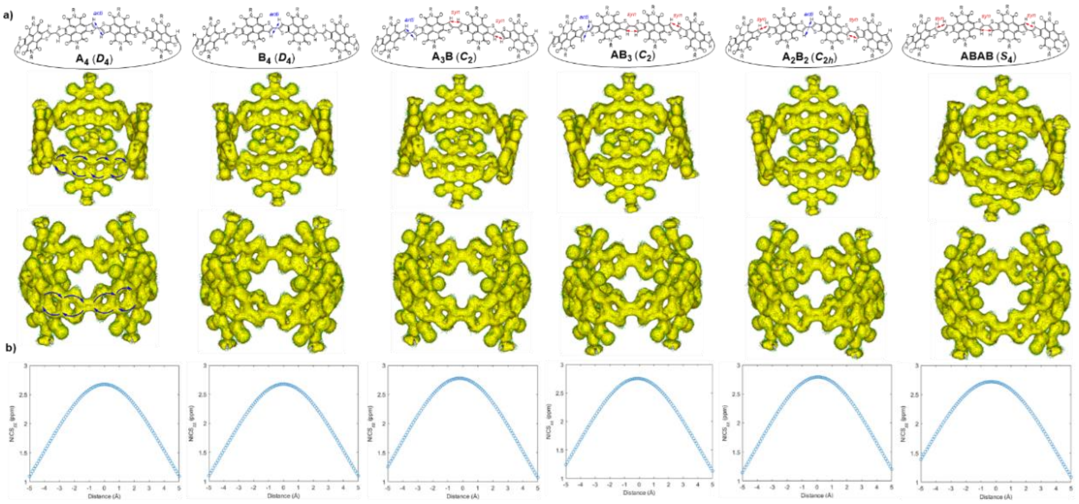

Figure S2. (a) ACID plots (isovalue = 0.032) calculated at CAM-B3LYP/6-311++G(d,p) level and (b) NICS$_{zz}$-scan contour plots (in ppm) calculated at the CAM-B3LYP/6-31+G(d) level for **A_4**, **A_2B_2**, **A_3B**, **AB_3**, **ABAB**, and **B_4**. The NICS scans were performed ranging from –5.0 Å to +5.0 Å relative to the molecular median plane.

Table S2. The NICS(-5 ~ +5)$_{ZZ}$ (ppm) values for the NICS scans of **A_4**, **A_2B_2**, **A_3B**, **AB_3**, **ABAB**, and **B_4**. Calculations were performed at the CAM-B3LYP/6-31+G(d) level. The NICS scans were performed ranging from –5.0 Å to +5.0 Å relative to the molecular median plane. The scanning interval length is 0.1 Å, with a total of 101 values.

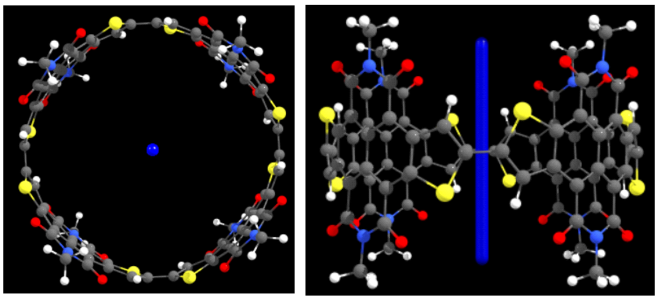

Top view *Side view*

**Entry**	**A_4**	**A_2B_2**	**A_3B**	**AB_3**	**ABAB**	**B_4**
5.0	1.10	1.15	1.26	1.24	1.39	1.10
4.9	1.14	1.19	1.31	1.29	1.43	1.14
4.8	1.18	1.23	1.35	1.33	1.48	1.18
4.7	1.22	1.28	1.40	1.37	1.52	1.23
4.6	1.27	1.32	1.44	1.42	1.57	1.27
4.5	1.31	1.37	1.49	1.46	1.61	1.31
4.4	1.36	1.41	1.53	1.51	1.65	1.36
4.3	1.40	1.46	1.58	1.55	1.69	1.40

4.2	1.44	1.50	1.62	1.59	1.74	1.45
4.1	1.49	1.55	1.67	1.64	1.78	1.49
4.0	1.53	1.59	1.71	1.68	1.82	1.53
3.9	1.58	1.64	1.76	1.73	1.86	1.58
3.8	1.62	1.68	1.80	1.77	1.90	1.62
3.7	1.66	1.73	1.85	1.81	1.94	1.67
3.6	1.71	1.77	1.89	1.86	1.98	1.71
3.5	1.75	1.82	1.93	1.90	2.02	1.75
3.4	1.79	1.86	1.98	1.94	2.06	1.79
3.3	1.83	1.91	2.02	1.98	2.10	1.84
3.2	1.88	1.95	2.06	2.02	2.14	1.88
3.1	1.92	1.99	2.10	2.06	2.17	1.92
3.0	1.96	2.03	2.14	2.10	2.21	1.96
2.9	2.00	2.08	2.18	2.14	2.24	2.00
2.8	2.04	2.12	2.22	2.18	2.28	2.04
2.7	2.08	2.16	2.26	2.22	2.31	2.08
2.6	2.12	2.20	2.30	2.26	2.34	2.12
2.5	2.16	2.24	2.33	2.29	2.37	2.16
2.4	2.19	2.27	2.37	2.33	2.40	2.19
2.3	2.23	2.31	2.40	2.36	2.43	2.23
2.2	2.26	2.35	2.43	2.39	2.46	2.27
2.1	2.30	2.38	2.47	2.43	2.48	2.30
2.0	2.33	2.42	2.50	2.46	2.51	2.33
1.9	2.36	2.45	2.53	2.49	2.53	2.36
1.8	2.39	2.48	2.55	2.51	2.55	2.39
1.7	2.42	2.51	2.58	2.54	2.58	2.42
1.6	2.45	2.54	2.60	2.57	2.60	2.45
1.5	2.48	2.57	2.63	2.59	2.61	2.48
1.4	2.50	2.60	2.65	2.61	2.63	2.50
1.3	2.52	2.62	2.67	2.63	2.65	2.53
1.2	2.55	2.65	2.69	2.65	2.66	2.55
1.1	2.57	2.67	2.71	2.67	2.67	2.57
1.0	2.59	2.69	2.72	2.69	2.69	2.59
0.9	2.60	2.71	2.73	2.70	2.70	2.60
0.8	2.62	2.72	2.75	2.71	2.70	2.62
0.7	2.63	2.74	2.76	2.73	2.71	2.63
0.6	2.64	2.75	2.76	2.74	2.72	2.64
0.5	2.65	2.76	2.77	2.74	2.72	2.65
0.4	2.66	2.77	2.77	2.75	2.72	2.66
0.3	2.67	2.78	2.78	2.75	2.72	2.67
0.2	2.67	2.79	2.78	2.76	2.72	2.67
0.1	2.68	2.79	2.78	2.76	2.72	2.68
0.0	2.68	2.79	2.77	2.75	2.71	2.68
-0.1	2.68	2.79	2.77	2.75	2.71	2.68
-0.2	2.67	2.79	2.76	2.75	2.70	2.67
-0.3	2.67	2.79	2.75	2.74	2.69	2.67
-0.4	2.66	2.78	2.74	2.73	2.68	2.66
-0.5	2.65	2.77	2.73	2.72	2.67	2.65
-0.6	2.64	2.77	2.72	2.71	2.66	2.64
-0.7	2.63	2.75	2.70	2.70	2.64	2.63
-0.8	2.62	2.74	2.68	2.68	2.62	2.62
-0.9	2.60	2.73	2.67	2.66	2.61	2.60
-1.0	2.59	2.71	2.65	2.64	2.59	2.58
-1.1	2.57	2.69	2.62	2.62	2.57	2.57
-1.2	2.55	2.67	2.60	2.60	2.54	2.55
-1.3	2.53	2.65	2.57	2.58	2.52	2.52
-1.4	2.50	2.63	2.55	2.56	2.49	2.50

-1.5	2.48	2.60	2.52	2.53	2.47	2.48
-1.6	2.45	2.58	2.49	2.50	2.44	2.45
-1.7	2.42	2.55	2.46	2.47	2.41	2.42
-1.8	2.39	2.52	2.43	2.44	2.38	2.39
-1.9	2.36	2.49	2.39	2.41	2.35	2.36
-2.0	2.33	2.46	2.36	2.38	2.32	2.33
-2.1	2.30	2.43	2.32	2.35	2.29	2.30
-2.2	2.27	2.39	2.29	2.31	2.25	2.26
-2.3	2.23	2.36	2.25	2.27	2.22	2.23
-2.4	2.19	2.32	2.21	2.24	2.18	2.19
-2.5	2.16	2.29	2.17	2.20	2.14	2.16
-2.6	2.12	2.25	2.13	2.16	2.11	2.12
-2.7	2.08	2.21	2.09	2.12	2.07	2.08
-2.8	2.04	2.17	2.05	2.08	2.03	2.04
-2.9	2.00	2.13	2.01	2.04	1.99	2.00
-3.0	1.96	2.09	1.96	2.00	1.95	1.96
-3.1	1.92	2.05	1.92	1.96	1.91	1.92
-3.2	1.88	2.00	1.88	1.92	1.87	1.88
-3.3	1.84	1.96	1.83	1.88	1.82	1.83
-3.4	1.79	1.92	1.79	1.83	1.78	1.79
-3.5	1.75	1.88	1.74	1.79	1.74	1.75
-3.6	1.71	1.83	1.70	1.75	1.70	1.71
-3.7	1.67	1.79	1.66	1.70	1.65	1.66
-3.8	1.62	1.74	1.61	1.66	1.61	1.62
-3.9	1.58	1.70	1.56	1.62	1.57	1.58
-4.0	1.53	1.65	1.52	1.57	1.52	1.53
-4.1	1.49	1.61	1.47	1.53	1.48	1.49
-4.2	1.45	1.56	1.43	1.48	1.44	1.44
-4.3	1.40	1.52	1.38	1.44	1.39	1.40
-4.4	1.36	1.47	1.34	1.40	1.35	1.36
-4.5	1.31	1.43	1.29	1.35	1.31	1.31
-4.6	1.27	1.38	1.25	1.31	1.26	1.27
-4.7	1.23	1.34	1.20	1.26	1.22	1.22
-4.8	1.18	1.29	1.16	1.22	1.18	1.18
-4.9	1.14	1.25	1.12	1.18	1.13	1.14
-5.0	1.10	1.20	1.07	1.13	1.09	1.10

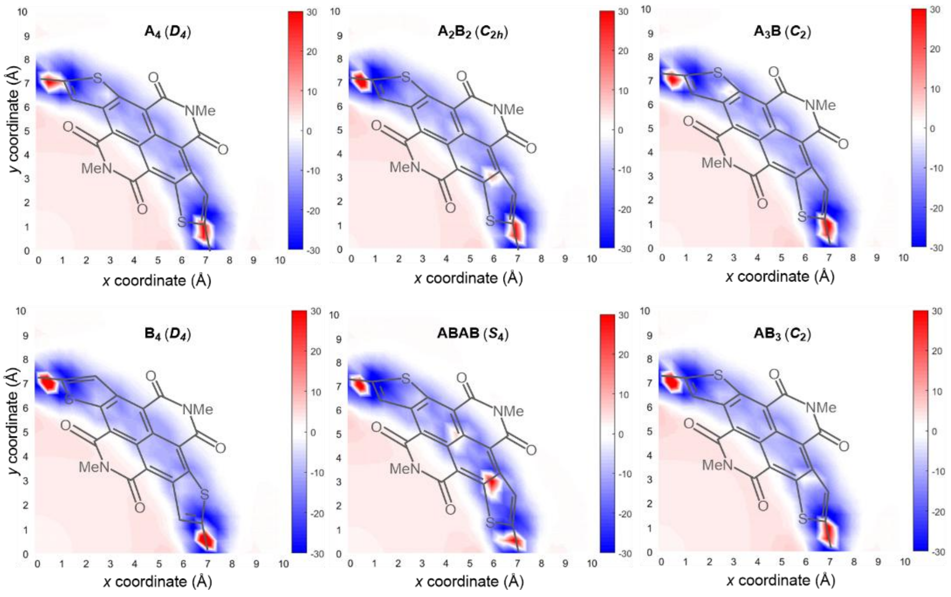

Figure S3. Contour plots visualizing the spatial distribution of NICS-grid (in ppm) for $\mathbf{A_4}$, $\mathbf{A_2B_2}$, $\mathbf{A_3B}$, $\mathbf{AB_3}$, **ABAB**, and $\mathbf{B_4}$ in the cross-sectional *x*-*y* plane of the nanopillar molecule. The color scale represents the computed magnetic shielding strength along the cylindrical axis (*z*-direction). Calculations were performed at the CAM-B3LYP/6-31+G(d) level.

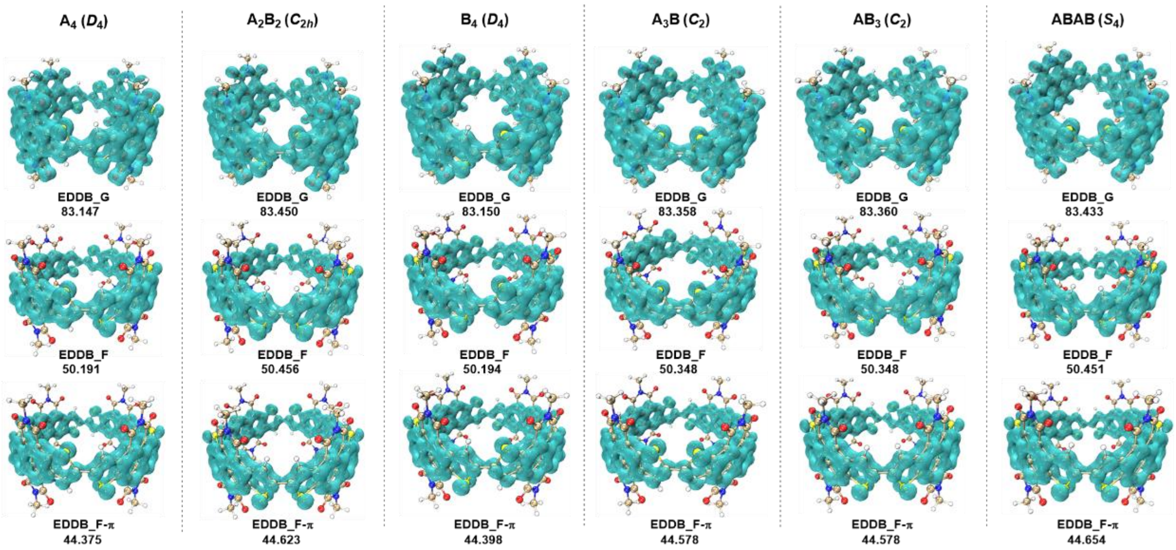

Figure S4. Electron density of delocalized bonds (EDDB) analysis for $\mathbf{A_4}$, $\mathbf{A_2B_2}$, $\mathbf{A_3B}$, $\mathbf{AB_3}$, **ABAB**, and $\mathbf{B_4}$. Corresponding delocalized electron counts (isovalue = 0.015 a.u.) are provided. (EDDB_G: total delocalized electrons, all atoms included; EDDB_F: delocalized electrons on the heavy-atom framework, H excluded; EDDB_F-π: π-delocalized electrons on the heavy-atom framework).

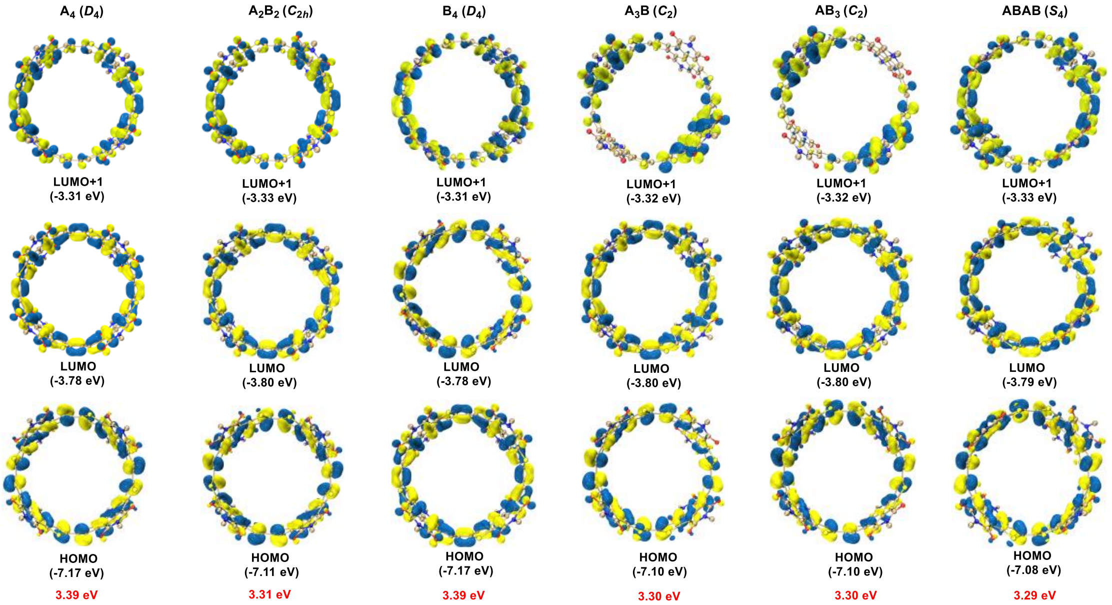

Figure S5. Frontier molecular orbitals and LUMO+1 for $\mathbf{A_4}$, $\mathbf{A_2B_2}$, $\mathbf{A_3B}$, $\mathbf{AB_3}$, **ABAB**, and $\mathbf{B_4}$ (isovalue = 0.02). Orbital energies (in parentheses) and the HOMO-LUMO gaps (highlighted in red) are provided.

Table S3. Relative energies (kcal·mol^{-1}) of $\mathbf{A_4}^{\bullet-}$, $\mathbf{A_2B_2}^{\bullet-}$, $\mathbf{A_3B}^{\bullet-}$, $\mathbf{AB_3}^{\bullet-}$, $\mathbf{ABAB}^{\bullet-}$, and $\mathbf{B_4}^{\bullet-}$. The basis set: (U)M06-2X-(D3)/6-31+G(d).

Compound Name	$A_4^{•–}$	$A_2B_2^{•–}$	$A_3B^{•–}$	$AB_3^{•–}$	$ABAB^{•–}$	$B_4^{•–}$
Relative Energy	0.0	+4.0	+3.9	+3.9	+7.5	0.0

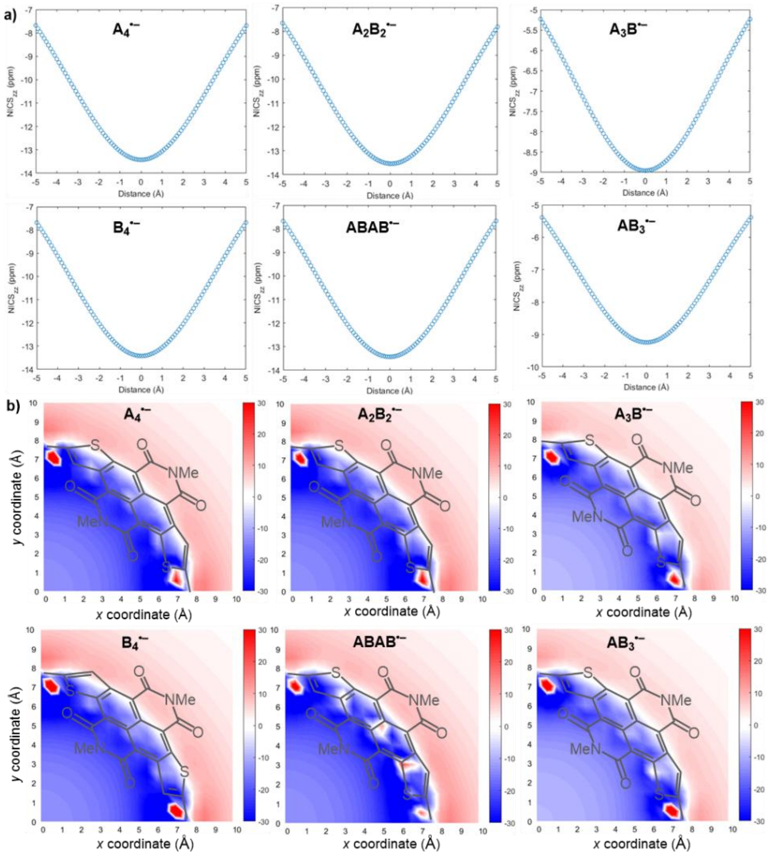

Figure S6. (a) NICS-scan contour plots and (b) contour plots visualizing the spatial distribution of NICS-grid of $\mathbf{A_4^{•–}}$, $\mathbf{A_2B_2^{•–}}$, $\mathbf{A_3B^{•–}}$, $\mathbf{AB_3^{•–}}$, $\mathbf{ABAB^{•–}}$, and $\mathbf{B_4^{•–}}$ in the cross-sectional *x-y* plane of the nanopillar molecules. The color scale represents the computed magnetic shielding strength along the cylindrical axis (*z*-direction). Calculations were performed at the CAM-B3LYP/6-31+G(d) level.

Table S4. The NICS(-5 ~ +5)$_{ZZ}$ (ppm) values for the NICS scans of $\mathbf{A_4^{•–}}$, $\mathbf{A_2B_2^{•–}}$, $\mathbf{A_3B^{•–}}$, $\mathbf{AB_3^{•–}}$, $\mathbf{ABAB^{•–}}$, and $\mathbf{B_4^{•–}}$. Calculations were performed at the CAM-B3LYP/6-31+G(d) level. The NICS scans were performed ranging from –5.0 Å to +5.0 Å relative to the molecular median plane. The scanning interval length is 0.1 Å, with a total of 101 values.

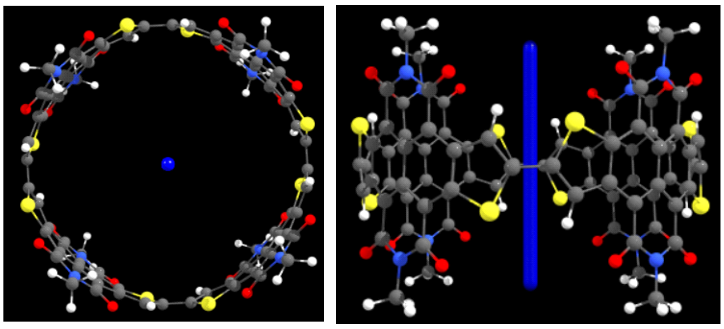

Top view ***Side view***

Entry	$A_4^{•–}$	$A_2B_2^{•–}$	$A_3B^{•–}$	$AB_3^{•–}$	$ABAB^{•–}$	$B_4^{•–}$
5.0	-7.68	-7.66	-5.23	-5.39	-7.67	-7.68
4.9	-7.82	-7.79	-5.32	-5.48	-7.81	-7.82
4.8	-7.95	-7.93	-5.40	-5.57	-7.95	-7.96

4.7	-8.09	-8.07	-5.49	-5.66	-8.09	-8.10
4.6	-8.23	-8.21	-5.58	-5.75	-8.23	-8.24
4.5	-8.38	-8.36	-5.67	-5.85	-8.37	-8.38
4.4	-8.52	-8.50	-5.76	-5.94	-8.52	-8.52
4.3	-8.67	-8.65	-5.86	-6.04	-8.66	-8.67
4.2	-8.82	-8.80	-5.95	-6.14	-8.81	-8.82
4.1	-8.96	-8.95	-6.05	-6.23	-8.96	-8.97
4.0	-9.11	-9.10	-6.14	-6.33	-9.11	-9.12
3.9	-9.27	-9.25	-6.24	-6.43	-9.26	-9.27
3.8	-9.42	-9.40	-6.34	-6.53	-9.41	-9.42
3.7	-9.57	-9.55	-6.44	-6.64	-9.57	-9.57
3.6	-9.72	-9.71	-6.53	-6.74	-9.72	-9.72
3.5	-9.88	-9.86	-6.63	-6.84	-9.87	-9.88
3.4	-10.03	-10.02	-6.73	-6.94	-10.03	-10.03
3.3	-10.18	-10.17	-6.83	-7.05	-10.18	-10.18
3.2	-10.33	-10.33	-6.93	-7.15	-10.33	-10.34
3.1	-10.49	-10.48	-7.03	-7.25	-10.49	-10.49
3.0	-10.64	-10.64	-7.13	-7.35	-10.64	-10.64
2.9	-10.79	-10.79	-7.23	-7.45	-10.79	-10.79
2.8	-10.94	-10.94	-7.32	-7.55	-10.94	-10.94
2.7	-11.08	-11.09	-7.42	-7.65	-11.08	-11.08
2.6	-11.23	-11.24	-7.51	-7.75	-11.23	-11.23
2.5	-11.37	-11.38	-7.61	-7.85	-11.37	-11.37
2.4	-11.51	-11.53	-7.70	-7.94	-11.51	-11.51
2.3	-11.65	-11.67	-7.79	-8.03	-11.65	-11.65
2.2	-11.78	-11.80	-7.88	-8.13	-11.79	-11.78
2.1	-11.92	-11.94	-7.96	-8.21	-11.92	-11.92
2.0	-12.04	-12.07	-8.05	-8.30	-12.05	-12.04
1.9	-12.17	-12.20	-8.13	-8.38	-12.17	-12.17
1.8	-12.29	-12.32	-8.21	-8.47	-12.29	-12.29
1.7	-12.40	-12.44	-8.28	-8.54	-12.40	-12.40
1.6	-12.51	-12.55	-8.35	-8.62	-12.51	-12.51
1.5	-12.62	-12.66	-8.42	-8.69	-12.62	-12.61
1.4	-12.71	-12.76	-8.49	-8.76	-12.72	-12.71
1.3	-12.81	-12.86	-8.55	-8.82	-12.81	-12.81
1.2	-12.90	-12.96	-8.61	-8.88	-12.90	-12.90
1.1	-12.98	-13.04	-8.66	-8.94	-12.98	-12.98
1.0	-13.05	-13.12	-8.71	-8.99	-13.06	-13.05
0.9	-13.12	-13.20	-8.76	-9.04	-13.13	-13.12
0.8	-13.19	-13.26	-8.80	-9.08	-13.19	-13.18
0.7	-13.24	-13.32	-8.83	-9.12	-13.24	-13.24
0.6	-13.29	-13.37	-8.87	-9.15	-13.29	-13.29
0.5	-13.33	-13.42	-8.89	-9.18	-13.33	-13.33
0.4	-13.36	-13.46	-8.92	-9.20	-13.37	-13.36
0.3	-13.39	-13.49	-8.93	-9.22	-13.40	-13.39
0.2	-13.41	-13.51	-8.95	-9.23	-13.41	-13.41
0.1	-13.42	-13.53	-8.95	-9.24	-13.43	-13.42
0.0	-13.43	-13.54	-8.96	-9.24	-13.43	-13.42
-0.1	-13.42	-13.54	-8.95	-9.24	-13.43	-13.42
-0.2	-13.41	-13.53	-8.95	-9.23	-13.41	-13.41
-0.3	-13.39	-13.52	-8.93	-9.22	-13.40	-13.39
-0.4	-13.37	-13.49	-8.92	-9.20	-13.37	-13.36
-0.5	-13.33	-13.46	-8.89	-9.18	-13.34	-13.33
-0.6	-13.29	-13.43	-8.87	-9.15	-13.29	-13.29
-0.7	-13.24	-13.38	-8.83	-9.12	-13.25	-13.24
-0.8	-13.19	-13.33	-8.80	-9.08	-13.19	-13.18
-0.9	-13.13	-13.27	-8.76	-9.04	-13.13	-13.12

-1.0	-13.06	-13.21	-8.71	-8.99	-13.06	-13.05
-1.1	-12.98	-13.13	-8.66	-8.94	-12.98	-12.98
-1.2	-12.90	-13.05	-8.61	-8.88	-12.90	-12.90
-1.3	-12.81	-12.97	-8.55	-8.82	-12.81	-12.81
-1.4	-12.72	-12.88	-8.49	-8.76	-12.72	-12.71
-1.5	-12.62	-12.78	-8.42	-8.69	-12.62	-12.61
-1.6	-12.52	-12.68	-8.35	-8.62	-12.51	-12.51
-1.7	-12.41	-12.57	-8.28	-8.55	-12.40	-12.40
-1.8	-12.29	-12.46	-8.20	-8.47	-12.29	-12.29
-1.9	-12.17	-12.34	-8.13	-8.39	-12.17	-12.17
-2.0	-12.05	-12.22	-8.04	-8.30	-12.05	-12.04
-2.1	-11.92	-12.09	-7.96	-8.22	-11.92	-11.92
-2.2	-11.79	-11.96	-7.87	-8.13	-11.79	-11.78
-2.3	-11.66	-11.82	-7.79	-8.04	-11.65	-11.65
-2.4	-11.52	-11.69	-7.70	-7.94	-11.51	-11.51
-2.5	-11.38	-11.55	-7.61	-7.85	-11.37	-11.37
-2.6	-11.24	-11.40	-7.51	-7.75	-11.23	-11.23
-2.7	-11.09	-11.26	-7.42	-7.65	-11.08	-11.08
-2.8	-10.94	-11.11	-7.32	-7.55	-10.93	-10.94
-2.9	-10.80	-10.96	-7.22	-7.45	-10.79	-10.79
-3.0	-10.65	-10.81	-7.13	-7.35	-10.63	-10.64
-3.1	-10.50	-10.66	-7.03	-7.25	-10.48	-10.49
-3.2	-10.34	-10.51	-6.93	-7.15	-10.33	-10.34
-3.3	-10.19	-10.35	-6.83	-7.05	-10.18	-10.18
-3.4	-10.04	-10.20	-6.73	-6.94	-10.02	-10.03
-3.5	-9.88	-10.04	-6.63	-6.84	-9.87	-9.88
-3.6	-9.73	-9.89	-6.53	-6.74	-9.71	-9.72
-3.7	-9.58	-9.73	-6.44	-6.64	-9.56	-9.57
-3.8	-9.43	-9.58	-6.34	-6.54	-9.41	-9.42
-3.9	-9.27	-9.42	-6.24	-6.43	-9.26	-9.27
-4.0	-9.12	-9.27	-6.14	-6.33	-9.10	-9.12
-4.1	-8.97	-9.12	-6.05	-6.23	-8.95	-8.97
-4.2	-8.82	-8.97	-5.95	-6.14	-8.80	-8.82
-4.3	-8.68	-8.82	-5.86	-6.04	-8.66	-8.67
-4.4	-8.53	-8.67	-5.76	-5.94	-8.51	-8.52
-4.5	-8.39	-8.52	-5.67	-5.85	-8.36	-8.38
-4.6	-8.24	-8.38	-5.58	-5.75	-8.22	-8.24
-4.7	-8.10	-8.23	-5.49	-5.66	-8.08	-8.10
-4.8	-7.96	-8.09	-5.40	-5.57	-7.94	-7.96
-4.9	-7.82	-7.95	-5.32	-5.48	-7.80	-7.82
-5.0	-7.69	-7.82	-5.23	-5.39	-7.67	-7.68

Table S5. Relative energies, the singlet–triplet gap ($\Delta E_{ST} = E_{Triplet} - E_{OSS}$) (kcal·mol^{-1}), and the spin-squared expectation values (<S^2>) for dianions $\mathbf{A_4}^{2-}$, $\mathbf{A_2B_2}^{2-}$, $\mathbf{A_3B}^{2-}$, $\mathbf{AB_3}^{2-}$, $\mathbf{ABAB}^{2-}$, and $\mathbf{B_4}^{2-}$ in the closed-shell singlet (CSS), open-shell singlet (OSS), and triplet (T) states, computed at the M06-2X-(D3)/6-31+G(d) level.

Entry	$\mathbf{A_4}^{2-}$	$\mathbf{A_2B_2}^{2-}$	$\mathbf{A_3B}^{2-}$	$\mathbf{AB_3}^{2-}$	$\mathbf{ABAB}^{2-}$	$\mathbf{B_4}^{2-}$
CSS	0.0	0.0	0.0	0.0	0.0	0.0
**OSS**	**-4.2**	**-4.0**	**-4.1**	**-4.1**	**-4.0**	**-4.2**
$\langle S^2 \rangle_{OSS}$	0.964	0.957	0.960	0.960	0.956	0.964
T	-2.5	-2.2	-2.2	-2.2	-2.1	-2.5

$\langle S^2 \rangle_T$	2.045	2.045	2.045	2.045	2.045	2.045
ΔE_{ST}	1.7	1.8	1.9	1.9	1.9	1.7

Yamaguchi et al. first introduced the parameter y to describe the diradical character of compounds, and defined it as: $y = 2c_2^2$ (Equation 1).[14] Within the framework of spin-projected unrestricted Hartree-Fock (PUHF) theory, another expression for y was derived using the overlap integral (T) between a pair of spatially separated α and β orbitals: $y = 1 - (2T)/(1+ T^2)$ (Equation 2).[15] The orbital overlap integral is directly correlated with natural orbital occupation numbers. Based on the relationships: $n^{PU}_{HONO\text{-}i} = 2 - y_i^{PU}$ (Equation 3) and $n^{PU}_{LUNO+i} = y_i^{PU}$ (Equation 4). After mathematical simplification, Nakano et al. finally established the expression for diradical character based on natural orbital occupation numbers: $1 - y_0 = \frac{n(HONO)-n(LUNO)}{2}$ (Equation 5).[16] This formula is mathematically equivalent to Yamaguchi's original definition using configuration coefficients. It largely resolves the challenge of extracting configuration coefficients (c_1/c_2) for large conjugated molecular systems and thus possesses better computational practicability in practical quantum chemical calculations.

Table S6. Diradical indices (y_0) of compounds $\mathbf{A_4^{2-}}$, $\mathbf{A_2B_2^{2-}}$, $\mathbf{A_3B^{2-}}$, $\mathbf{AB_3^{2-}}$, $\mathbf{ABAB^{2-}}$, and $\mathbf{B_4^{2-}}$ calculated based on natural orbital occupation numbers. The closer the value of y_0 is to 1, the more pronounced the diradical character of the target molecule.

Entry	$\mathbf{A_4^{2-}}$	$\mathbf{A_2B_2^{2-}}$	$\mathbf{A_3B^{2-}}$	$\mathbf{AB_3^{2-}}$	$\mathbf{ABAB^{2-}}$	$\mathbf{B_4^{2-}}$
y_0 [a]	0.68	0.65	0.65	0.65	0.64	0.66

[a] $y_0 = 1 - \frac{n(HONO)-n(LUNO)}{2}$, n(HONO) and n(LUNO) represent the occupation numbers of the highest occupied natural orbital (HONO) and the lowest unoccupied natural orbital (LUNO), respectively.

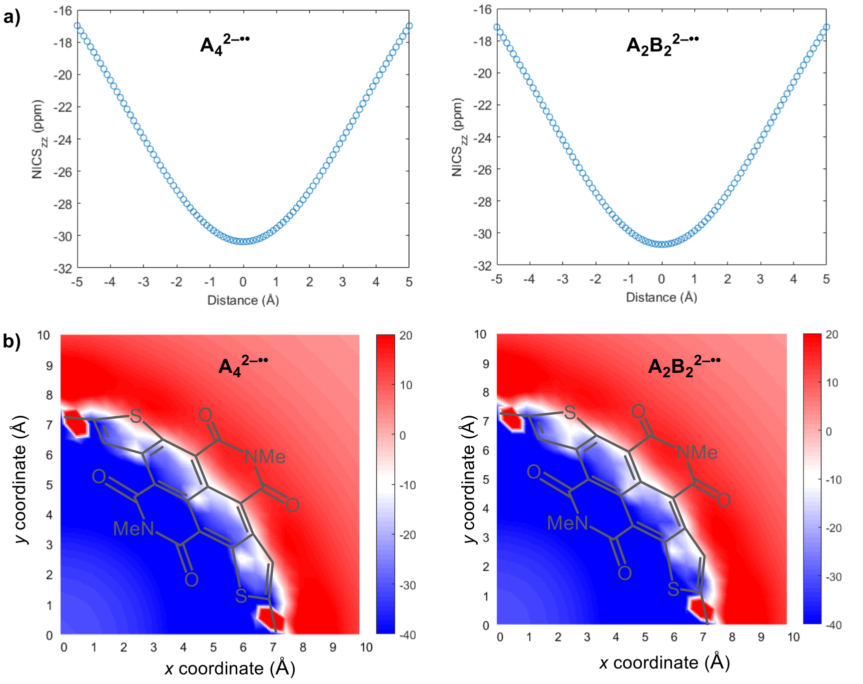

Figure S7. (a) NICS-scan contour plots and (b) contour plots visualizing the spatial distribution of

NICS-grid of $\mathbf{A_4^{2-\bullet\bullet}}$ and $\mathbf{A_2B_2^{2-\bullet\bullet}}$ in the cross-sectional *x-y* plane of the nanopillar molecules. The color scale represents the computed magnetic shielding strength along the cylindrical axis (*z*-direction). Calculations were performed at the (U)CAM-B3LYP/6-31+G(d) level.

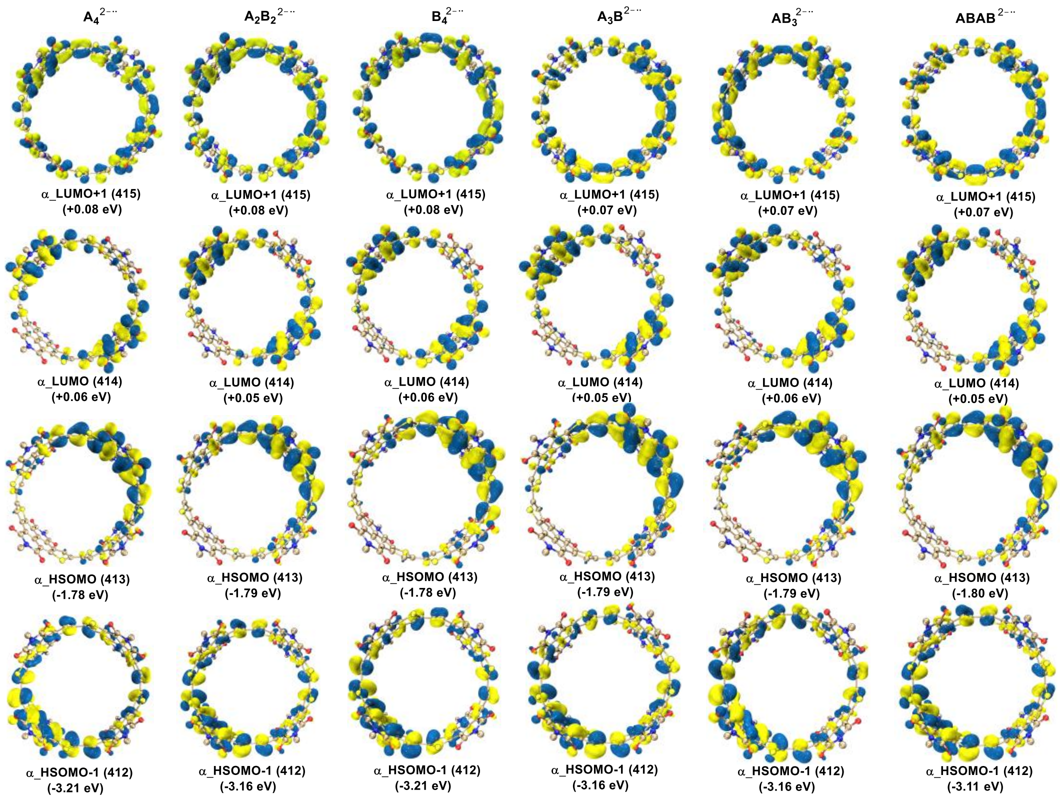

Figure S8. Highest singly occupied molecular orbital (HSOMO), lowest unoccupied molecular orbital (LUMO), second lowest unoccupied molecular orbital (LUMO+1), and second highest singly occupied molecular orbital (HSOMO-1) for $\mathbf{A_4^{2-\bullet\bullet}}$, $\mathbf{A_2B_2^{2-\bullet\bullet}}$, $\mathbf{B_4^{2-\bullet\bullet}}$, $\mathbf{A_3B^{2-\bullet\bullet}}$, $\mathbf{AB_3^{2-\bullet\bullet}}$, and $\mathbf{ABAB^{2-\bullet\bullet}}$ (isovalue = 0.02 a.u.). The numbers of the corresponding orbitals are given in parentheses within the first column, along with their α or β spin characters.

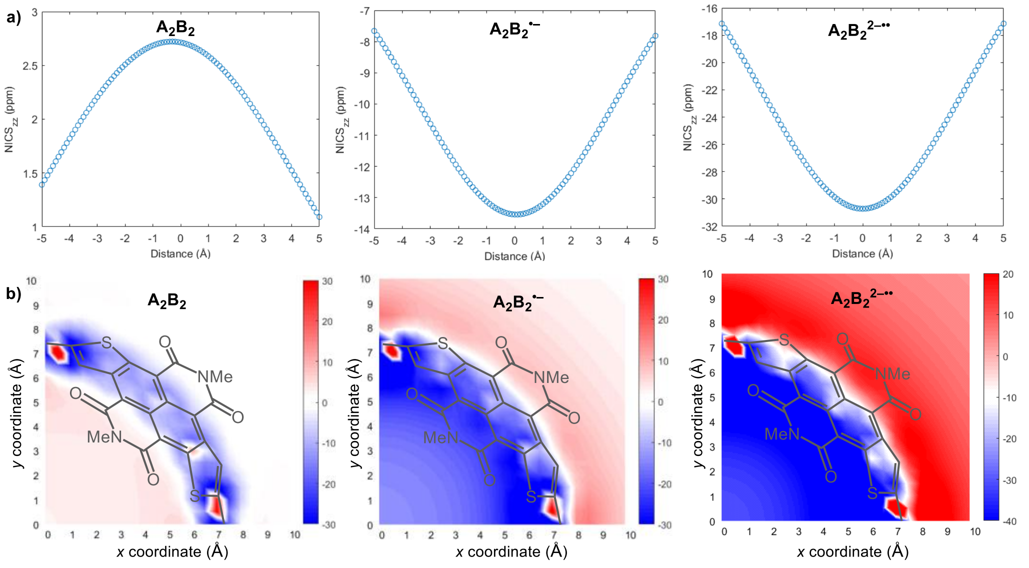

Figure S9. Effect of electron injection on the magnetic shielding properties: (a) NICS-scan contour

plots and (b) contour plots visualizing the spatial distribution of NICS-grid for **A_2B_2**, **$A_2B_2^{\bullet-}$**, and **$A_2B_2^{2-\bullet\bullet}$**. Calculations were performed at the (U)CAM-B3LYP/6-31+G(d) level.

Table S7. The NICS(-5 ~ +5)$_{ZZ}$ (ppm) values for the NICS scans of dianions **A_4^{2-}**, **$A_2B_2^{2-}$**, **A_3B^{2-}**, **AB_3^{2-}**, **$ABAB^{2-}$**, and **B_4^{2-}**. Calculations were performed at the CAM-B3LYP/6-31+G(d) level. The NICS scans were performed ranging from –5.0 Å to +5.0 Å relative to the molecular median plane. The scanning interval length is 0.1 Å, with a total of 101 values.

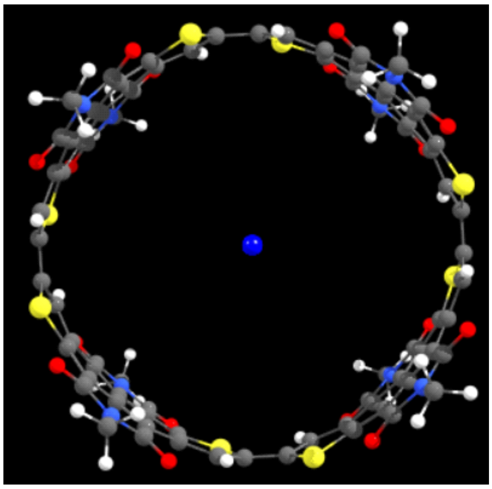

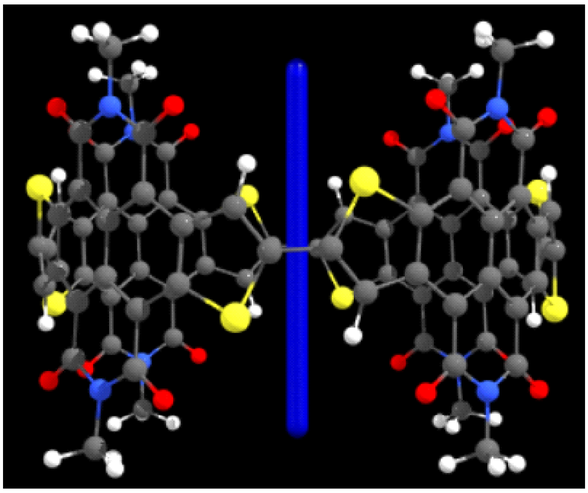

Top view *Side view*

Entry	A_4^{2-}	$A_2B_2^{2-}$	A_3B^{2-}	AB_3^{2-}	$ABAB^{2-}$	B_4^{2-}
5.0	-16.07	-16.30	-16.20	-16.19	-16.29	-16.10
4.9	-16.37	-16.61	-16.50	-16.50	-16.60	-16.40
4.8	-16.68	-16.92	-16.81	-16.81	-16.92	-16.71
4.7	-16.99	-17.24	-17.13	-17.12	-17.23	-17.02
4.6	-17.31	-17.56	-17.45	-17.44	-17.55	-17.34
4.5	-17.63	-17.89	-17.77	-17.76	-17.88	-17.66
4.4	-17.95	-18.21	-18.09	-18.09	-18.21	-17.98
4.3	-18.27	-18.54	-18.42	-18.42	-18.54	-18.31
4.2	-18.60	-18.88	-18.75	-18.75	-18.87	-18.64
4.1	-18.93	-19.21	-19.09	-19.08	-19.21	-18.97
4.0	-19.27	-19.55	-19.42	-19.42	-19.54	-19.30
3.9	-19.60	-19.89	-19.76	-19.76	-19.88	-19.64
3.8	-19.94	-20.24	-20.10	-20.09	-20.23	-19.97
3.7	-20.28	-20.58	-20.44	-20.44	-20.57	-20.31
3.6	-20.61	-20.92	-20.78	-20.78	-20.91	-20.65
3.5	-20.95	-21.26	-21.12	-21.12	-21.26	-20.99
3.4	-21.29	-21.61	-21.46	-21.46	-21.60	-21.32
3.3	-21.63	-21.95	-21.80	-21.80	-21.94	-21.66
3.2	-21.96	-22.29	-22.14	-22.14	-22.29	-22.00
3.1	-22.30	-22.63	-22.48	-22.47	-22.63	-22.33
3.0	-22.63	-22.97	-22.81	-22.81	-22.96	-22.66
2.9	-22.96	-23.30	-23.14	-23.14	-23.30	-22.99
2.8	-23.28	-23.63	-23.47	-23.47	-23.63	-23.32
2.7	-23.61	-23.96	-23.80	-23.79	-23.96	-23.64
2.6	-23.92	-24.28	-24.12	-24.11	-24.28	-23.96
2.5	-24.24	-24.60	-24.43	-24.43	-24.60	-24.27
2.4	-24.54	-24.91	-24.74	-24.74	-24.92	-24.58
2.3	-24.84	-25.22	-25.04	-25.04	-25.22	-24.88
2.2	-25.14	-25.52	-25.34	-25.34	-25.52	-25.17
2.1	-25.42	-25.81	-25.63	-25.63	-25.81	-25.45

2.0	-25.70	-26.09	-25.91	-25.91	-26.10	-25.73
1.9	-25.97	-26.36	-26.18	-26.18	-26.37	-26.00
1.8	-26.23	-26.63	-26.44	-26.44	-26.64	-26.26
1.7	-26.48	-26.88	-26.69	-26.69	-26.89	-26.51
1.6	-26.72	-27.12	-26.93	-26.93	-27.13	-26.75
1.5	-26.94	-27.35	-27.16	-27.16	-27.37	-26.97
1.4	-27.16	-27.57	-27.38	-27.38	-27.59	-27.19
1.3	-27.36	-27.78	-27.58	-27.58	-27.80	-27.39
1.2	-27.55	-27.97	-27.77	-27.77	-27.99	-27.58
1.1	-27.73	-28.15	-27.95	-27.95	-28.17	-27.76
1.0	-27.89	-28.32	-28.12	-28.12	-28.34	-27.92
0.9	-28.04	-28.47	-28.27	-28.27	-28.49	-28.07
0.8	-28.18	-28.61	-28.40	-28.41	-28.63	-28.21
0.7	-28.30	-28.73	-28.52	-28.53	-28.75	-28.33
0.6	-28.40	-28.83	-28.63	-28.63	-28.86	-28.43
0.5	-28.49	-28.93	-28.72	-28.72	-28.95	-28.52
0.4	-28.56	-29.00	-28.79	-28.80	-29.03	-28.59
0.3	-28.62	-29.06	-28.85	-28.85	-29.08	-28.65
0.2	-28.66	-29.10	-28.89	-28.89	-29.13	-28.69
0.1	-28.68	-29.12	-28.91	-28.92	-29.15	-28.71
0.0	-28.69	-29.13	-28.92	-28.93	-29.16	-28.72
-0.1	-28.68	-29.12	-28.91	-28.92	-29.15	-28.71
-0.2	-28.66	-29.10	-28.89	-28.89	-29.13	-28.69
-0.3	-28.62	-29.06	-28.85	-28.85	-29.08	-28.64
-0.4	-28.56	-29.00	-28.79	-28.80	-29.03	-28.59
-0.5	-28.49	-28.93	-28.71	-28.72	-28.95	-28.51
-0.6	-28.40	-28.83	-28.62	-28.63	-28.86	-28.42
-0.7	-28.29	-28.73	-28.52	-28.53	-28.75	-28.32
-0.8	-28.17	-28.61	-28.40	-28.41	-28.63	-28.20
-0.9	-28.04	-28.47	-28.26	-28.27	-28.49	-28.06
-1.0	-27.89	-28.32	-28.11	-28.12	-28.34	-27.91
-1.1	-27.72	-28.15	-27.94	-27.95	-28.17	-27.75
-1.2	-27.54	-27.97	-27.76	-27.77	-27.99	-27.57
-1.3	-27.35	-27.78	-27.57	-27.58	-27.80	-27.38
-1.4	-27.15	-27.57	-27.37	-27.38	-27.59	-27.18
-1.5	-26.93	-27.35	-27.15	-27.16	-27.37	-26.96
-1.6	-26.71	-27.12	-26.92	-26.93	-27.13	-26.73
-1.7	-26.47	-26.88	-26.68	-26.69	-26.89	-26.49
-1.8	-26.22	-26.63	-26.43	-26.44	-26.63	-26.24
-1.9	-25.96	-26.36	-26.16	-26.18	-26.37	-25.98
-2.0	-25.69	-26.09	-25.89	-25.91	-26.10	-25.71
-2.1	-25.41	-25.81	-25.61	-25.63	-25.81	-25.43
-2.2	-25.13	-25.52	-25.32	-25.34	-25.52	-25.15
-2.3	-24.83	-25.22	-25.03	-25.04	-25.22	-24.86
-2.4	-24.53	-24.91	-24.72	-24.74	-24.91	-24.55
-2.5	-24.23	-24.60	-24.42	-24.43	-24.60	-24.25
-2.6	-23.91	-24.28	-24.10	-24.11	-24.28	-23.93
-2.7	-23.60	-23.96	-23.78	-23.79	-23.96	-23.62
-2.8	-23.27	-23.63	-23.46	-23.47	-23.63	-23.29
-2.9	-22.95	-23.30	-23.13	-23.14	-23.30	-22.97
-3.0	-22.62	-22.97	-22.79	-22.81	-22.96	-22.64
-3.1	-22.29	-22.63	-22.46	-22.47	-22.63	-22.31
-3.2	-21.95	-22.29	-22.12	-22.14	-22.28	-21.97
-3.3	-21.62	-21.95	-21.78	-21.80	-21.94	-21.64
-3.4	-21.28	-21.61	-21.44	-21.46	-21.60	-21.30
-3.5	-20.94	-21.26	-21.10	-21.12	-21.26	-20.96
-3.6	-20.60	-20.92	-20.76	-20.78	-20.91	-20.62

-3.7	-20.27	-20.58	-20.42	-20.43	-20.57	-20.28
-3.8	-19.93	-20.24	-20.08	-20.09	-20.23	-19.95
-3.9	-19.59	-19.89	-19.74	-19.76	-19.88	-19.61
-4.0	-19.26	-19.55	-19.41	-19.42	-19.54	-19.28
-4.1	-18.92	-19.21	-19.07	-19.08	-19.21	-18.94
-4.2	-18.59	-18.88	-18.74	-18.75	-18.87	-18.61
-4.3	-18.27	-18.54	-18.41	-18.42	-18.54	-18.28
-4.4	-17.94	-18.21	-18.08	-18.09	-18.20	-17.96
-4.5	-17.62	-17.89	-17.75	-17.76	-17.88	-17.63
-4.6	-17.30	-17.56	-17.43	-17.44	-17.55	-17.31
-4.7	-16.98	-17.24	-17.11	-17.12	-17.23	-17.00
-4.8	-16.67	-16.92	-16.80	-16.81	-16.91	-16.69
-4.9	-16.36	-16.61	-16.49	-16.50	-16.60	-16.38
-5.0	-16.06	-16.30	-16.18	-16.19	-16.29	-16.07

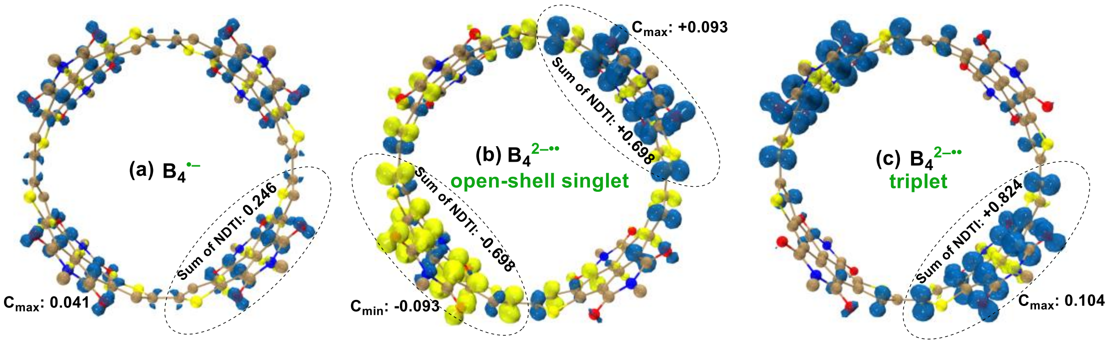

Figure S10. Spin density plots (isovalue = 0.002 a.u.) and the main spin populations for (a) $\mathbf{B_4}^{\bullet-}$, (b) $\mathbf{B_4}^{2-\bullet\bullet}$ (open-shell singlet), and (c) $\mathbf{B_4}^{2-\bullet\bullet}$ (triplet). Hydrogen atoms are omitted for clarity.

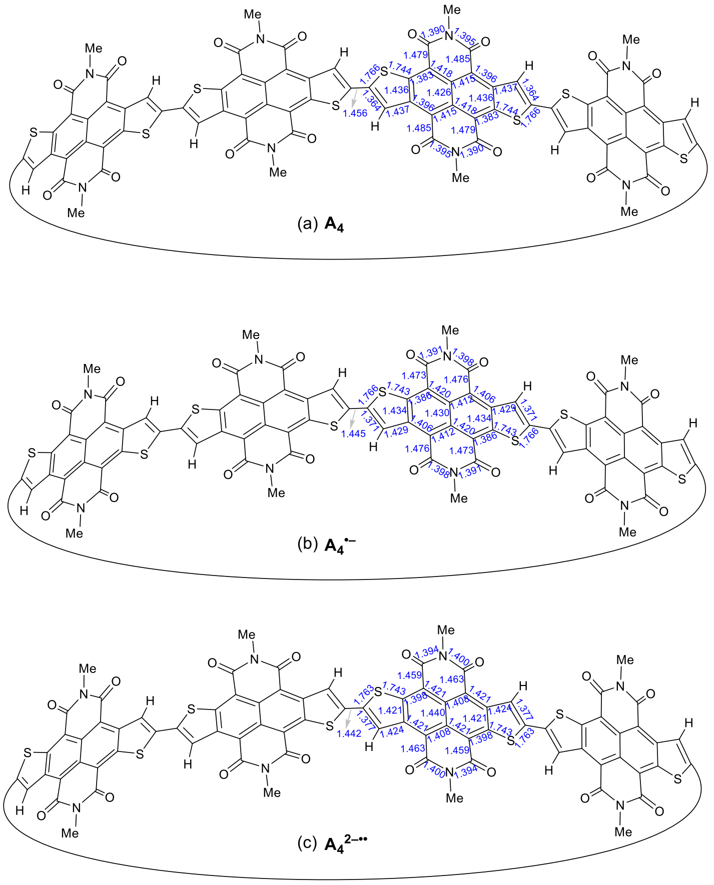

Figure S11. Key bond-lengths (Å) for the individual rings and the inter-unit linkages in $\mathbf{A_4}$, $\mathbf{A_4}^{\bullet-}$, and $\mathbf{A_4}^{2-\bullet\bullet}$.

By comparing the C-C bond lengths within NDTI units among the neutral, monoanionic, and dianionic systems (Figure S11), the results are summarized as follows: 1) The C-C bond length differences of thiophene rings change from 0.073 Å (BL_{max}–BL_{min}: 1.437 –1.364 Å) to 0.063 Å (1.434 – 1.371 Å) and 0.063 Å (1.424 – 1.377 Å); 2) The C-C bond length differences of benzene rings vary from 0.053 Å (1.436 – 1.383 Å) to 0.048 Å (1.434 – 1.386 Å) and 0.042 Å (1.440 – 1.398 Å); 3) The lengths of the C-C bonds linking adjacent NDTI units show a gradual change of 1.456 Å → 1.445 Å → 1.442 Å. These variations clearly reveal the feature of bond length equalization upon reduction, which further corroborates the conclusion of global π-electron delocalization and aromaticity transition of the system.

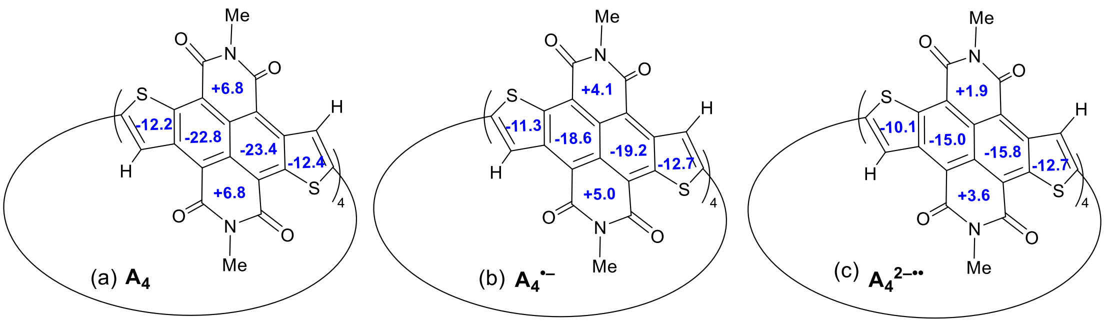

Figure S12. The NICS(1)$_{ZZ}$ values (ppm) of the individual rings in $\mathbf{A_4}$, $\mathbf{A_4}^{\bullet-}$, and $\mathbf{A_4}^{2-\bullet\bullet}$. The values are the average of NICS(+1)$_{ZZ}$ and NICS(-1)$_{ZZ}$.

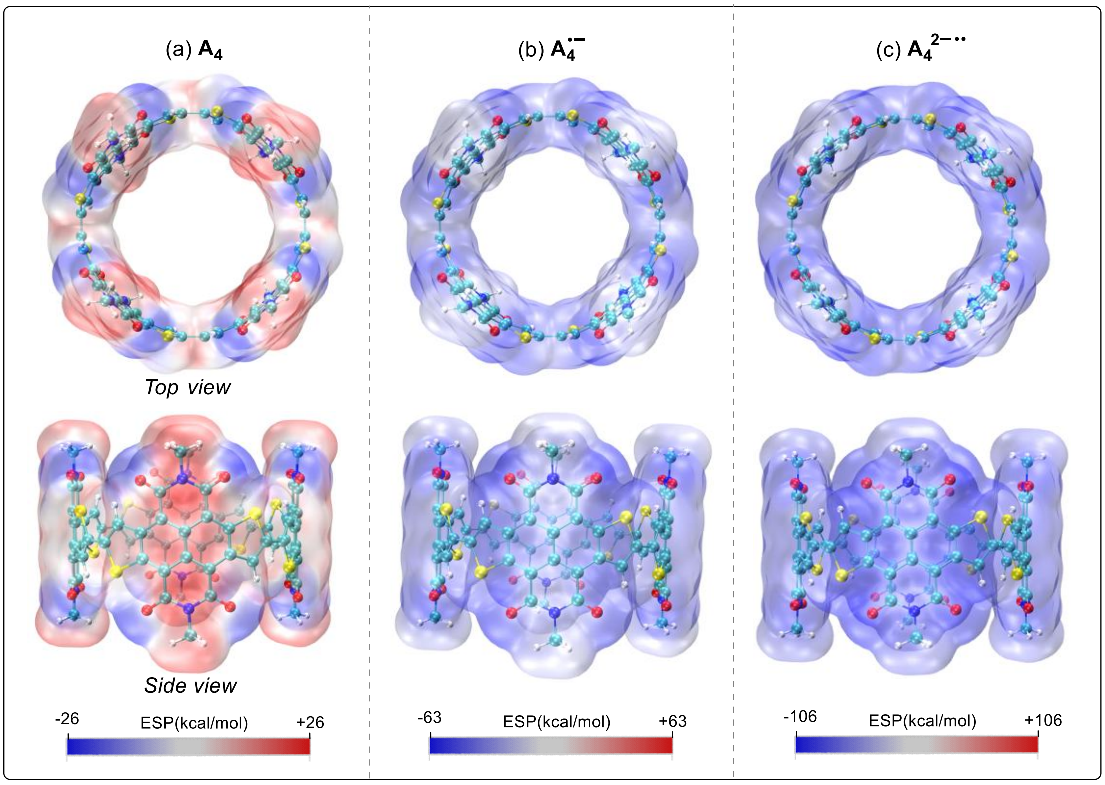

Figure S13. Electrostatic potential (ESP) surfaces of (a) $\mathbf{A_4}$, (b) $\mathbf{A_4}^{\bullet-}$, and (c) $\mathbf{A_4}^{2-\bullet\bullet}$ (isosurface value = 0.001 a.u.). Red and blue regions indicate positive and negative electrostatic potentials, respectively.

As illustrated in the ESP plots: (a) For neutral $\mathbf{A_4}$, positive electrostatic potential (red regions, +26 kcal/mol maximum) is distributed at the periphery of individual NDTI units and along the macrocyclic π-conjugated backbone, whereas negative electrostatic potential (blue regions, minimum −26 kcal/mol) is mainly concentrated around oxygen atom sites. (b) After one-electron reduction to form monoanionic $\mathbf{A}^{\bullet-}$, the overall negative electrostatic potential of the macrocyclic framework is markedly strengthened (minimum -63 kcal/mol), while positive potential at peripheral sites is obviously weakened. (c) Upon further two-electron reduction to dianionic $\mathbf{A_4}^{2-\bullet\bullet}$, negative potential over the whole macrocyclic π-skeleton is further enhanced (minimum -106 kcal/mol). Electrons preferentially accumulate on the global delocalized π-surface of the macrocyclic backbone rather than being confined to single NDTI units during electron injection. This global charge redistribution directly verifies the evolution toward enhanced global aromaticity of the macrocycle upon sequential reduction.

We have also performed comprehensive TD-DFT simulations (Figure S14) for both the monoanionic ($\mathbf{A}^{\bullet-}$) and dianionic ($\mathbf{A_4}^{2-\bullet\bullet}$) systems, along with detailed frontier orbital analysis, to rationalize the origin of the long-wavelength absorption and the dramatic blue shift upon further reduction.

(a) For $\mathbf{A}^{\bullet-}$: The experimental absorption maximum appears at ~2680 nm. TD-DFT calculations predict the corresponding vertical excitation at λ_{cal} = ~2500 nm with an oscillator strength (f) of 0.347, in good agreement with the experimental trend. Dominant electronic transitions: The transition is primarily contributed by the α_HSOMO→α_LUMO excitation (89.6%), with a minor contribution from α_HSOMO-1→α_LUMO+1 (10.4%).

Electronic origin of long-wavelength absorption: The monoanion exhibits a small frontier orbital energy gap of 1.32 eV, which accounts for its low-energy long-wavelength absorption. Upon one-electron reduction, the added electron occupies the LUMO of the neutral molecule and consequently forms a SOMO. After orbital rearrangement, the energy gaps between the HSOMO/HSOMO−1 and LUMO/LUMO+1 are remarkably narrowed. In particular, the α_HSOMO–α_LUMO gap is calculated to be 1.32 eV ($E_{\text{LUMO-HOMO}}$ of neutral $\mathbf{A_4}$ is 3.39 eV in Figure S5). The reduction in vertical excitation energy eventually leads to the observed long-wavelength absorption.

(b) For $\mathbf{A_4}^{2-\bullet\bullet}$: Further reduction to the dianion results in a dramatic blue shift of the absorption maximum to ~1860 nm. TD-DFT simulations reproduce this trend well, with a calculated excitation at λ_{cal} =1721 nm and a significantly increased oscillator strength (f = 0.736). Dominant electronic transitions: The main contribution to this transition is the α_HSOMO→α_LUMO excitation (86.7%), with a minor contribution from α_HSOMO-1→α_LUMO+1 (13.3%).

Electronic origin of the blue shift: The blue shift is directly attributed to the increased vertical excitation energy in the dianion compared to the monoanion. Upon the second electron injection, the frontier orbital energies are rearranged. The dominant allowed transition in the dianion (α_HSOMO→α_LUMO) involves a larger energy gap (1.84 eV) than that in the monoanion (α_HSOMO→α_LUMO, 1.32 eV), based on the DFT-calculated orbital energies (see Fig. S14). This larger excitation energy leads to the observed blue shift.

The higher oscillator strength (f = 0.736 vs. f = 0.347 for the monoanion) further indicates a more fully allowed transition, consistent with the enhanced global π-delocalization and aromatic character in the dianionic state.

These TD-DFT results provide a clear electronic picture of the spectral evolution: the low-energy absorption in the monoanion arises from a narrow frontier orbital gap, while the second reduction

increases the excitation energy of the dominant transition, leading to the blue shift.

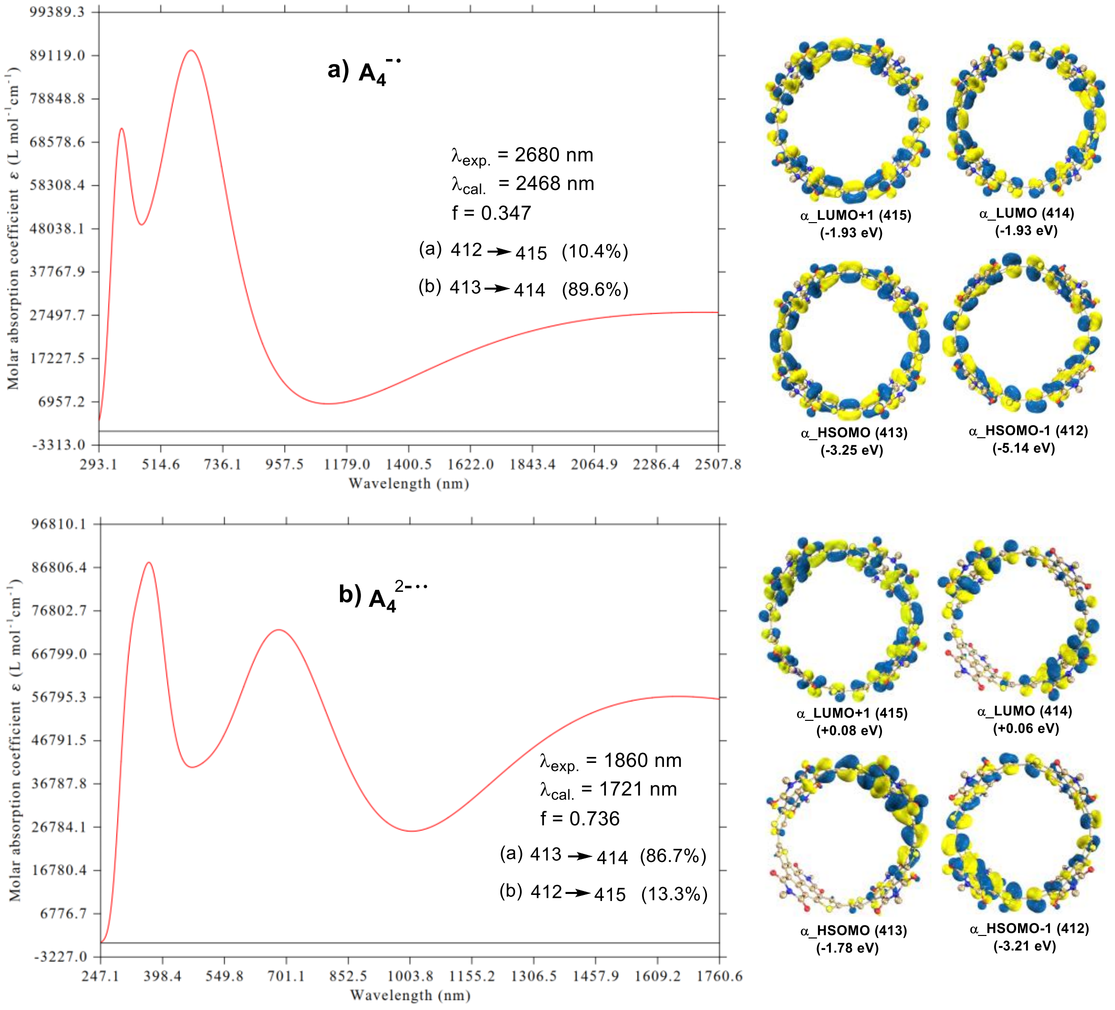

Figure S14. TD-DFT simulations for both the monoanionic and dianionic systems. Including selected Transition wavelength, and oscillator strengths of the electronic transition of $\mathbf{A}^{\bullet-}$ (a) and $\mathbf{A}_4^{2-\bullet\bullet}$ (b) at the M06-2X/6-311G(d,p)~(solvent=Dichloromethane) level of theory.

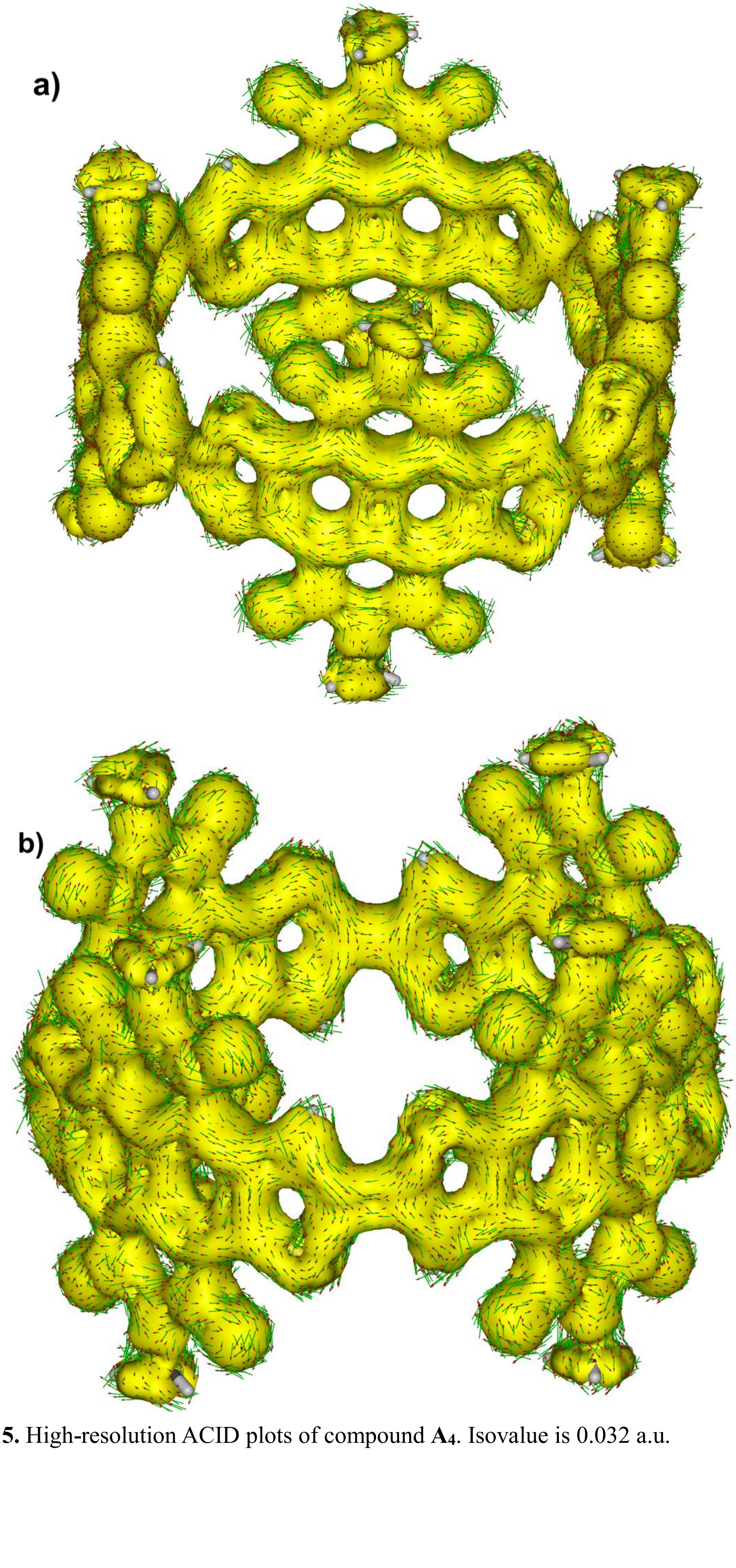

Figure S15. High-resolution ACID plots of compound $\mathbf{A_4}$. Isovalue is 0.032 a.u.

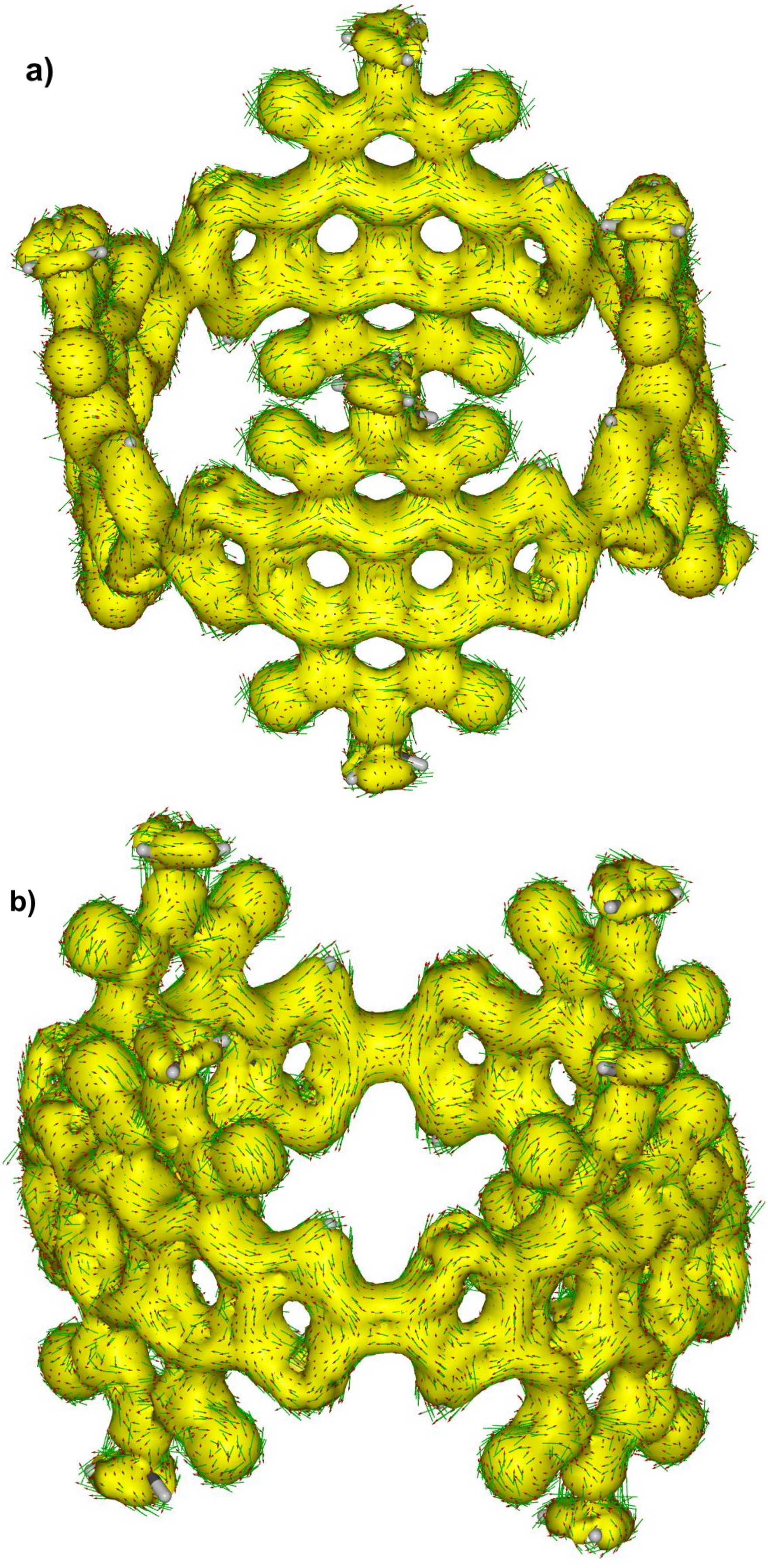

Figure S16. High-resolution ACID plots of compound $\mathbf{A_2B_2}$. Isovalue is 0.032 a.u.

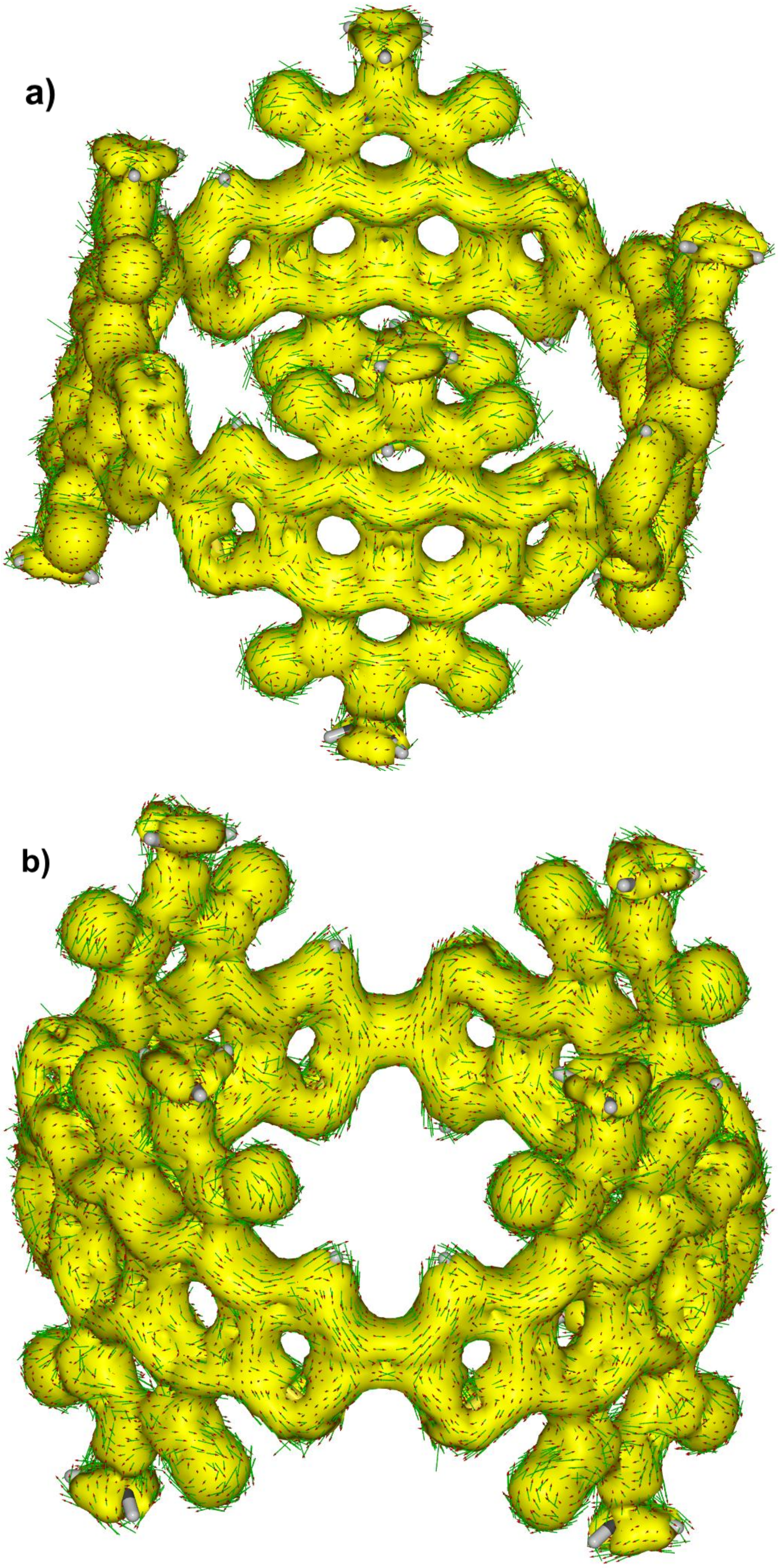

Figure S17. High-resolution ACID plots of compound $\mathbf{A_3B}$. Isovalue is 0.032 a.u.

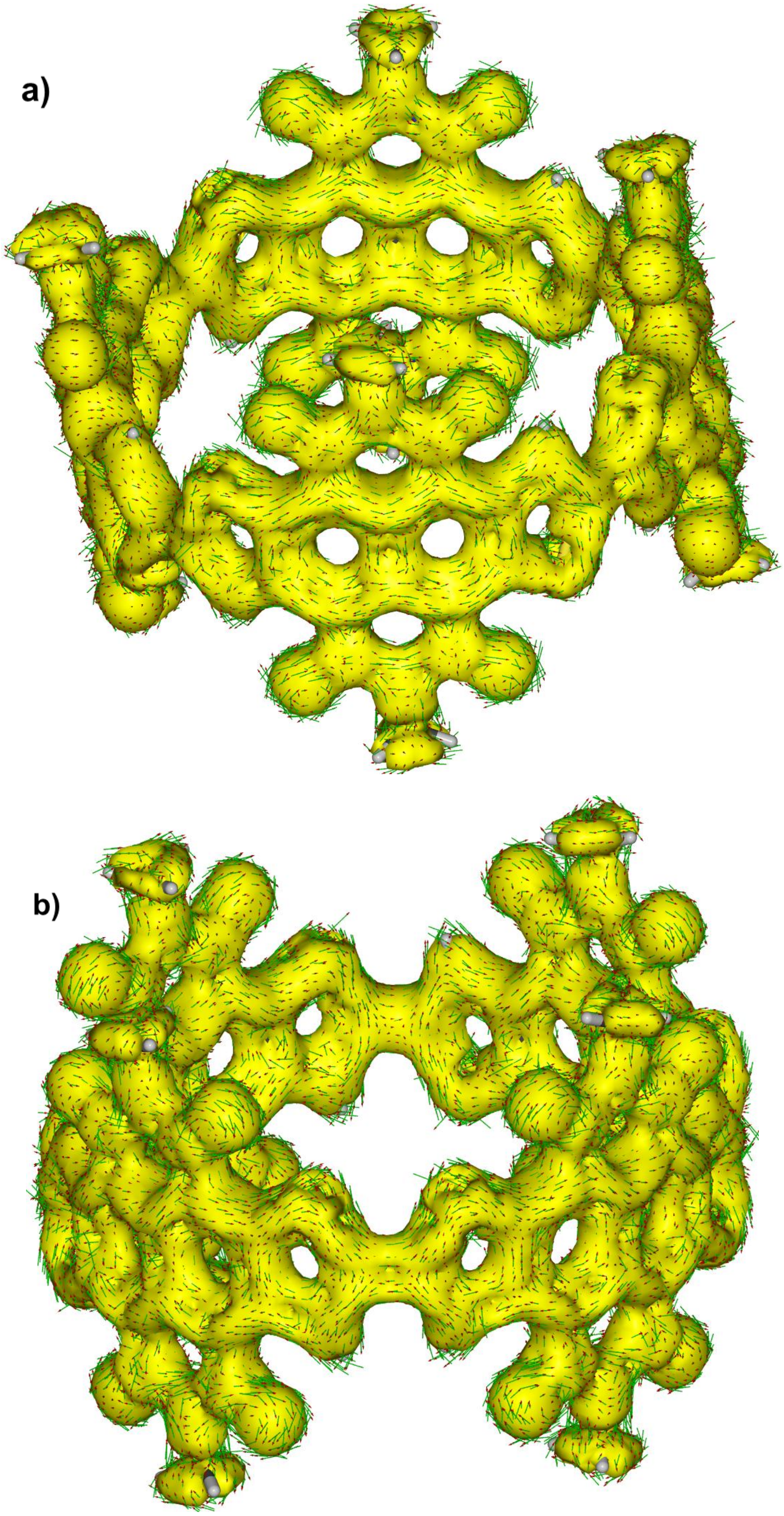

Figure S18. High-resolution ACID plots of compound **AB_3**. Isovalue is 0.032 a.u.

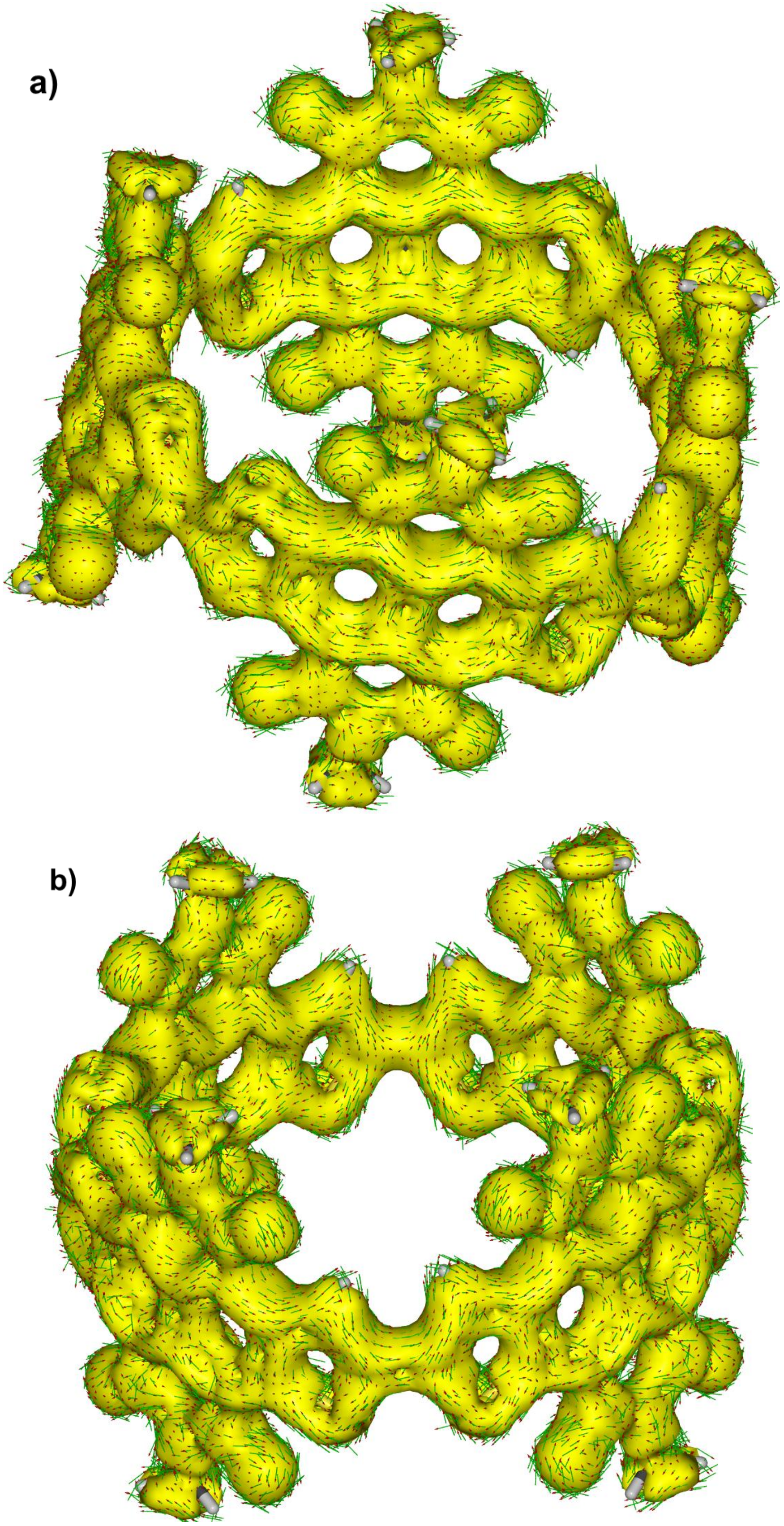

Figure S19. High-resolution ACID plots of compound **ABAB**. Isovalue is 0.032 a.u.

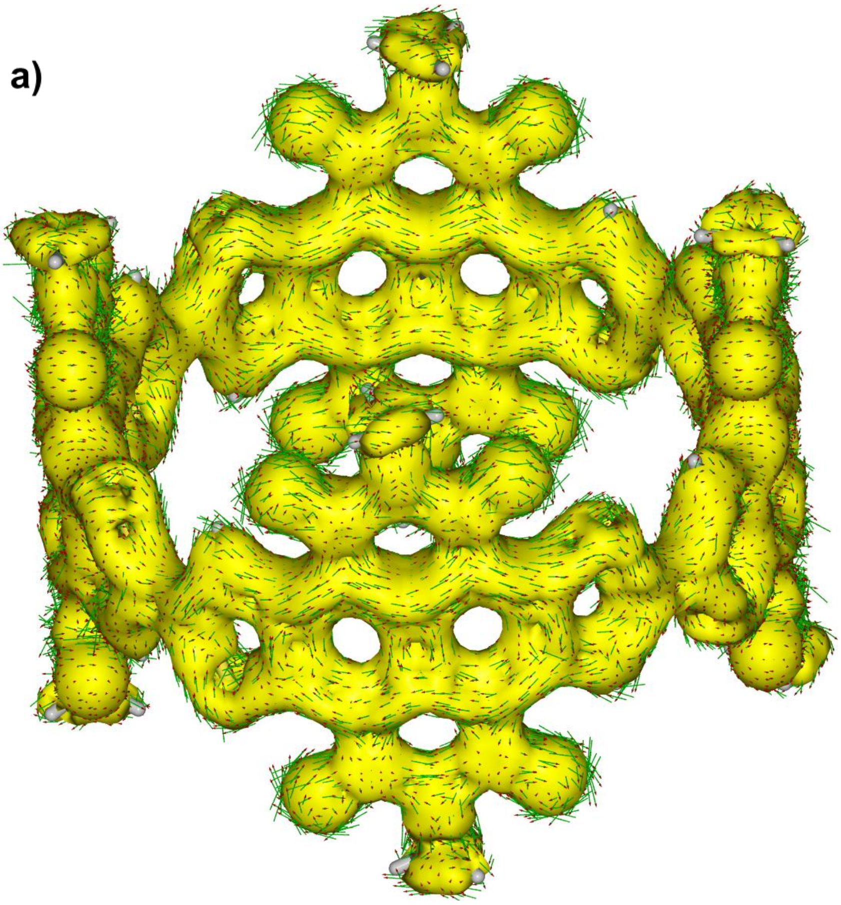

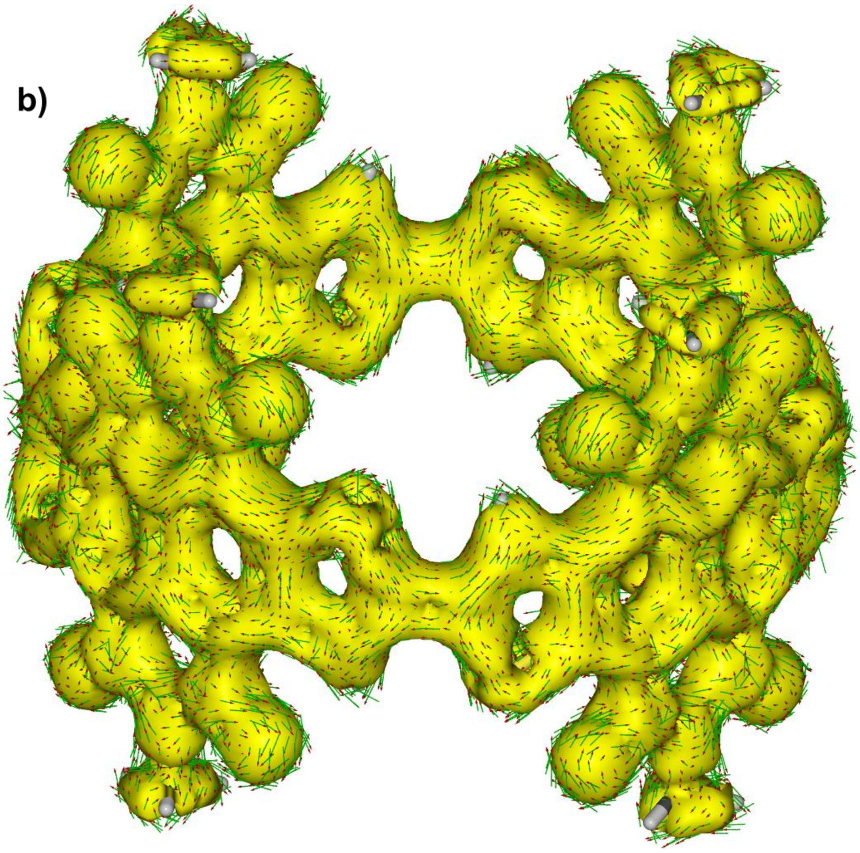

Figure S20. High-resolution ACID plots of compound $\mathbf{B_4}$. Isovalue is 0.032 a.u.

a)

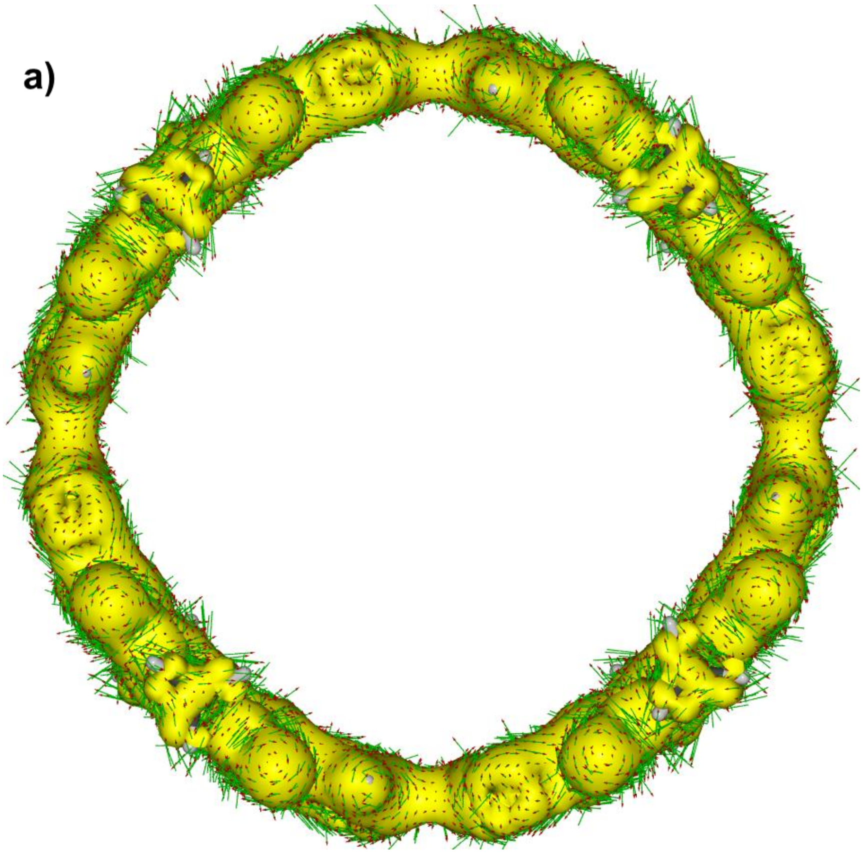

b)

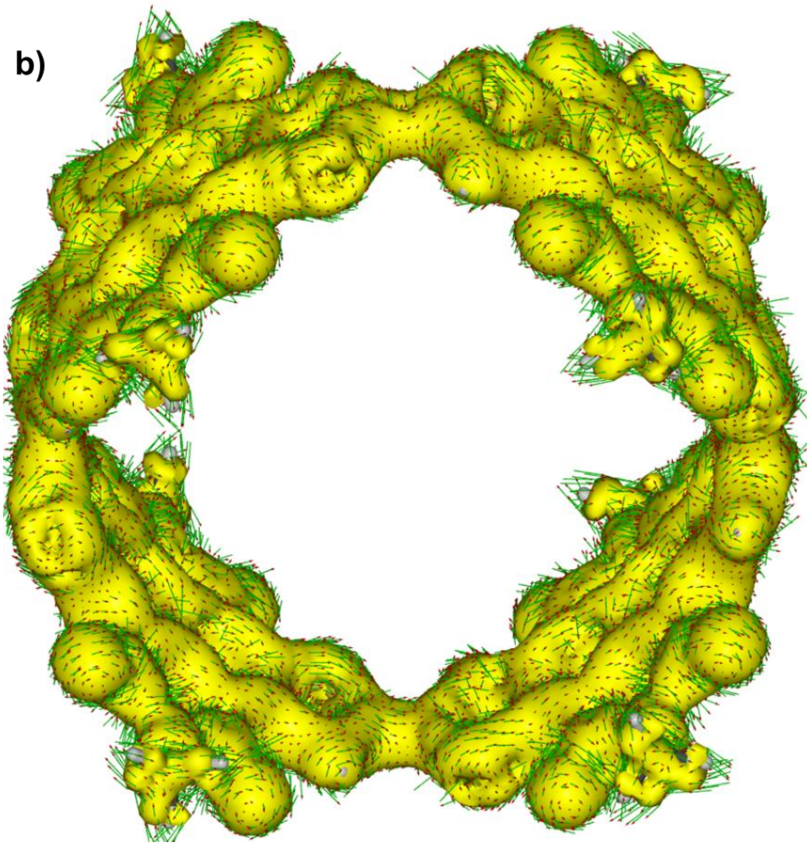

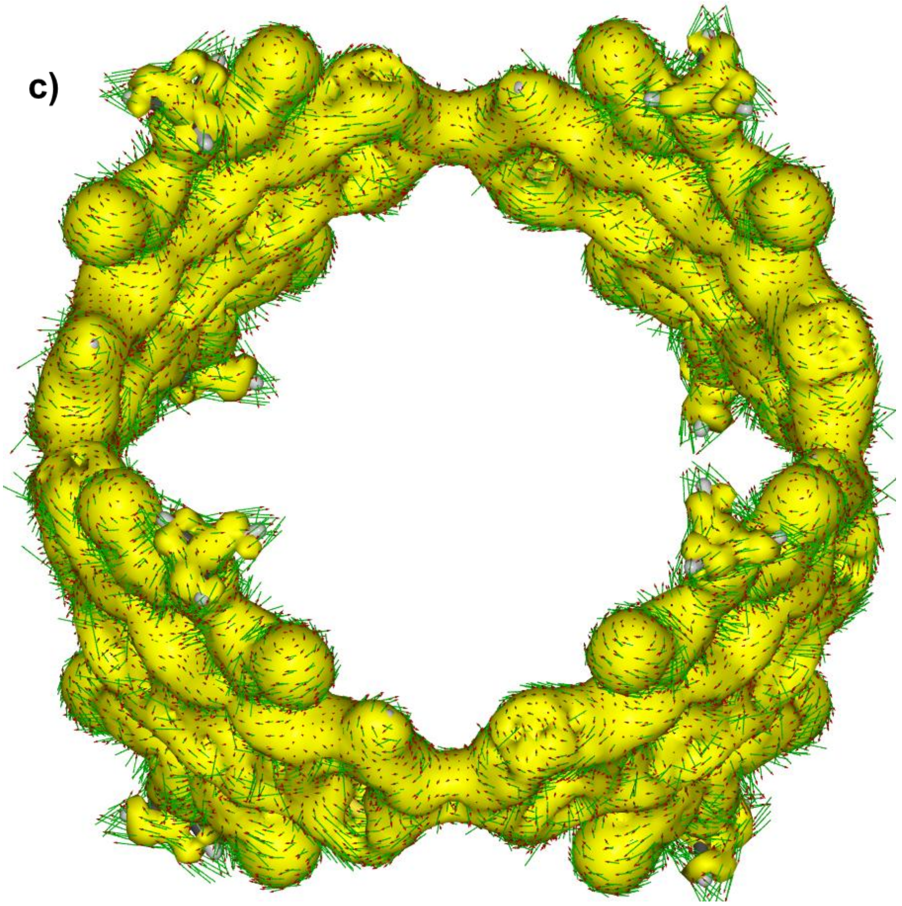

Figure S21. High-resolution ACID plots of compound $\mathbf{A_4}$ viewed from different angles. Isovalue is 0.032 a.u.

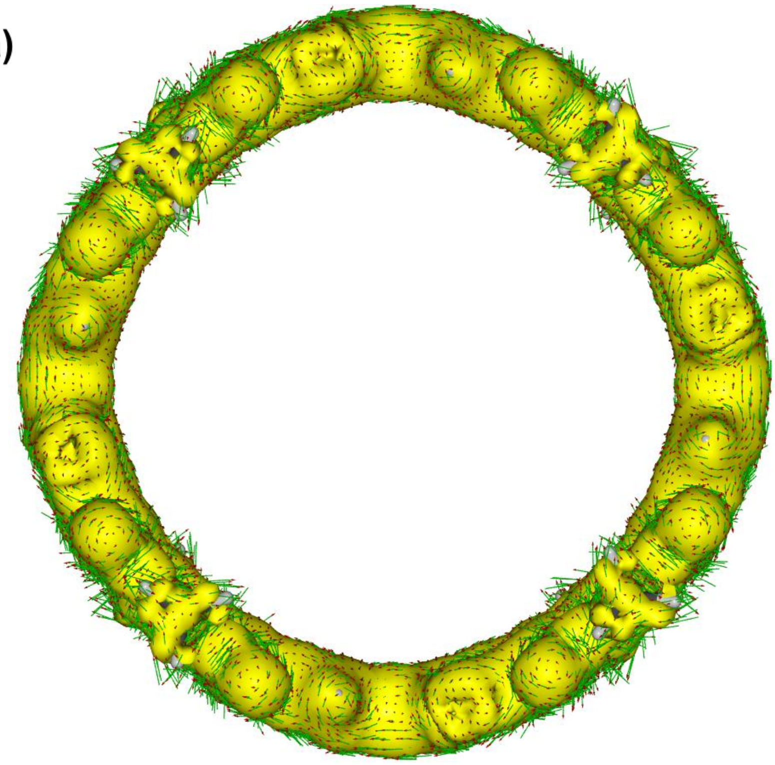

b)

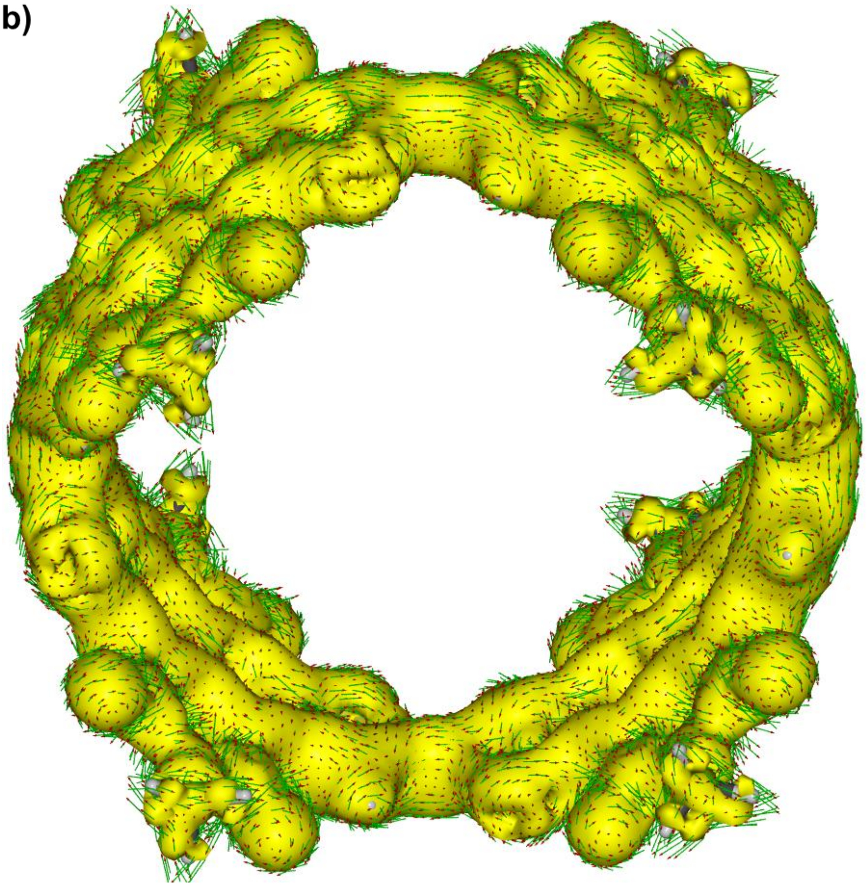

c)

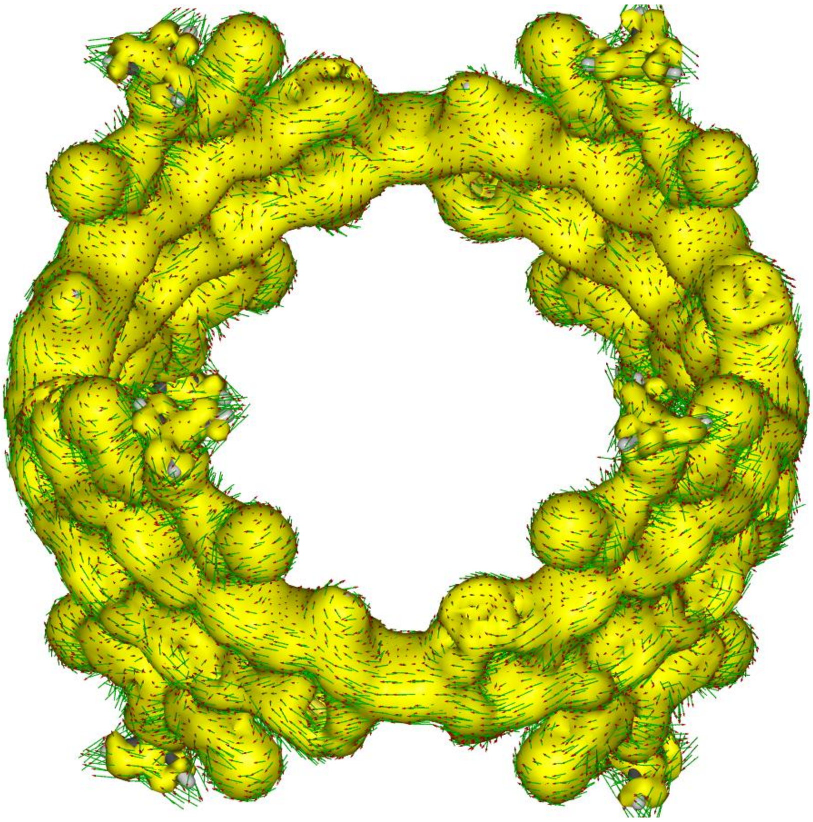

Figure S22. High-resolution ACID plots of compound $\mathbf{A_4}^{\bullet-}$ viewed from different angles. Isovalue is 0.032 a.u.

a)

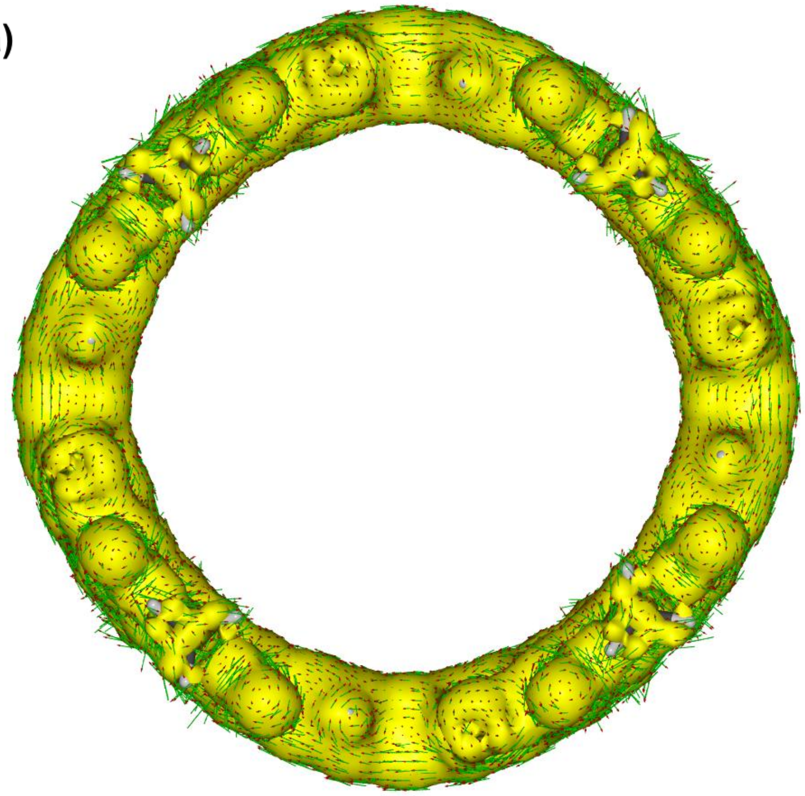

b)

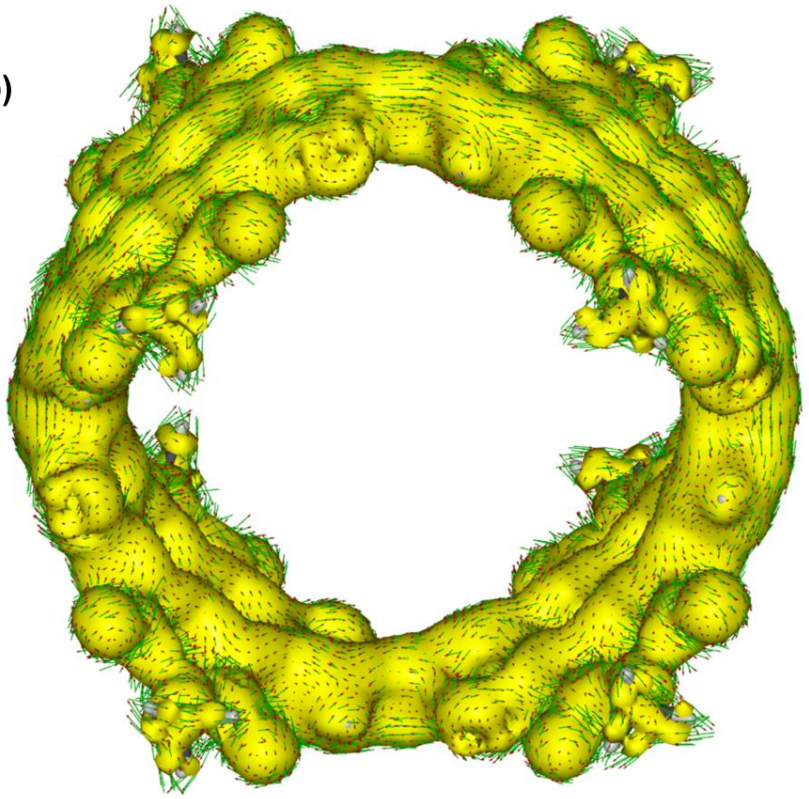

c)

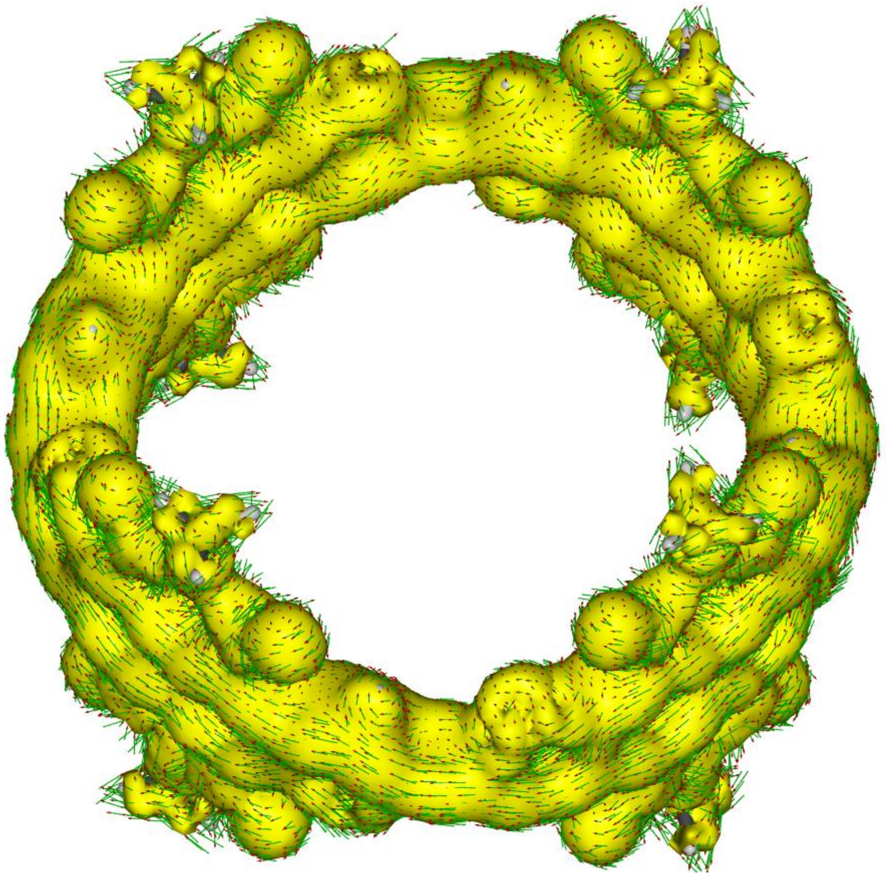

Figure S23. High-resolution ACID plots of compound $\mathbf{A_4}^{2-\bullet\bullet}$ viewed from different angles. Isovalue is 0.032 a.u.

III. Electrochemical Properties

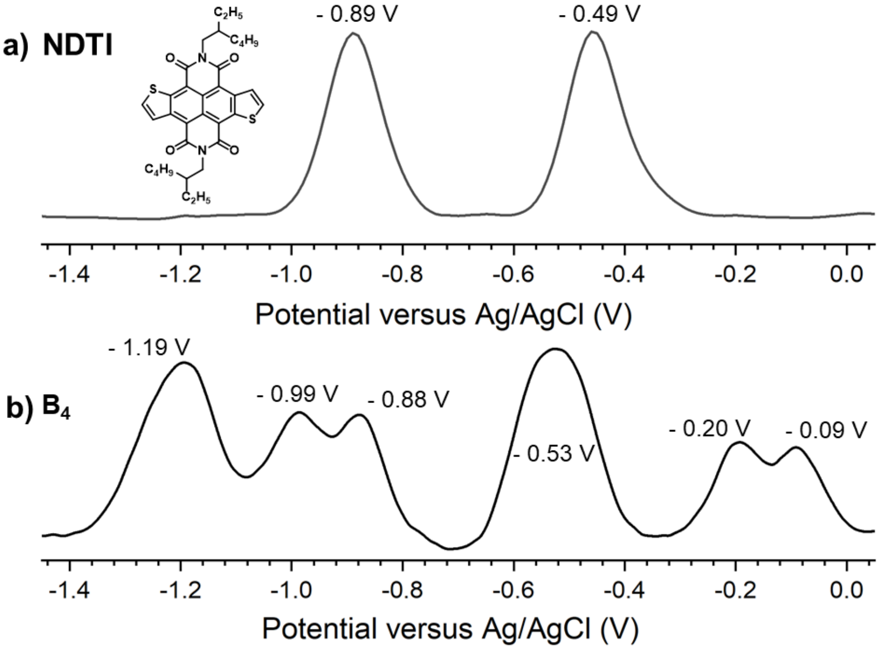

Figure S24. Square wave voltammetry (SWV) of **NDTI** and **B_4** in DCM (0.1 M Bu_4NPF_6). Scan rate: 20 mV s^{-1}. Electrodes: Pt working and counter, Ag/AgCl reference.

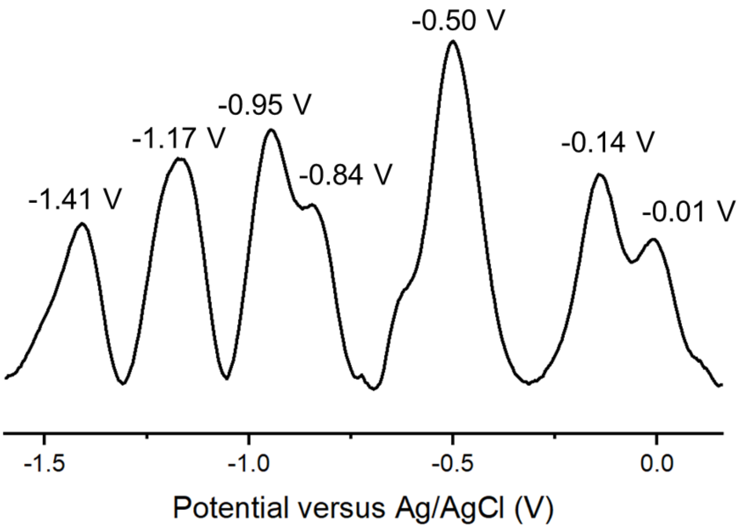

Figure S25. Square wave voltammetry (SWV) of **$B_4{\supset}C_{60}$** in DCM (0.1 M Bu_4NPF_6). Scan rate: 20 mV s^{-1}. Electrodes: Pt working and counter, Ag/AgCl reference.

IV. UV-Vis-NIR Absorption Spectra: Chemical and Electrochemical Reduction

1) Chemical reduction

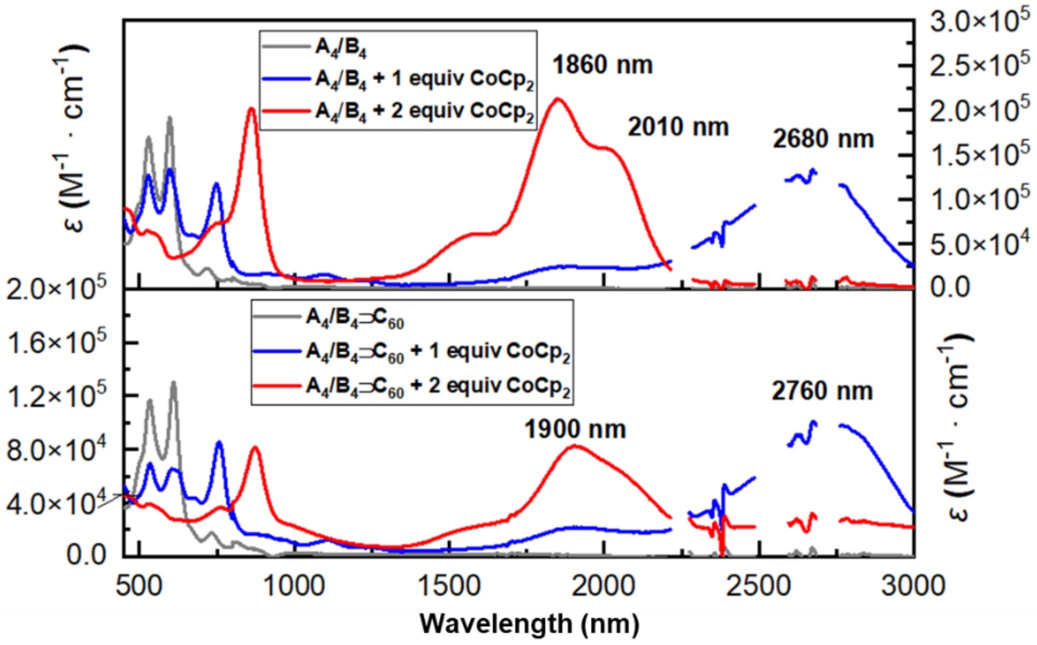

Figure S26. Absorption spectra of **A_4/B_4** and **A_4/$B_4{\supset}C_{60}$** following chemical reduction with $CoCp_2$. (a) **A_4/B_4, $A_4^{\bullet-}$/$B_4^{\bullet-}$**, and **$A_4^{2-\bullet\bullet}$/$B_4^{2-\bullet\bullet}$**; (b) **A_4/$B_4{\supset}C_{60}$, $A_4^{\bullet-}$/$B_4^{\bullet-}{\supset}C_{60}$**, and **$A_4^{2-\bullet\bullet}$/$B_4^{2-\bullet\bullet}{\supset}C_{60}$.**

All samples were measured at 5.0 × 10^{-6} M in DCM.

All reduced species exhibit intense near- to mid-IR absorptions with high extinction coefficients (ε_{max} > 10^5 $M^{-1}\cdot cm^{-1}$), making **[4]C-NDTI** a promising candidate for IR optoelectronics. Overlay comparison revealing that the characteristic absorption peaks exhibit negligible shifts upon C_{60} inclusion, indicating a minimal perturbation of the electronic structure of $\mathbf{B_4}^{\bullet-}$ and $\mathbf{B_4}^{2-\bullet\bullet}$ by guest encapsulation.

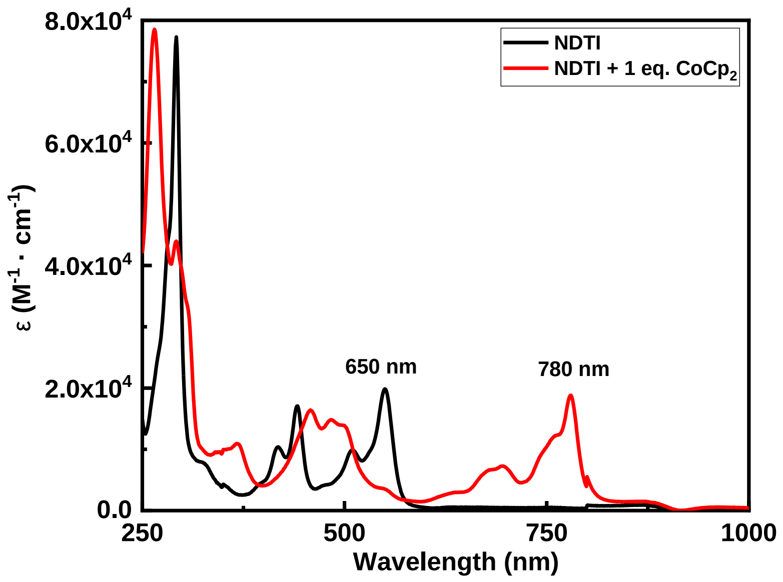

Figure S27. Absorption spectra of NDTI and NDTI$^{\bullet-}$. All samples were measured at 5.0 × 10^{-6} M in DCM.

2) Electrochemical reduction

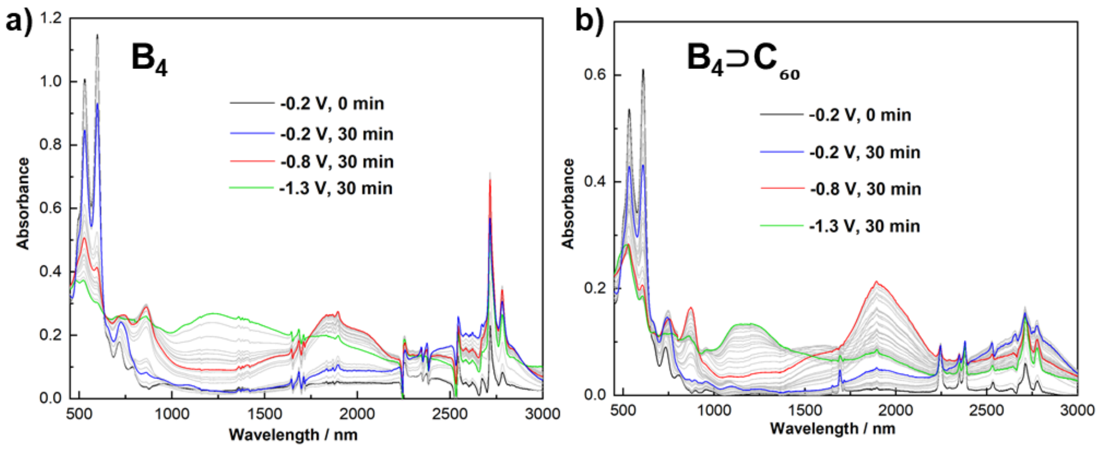

Figure S28. *In Situ* UV-Vis-NIR absorption spectral evolution of (a) $\mathbf{B_4}$ and (b) $\mathbf{B_4 \supset C_{60}}$ in DCM (5.0 × 10^{-5} M) during constant-potential electrolysis at -0.2 V, -0.8 V and -1.3 V. (Supporting electrolyte: 0.1 M Bu_4NPF_6; working and counter electrodes: Pt wires; reference electrode: Ag/AgCl.)

To probe higher reduction states, we also performed *in situ* spectroelectrochemical reduction (monitored by UV-vis-NIR spectroscopy at 6-min intervals) of $\mathbf{B_4}$, by stepping the applied

potential sequentially to more negative values (Fig. S28a). At each potential, the system was held for approximately 30 min to allow the reduction to reach steady state.

Initially, at -0.2 V, the spectral evolution mirrored that of chemical reduction, with the characteristic peaks of the radical anion $\mathbf{B_4}^{\bullet-}$ emerging at 760 and 2680 nm. Then, the potential was stepped to −0.8 V and held for another 30 min. These features diminished, while new absorptions grew across 1300-2650 nm. This signature matched that of the chemically generated diradical dianion $\mathbf{B_4}^{2-\bullet\bullet}$, confirming its formation via electrochemical reduction. Further stepping the potential to -1.3 V for 30 min caused the diradical dianion peaks to gradually disappear, replaced by a broad absorption band spanning 1000-1750 nm, which is absent in samples prepared by chemical reduction with Cp_2Co. This sequential spectral evolution aligns with the voltammetric data, which indicate a third two-electron reduction wave, corresponding to the formation of the tetra-anion $\mathbf{B_4}^{4-}$.

As shown in Fig. S28b, inclusion of C_{60} had little effect on the spectroelectrochemical reduction profile, indicating the redox states and associated spectral signatures of **[4]C-NDTI** remain largely unchanged upon host-guest complexation.

3) Stability of chemically reduced species

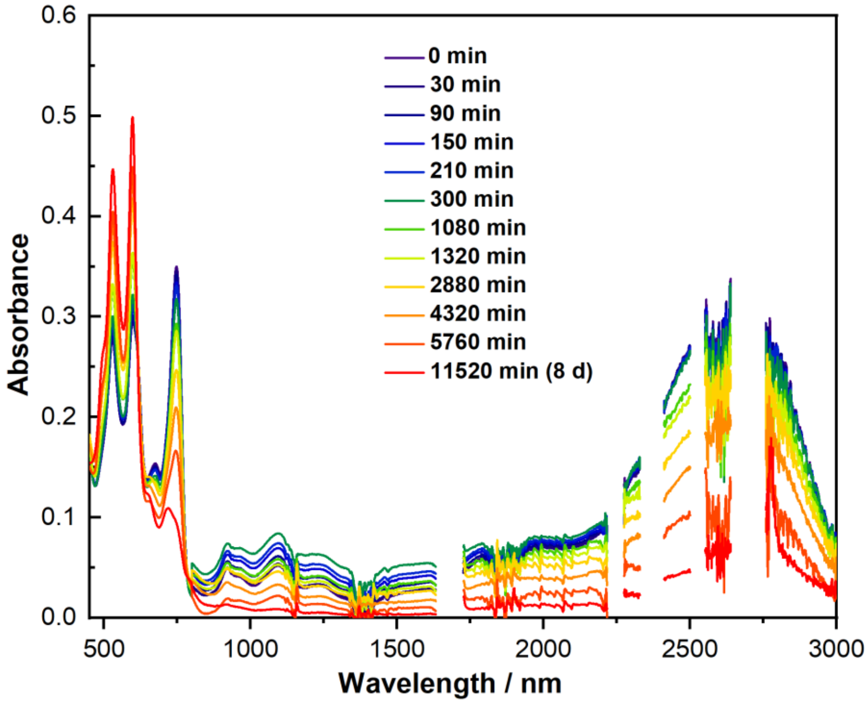

Figure S29. Ambient stability of $\mathbf{A_4}^{\bullet-}$ (5.0×10^{-6} M in DCM) monitored by *in situ* UV-vis-NIR spectroscopy.

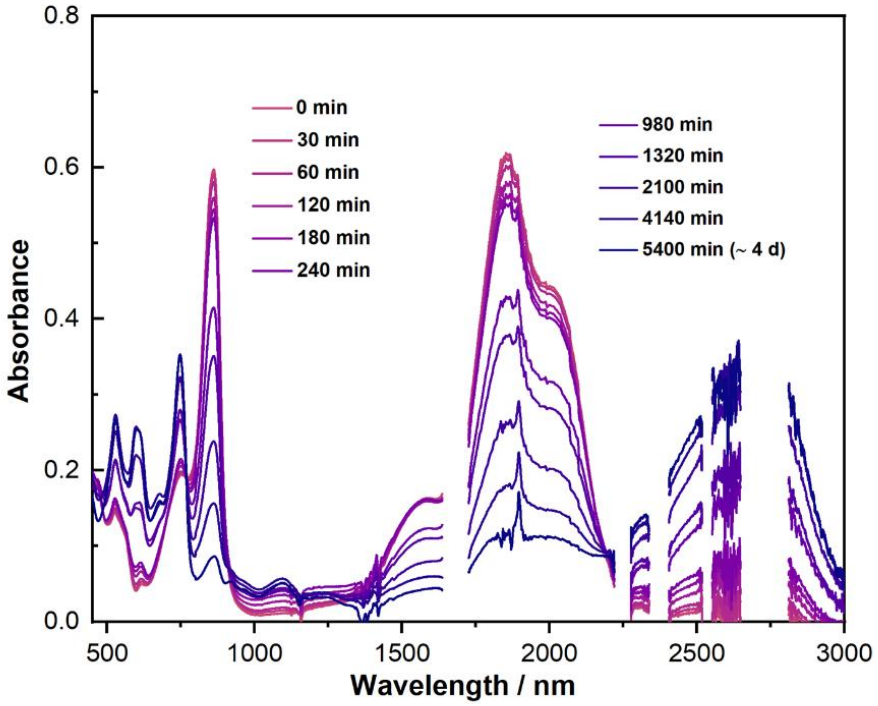

Figure S30. Ambient stability of $\mathbf{A_4}^{2-\bullet\bullet}$ (5.0 × 10^{-6} M in DCM) monitored by *in situ* UV-vis-NIR spectroscopy.

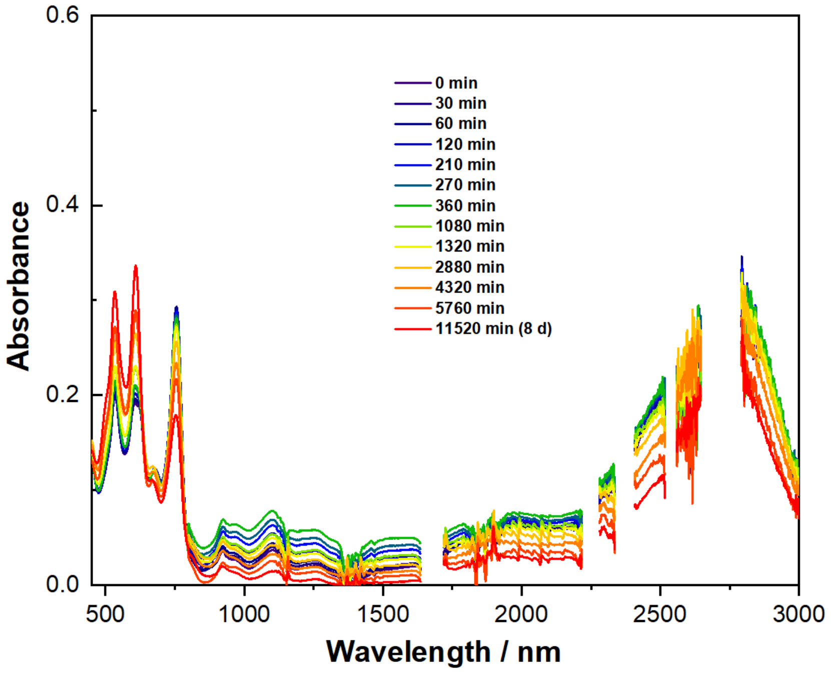

Figure S31. Ambient stability of $\mathbf{A_4}^{\bullet-}$**⊃**$\mathbf{C_{60}}$ (5.0 × 10^{-6} M in DCM) monitored by *in situ* UV-vis-NIR spectroscopy.

Notably, the radical anion signal of the encapsulated complex ($\mathbf{A_4}^{\bullet-}$**⊃**$\mathbf{C_{60}}$) persists for at least 8 days (Fig. S31), indicating enhanced kinetic stability compared to the pristine radical anion (Fig. S29).

V. Electron Paramagnetic Resonance (EPR) Spectroscopy: Spin Delocalization and Diradical Character

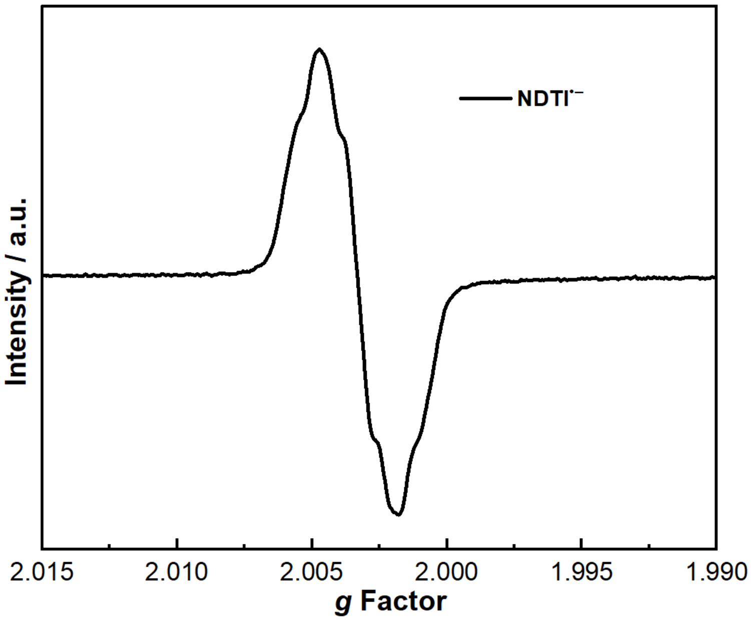

Figure S32. EPR spectra of the radical anion species NDTI•– in DCM at 100 K.

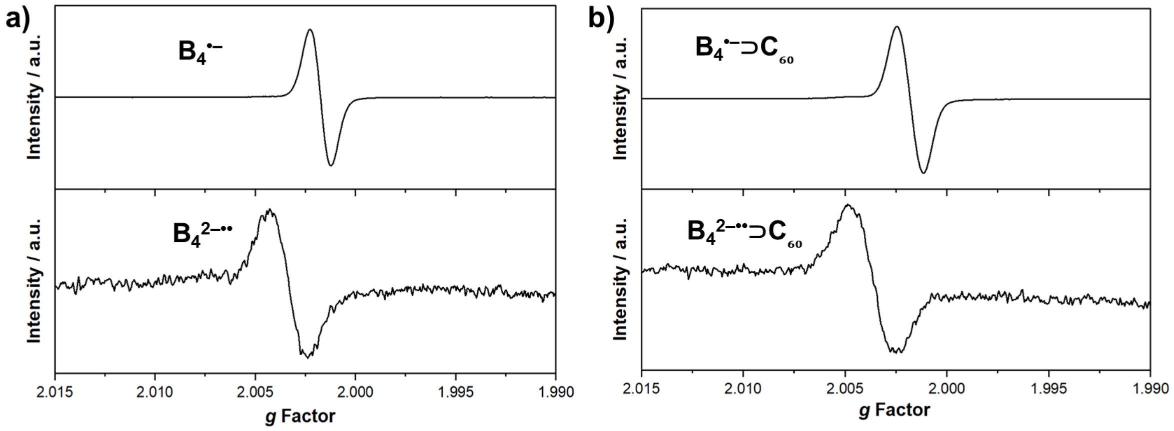

Figure S33. a) EPR spectra of the radical anion species $\mathbf{B_4^{\bullet-}}$ and the diradical dianion $\mathbf{B_4^{2-\bullet\bullet}}$ in DCM at 100 K; (b) EPR spectra of fullerene-encapsulated complexes $\mathbf{B_4^{\bullet-}\supset C_{60}}$ and $\mathbf{B_4^{2-\bullet\bullet}\supset C_{60}}$ in DCM at 100 K for comparison.

The singlet-triplet energy gap (ΔE_{ST}) has been extracted from the variable-temperature EPR data by fitting the temperature-dependent double−integral intensities (I) using the modified Bleaney-Bowers equation for a singlet diradical system[17]:

$$IT \propto \frac{A}{3 + exp(\Delta E/k_B T)}$$

Where $\Delta E = E_{Triplet}$-E_{OSS}, k_B = 0.001987 kcal·mol^{-1}·K^{-1}, and IT is the product of double-integral intensity and absolute temperature. The fitting was performed on the normalized IT values

(divided by the maximum at 140 K). $\Delta E = E_{Triplet}\text{-}E_{OSS} = 0.68$ kcal·mol^{-1}

The value indicates that the singlet state is the ground state and lies 0.68 kcal·mol^{-1} below the triplet state. The fit exhibits excellent agreement ($R^2 > 0.99$), as shown in the figure below. This small singlet-triplet gap confirms the significant open-shell singlet diradical character of the compound, and the thermally accessible triplet state is responsible for the temperature-dependent EPR signal enhancement. The extracted ΔE is close to the theoretical calculations ($\Delta E_{\text{T-S}}$ = 1.70 kcal·mol^{-1} for the model compound).

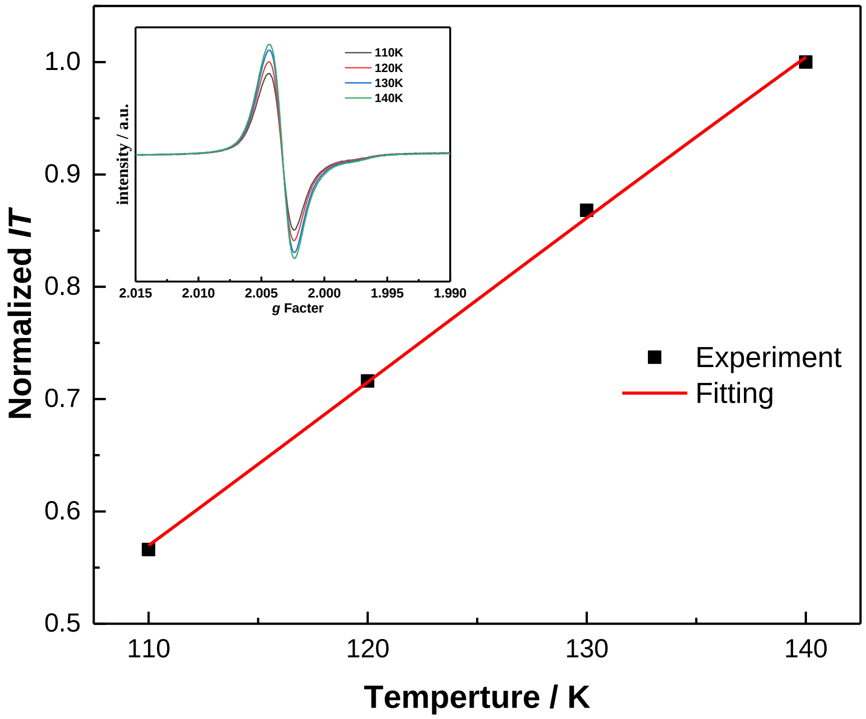

Figure S34. Experimental (black dots) and Bleaney−Bowers fit (red line) of *IT*-T plots based on the variable-temperature EPR spectra (inset) of $\mathbf{B_4}^{2-\bullet\bullet}$ in DCM.

VI. Chiroptical Properties: CD and MCD Spectra

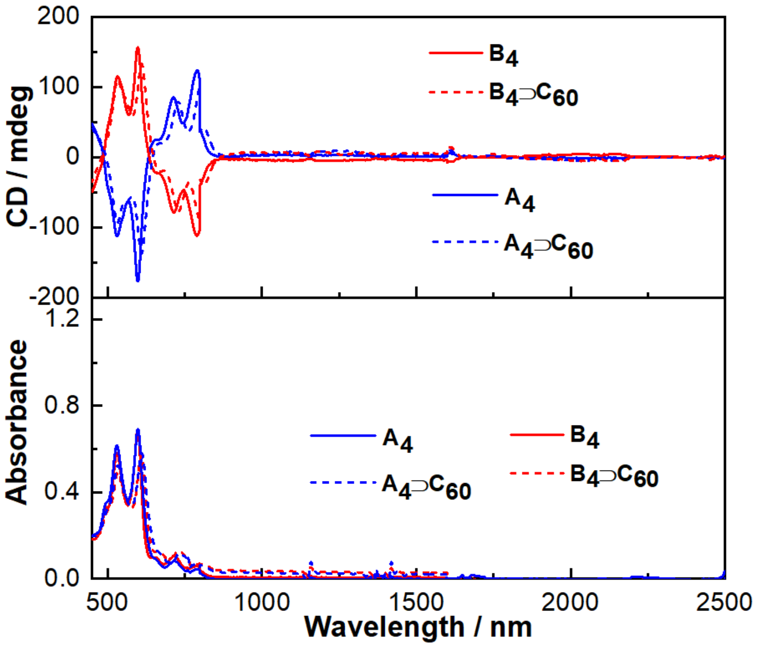

Figure S35. Electronic absorption and CD spectra of **A_4**, **B_4**, **$A_4⊃C_{60}$** and **$B_4⊃C_{60}$** (5.0 μM in DCM).

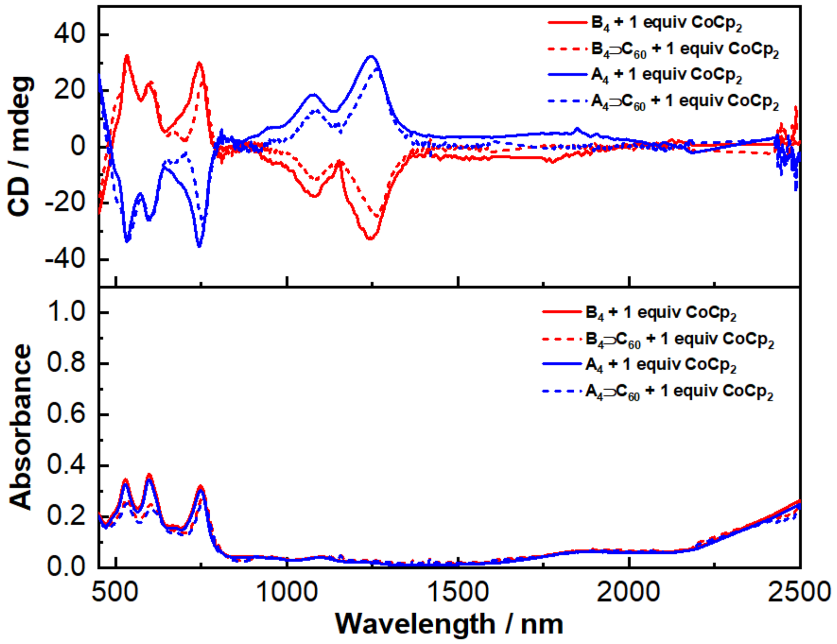

Figure S36. Electronic absorption and CD spectra of **$A_4^{\bullet-}$**, **$B_4^{\bullet-}$**, **$A_4^{\bullet-}⊃C_{60}$** and **$B_4^{\bullet-}⊃C_{60}$** (5.0 μM in DCM).

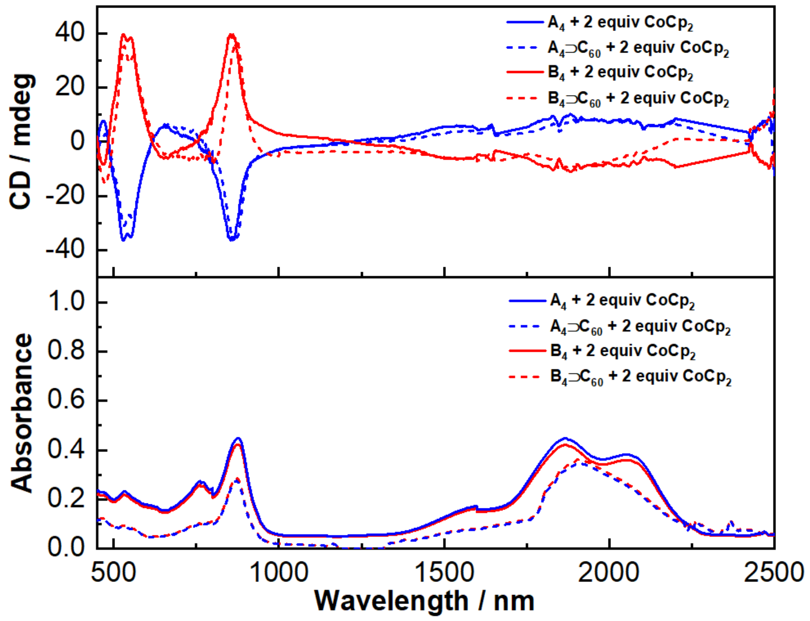

Figure S37. Electronic absorption and CD spectra of $\mathbf{A_4}^{2-\bullet\bullet}$, $\mathbf{B_4}^{2-\bullet\bullet}$, $\mathbf{A_4}^{2-\bullet\bullet}\mathbf{\supset C_{60}}$ and $\mathbf{B_4}^{2-\bullet\bullet}\mathbf{\supset C_{60}}$ (5.0 μM in DCM).

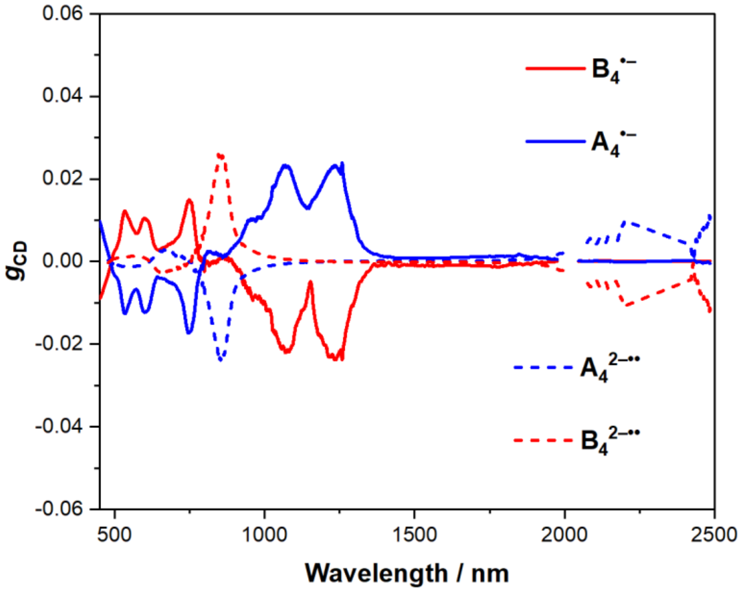

Figure S38. The g_{CD} for $\mathbf{A_4}^{\bullet-}$, $\mathbf{B_4}^{\bullet-}$, $\mathbf{A_4}^{2-\bullet\bullet}$, and $\mathbf{B_4}^{2-\bullet\bullet}$. Spectral regions dominated by solvent absorption (~2200) were excluded from the g_{CD} calculations to ensure accuracy; the resulting gaps in the curves reflect this necessary data processing.

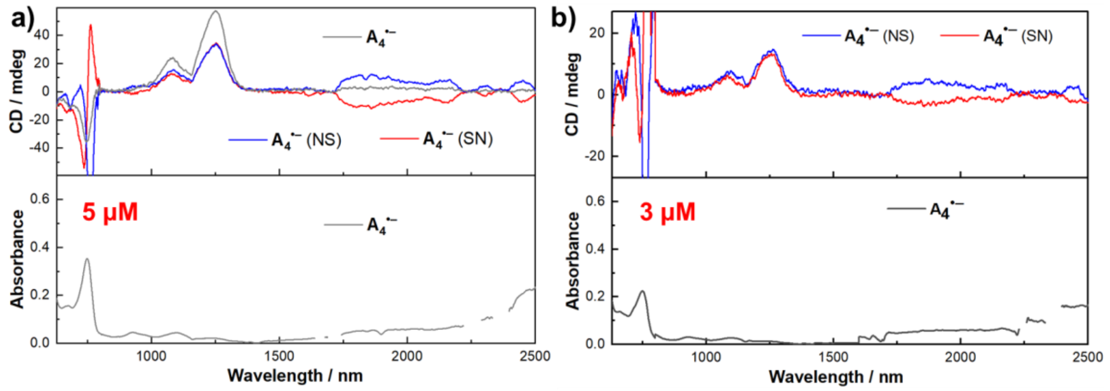

Figure S39. CD spectra of $\mathbf{A_4}^{\bullet-}$ (*in situ* formed from $\mathbf{A_4}$ and 1 equiv of Cp_2Co in DCM) under a 1.6 T field, with its electronic absorption spectrum shown for reference. Samples were prepared from $\mathbf{A_4}$ and Cp_2Co at concentrations of (a) 5.0 μM and (b) 3.0 μM.

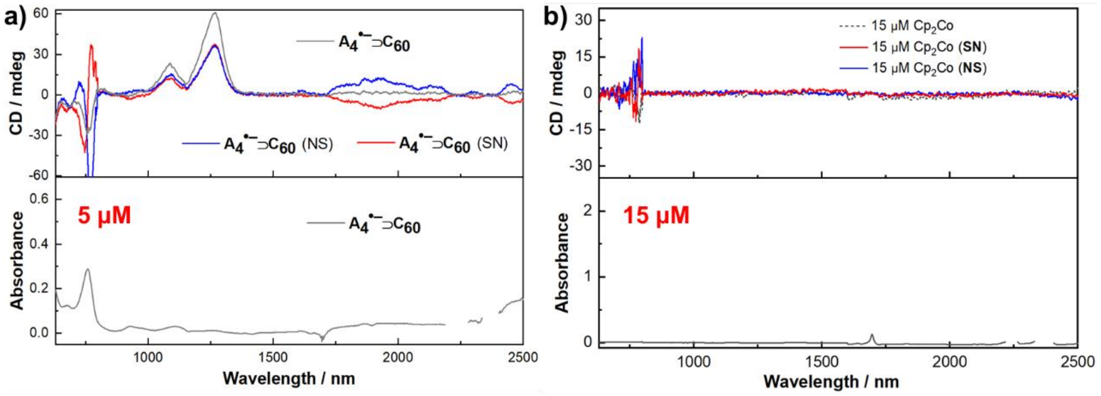

Figure S40. (a) CD spectra of $\mathbf{A_4}^{\bullet-}\supset\mathbf{C_{60}}$ (5.0 μM in DCM) under a 1.6 T field, with its electronic absorption spectrum shown for reference; (b) CD spectra of the Cp_2Co (15.0 μM in DCM) under the same field, measured to exclude its contribution to the signals in Figs. S25 and S26a.

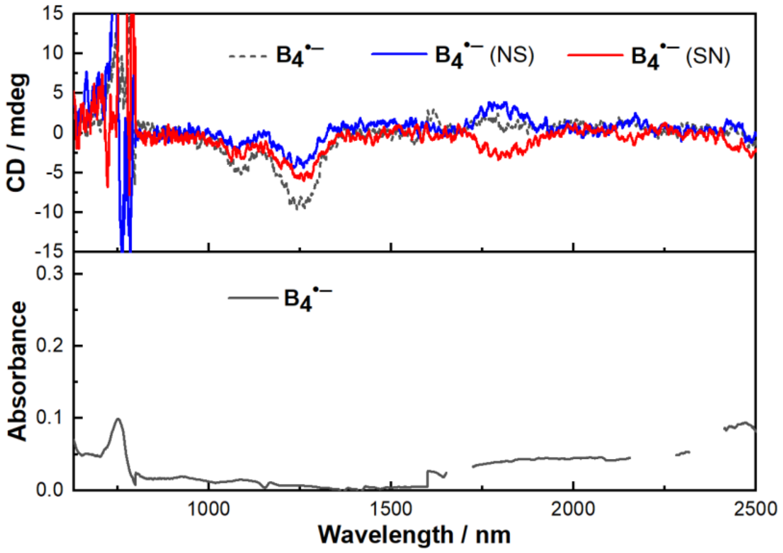

Figure S41. CD spectra of $\mathbf{B_4}^{\bullet-}$ (1.4 μM) under a 1.6 T field, with its electronic absorption spectrum shown for reference;

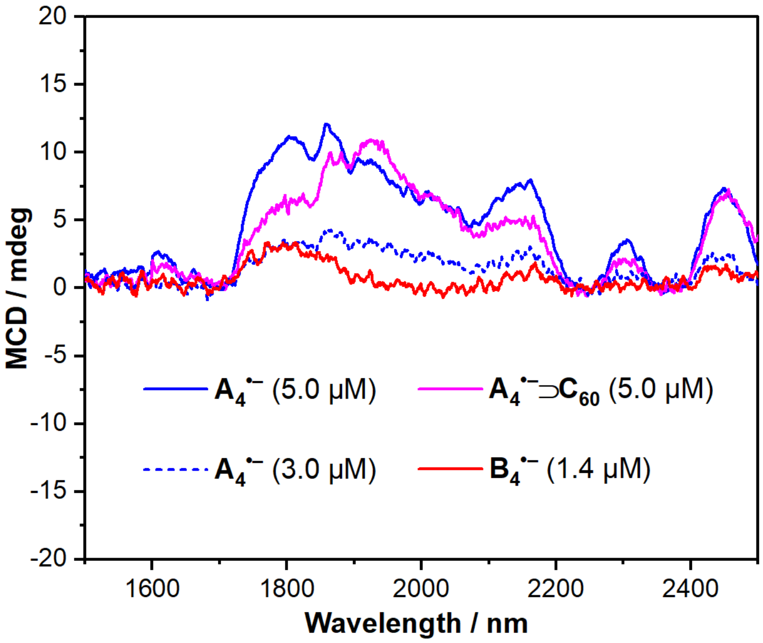

Figure S42. Comparison of the MCD spectra of $\mathbf{A_4^{•–}}$ (5.0 μM), $\mathbf{A_4^{•–}⊃C_{60}}$ (5.0 μM), $\mathbf{A_4^{•–}}$ (3.0 μM), and $\mathbf{B_4^{•–}}$ (1.4 μM) recorded under a 1.6 T field.

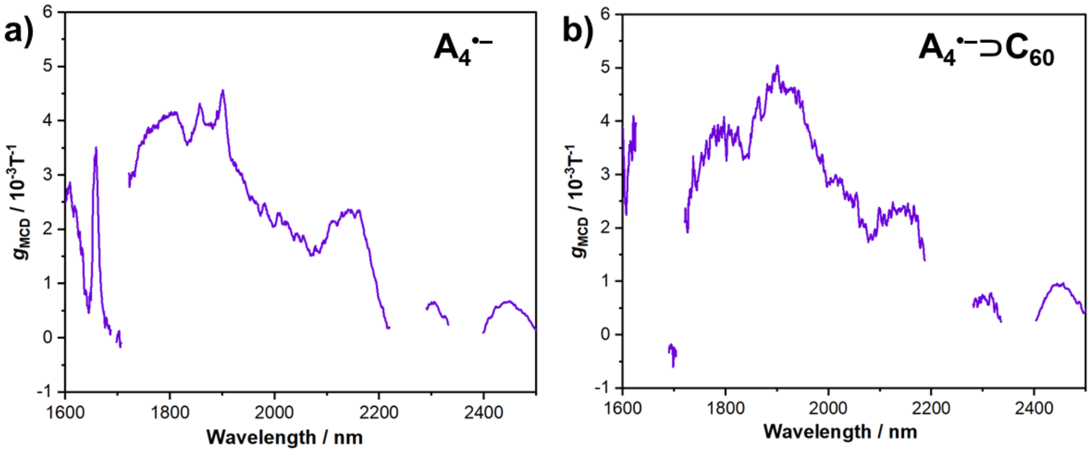

Figure S43. The g_{MCD} for $\mathbf{A_4^{•–}}$ (a) and $\mathbf{A_4^{•–}⊃C_{60}}$ (b) under a 1.6 T field. Spectral regions dominated by solvent absorption (~2200, 2500, and 2700 nm) were excluded from the g_{MCD} calculations to ensure accuracy; the resulting gaps in the curves reflect this necessary data processing.

To elucidate the magneto-optical properties of the radical anions, we conducted a series of control experiments and a more detailed analysis of the MCD signals in the 1700-2500 nm region.

i) Control Experiments

a) To confirm that the observed MCD signals originate solely from the **[4]C-NDTI** radical anions and not from the reductant, we measured the MCD spectrum of $CoCp_2$ under identical conditions (15.0 μM in DCM, 1.6 T). As shown in Fig. S40b, $CoCp_2$ exhibits no detectable MCD signal in the 1600-2500 nm range, ruling out any contribution from the reductant to the spectra shown in Figs. 8 and S39-S41.

b) We compared the MCD spectra of the two enantiomers, $\mathbf{A_4^{•–}}$ and $\mathbf{B_4^{•–}}$, to assess the influence of molecular chirality on the signal orientation. As shown in Fig. S39 and S40, the MCD signals of

$\mathbf{A_4}^{\bullet-}$ and $\mathbf{B_4}^{\bullet-}$ are essentially identical in both sign and shape. This is expected because MCD arises from magnetic field-induced effects, such as state mixing ($\mathcal{B}_0$) or Zeeman splitting of the spin-degenerate ground state ($\mathcal{C}_0$), which are inherently insensitive to the handedness of the molecule. In contrast, the CD signals of the two enantiomers are mirror images (Fig. S36), confirming that the chirality of the macrocycle is preserved in the radical anion state. The lower signal-to-noise ratio in the $\mathbf{B_4}^{\bullet-}$ spectrum is due to its lower concentration (1.4 μM vs. 5.0 μM for $\mathbf{A_4}^{\bullet-}$).

c) We examined the effect of concentration. Fig. S39 compares the MCD spectra of $\mathbf{A_4}^{\bullet-}$ at 5.0 μM and 3.0 μM. The signal intensity scales approximately with concentration while the lineshape remains unchanged, confirming that the signals are intrinsic to $\mathbf{A_4}^{\bullet-}$ and not artifacts of aggregation.

ii) Spectral Lineshape

The MCD spectra of $\mathbf{A_4}^{\bullet-}$ and $\mathbf{B_4}^{\bullet-}$ exhibit unidirectional Gaussian-shaped bands with multiple overlapping components (Fig. S42). This lineshape is characteristic of Faraday terms arising from either field-induced state mixing ($\mathcal{B}_0$) or the spin-degenerate ground state ($\mathcal{C}_0$) of the radical anion. The absence of derivative-shaped ($\mathcal{A}_1$) terms indicates that the excited states involved are non-degenerate, consistent with a minor lifting of degeneracy in the radical anion due to Jahn-Teller distortion or other symmetry-lowering perturbations. This behavior aligns with Michl's perimeter model for perturbed aromatic π-systems.

iii) Dissymmetry Factors

The MCD dissymmetry factors, $g_{\mathrm{MCD}} = \Delta\varepsilon_{\mathrm{MCD}}/\varepsilon$ = MCD/(32980×A×1.6), were calculated for $\mathbf{A_4}^{\bullet-}$ and $\mathbf{A_4}^{\bullet-}\supset\mathbf{C_{60}}$ (Fig. S43). The maximum g_{MCD} values reach 4.6×10^{-3} T^{-1} for $\mathbf{A_4}^{\bullet-}$ and 5.0×10^{-4} T^{-1} for $\mathbf{A_4}^{\bullet-}\supset\mathbf{C_{60}}$ at around 1900 nm. The reduction in g_{MCD} upon C_{60} encapsulation may reflect a slight perturbation of the electronic structure or a dilution effect due to the increased molecular weight. Nevertheless, the magnitude of these *g*-factors signifies a substantial magneto-optical response in the infrared regime, which is notable for organic radicals that typically exhibit weak spin-orbit coupling.

VII. Cartesian Coordinates

$\mathbf{A_4}$ (singlet)
M06-2X(D3)//6-31+G(d)
E = -7893.436500 a.u.

O	3.25793700	6.23057100	3.63383800
N	4.93410700	4.68641700	3.53716600
O	3.01509400	6.45192900	-3.44366900
N	4.68641700	4.93410700	-3.53716600
C	5.04129900	4.64214400	4.99668500
H	6.08997200	4.74055900	5.27901100
H	4.44950100	5.45958800	5.40084300
O	6.45192900	3.01509400	3.44366900
O	6.23057100	3.25793700	-3.63383800
C	4.00239100	5.54075300	2.95901800
C	3.95824900	5.57913100	1.48121700
C	3.82204900	5.66113400	-1.35049100
C	3.78168600	5.72963500	-2.83303100
C	4.84683800	4.78705200	0.71156400
C	4.78705200	4.84683800	-0.71156400
C	5.72963500	3.78168600	2.83303100
C	5.66113400	3.82204900	1.35049100
C	5.57913100	3.95824900	-1.48121700

C	5.54075300	4.00239100	-2.95901800
C	4.64214400	5.04129900	-4.99668500
H	5.45958800	4.44950100	-5.40084300
H	3.68420500	4.66849400	-5.36625500
H	4.74055900	6.08997200	-5.27901100
H	4.66849400	3.68420500	5.36625500
C	2.92937000	6.24910900	0.84425500
C	2.83240400	6.28472400	-0.58840200
C	6.28472400	2.83240400	0.58840200
C	6.24910900	2.92937000	-0.84425500
C	1.55842700	6.79550100	-1.01539800
C	0.72723900	7.06004900	0.03335500
S	1.51778200	6.93191400	1.60698700
H	1.25334500	6.80007600	-2.05321700
C	6.79550100	1.55842700	1.01539800
C	7.06004900	0.72723900	-0.03335500
S	6.93191400	1.51778200	-1.60698700
H	6.80007600	1.25334500	2.05321700
O	6.23057100	-3.25793700	3.63383800
N	4.68641700	-4.93410700	3.53716600
O	6.45192900	-3.01509400	-3.44366900
N	4.93410700	-4.68641700	-3.53716600
C	4.64214400	-5.04129900	4.99668500
H	4.74055900	-6.08997200	5.27901100
H	5.45958800	-4.44950100	5.40084300
O	3.01509400	-6.45192900	3.44366900
O	3.25793700	-6.23057100	-3.63383800
C	5.54075300	-4.00239100	2.95901800
C	5.57913100	-3.95824900	1.48121700
C	5.66113400	-3.82204900	-1.35049100
C	5.72963500	-3.78168600	-2.83303100
C	4.78705200	-4.84683800	0.71156400
C	4.84683800	-4.78705200	-0.71156400
C	3.78168600	-5.72963500	2.83303100
C	3.82204900	-5.66113400	1.35049100
C	3.95824900	-5.57913100	-1.48121700
C	4.00239100	-5.54075300	-2.95901800
C	5.04129900	-4.64214400	-4.99668500
H	4.44950100	-5.45958800	-5.40084300
H	4.66849400	-3.68420500	-5.36625500
H	6.08997200	-4.74055900	-5.27901100
H	3.68420500	-4.66849400	5.36625500
C	6.24910900	-2.92937000	0.84425500
C	6.28472400	-2.83240400	-0.58840200
C	2.83240400	-6.28472400	0.58840200
C	2.92937000	-6.24910900	-0.84425500
C	6.79550100	-1.55842700	-1.01539800
C	7.06004900	-0.72723900	0.03335500
S	6.93191400	-1.51778200	1.60698700
H	6.80007600	-1.25334500	-2.05321700
C	1.55842700	-6.79550100	1.01539800
C	0.72723900	-7.06004900	-0.03335500
S	1.51778200	-6.93191400	-1.60698700
H	1.25334500	-6.80007600	2.05321700
O	-3.25793700	-6.23057100	3.63383800
N	-4.93410700	-4.68641700	3.53716600
O	-3.01509400	-6.45192900	-3.44366900

N	-4.68641700	-4.93410700	-3.53716600
C	-5.04129900	-4.64214400	4.99668500
H	-6.08997200	-4.74055900	5.27901100
H	-4.44950100	-5.45958800	5.40084300
O	-6.45192900	-3.01509400	3.44366900
O	-6.23057100	-3.25793700	-3.63383800
C	-4.00239100	-5.54075300	2.95901800
C	-3.95824900	-5.57913100	1.48121700
C	-3.82204900	-5.66113400	-1.35049100
C	-3.78168600	-5.72963500	-2.83303100
C	-4.84683800	-4.78705200	0.71156400
C	-4.78705200	-4.84683800	-0.71156400
C	-5.72963500	-3.78168600	2.83303100
C	-5.66113400	-3.82204900	1.35049100
C	-5.57913100	-3.95824900	-1.48121700
C	-5.54075300	-4.00239100	-2.95901800
C	-4.64214400	-5.04129900	-4.99668500
H	-5.45958800	-4.44950100	-5.40084300
H	-3.68420500	-4.66849400	-5.36625500
H	-4.74055900	-6.08997200	-5.27901100
H	-4.66849400	-3.68420500	5.36625500
C	-2.92937000	-6.24910900	0.84425500
C	-2.83240400	-6.28472400	-0.58840200
C	-6.28472400	-2.83240400	0.58840200
C	-6.24910900	-2.92937000	-0.84425500
C	-1.55842700	-6.79550100	-1.01539800
C	-0.72723900	-7.06004900	0.03335500
S	-1.51778200	-6.93191400	1.60698700
H	-1.25334500	-6.80007600	-2.05321700
C	-6.79550100	-1.55842700	1.01539800
C	-7.06004900	-0.72723900	-0.03335500
S	-6.93191400	-1.51778200	-1.60698700
H	-6.80007600	-1.25334500	2.05321700
O	-6.23057100	3.25793700	3.63383800
N	-4.68641700	4.93410700	3.53716600
O	-6.45192900	3.01509400	-3.44366900
N	-4.93410700	4.68641700	-3.53716600
C	-4.64214400	5.04129900	4.99668500
H	-4.74055900	6.08997200	5.27901100
H	-5.45958800	4.44950100	5.40084300
O	-3.01509400	6.45192900	3.44366900
O	-3.25793700	6.23057100	-3.63383800
C	-5.54075300	4.00239100	2.95901800
C	-5.57913100	3.95824900	1.48121700
C	-5.66113400	3.82204900	-1.35049100
C	-5.72963500	3.78168600	-2.83303100
C	-4.78705200	4.84683800	0.71156400
C	-4.84683800	4.78705200	-0.71156400
C	-3.78168600	5.72963500	2.83303100
C	-3.82204900	5.66113400	1.35049100
C	-3.95824900	5.57913100	-1.48121700
C	-4.00239100	5.54075300	-2.95901800
C	-5.04129900	4.64214400	-4.99668500
H	-4.44950100	5.45958800	-5.40084300
H	-4.66849400	3.68420500	-5.36625500
H	-6.08997200	4.74055900	-5.27901100
H	-3.68420500	4.66849400	5.36625500

C	-6.24910900	2.92937000	0.84425500
C	-6.28472400	2.83240400	-0.58840200
C	-2.83240400	6.28472400	0.58840200
C	-2.92937000	6.24910900	-0.84425500
C	-6.79550100	1.55842700	-1.01539800
C	-7.06004900	0.72723900	0.03335500
S	-6.93191400	1.51778200	1.60698700
H	-6.80007600	1.25334500	-2.05321700
C	-1.55842700	6.79550100	1.01539800
C	-0.72723900	7.06004900	-0.03335500
S	-1.51778200	6.93191400	-1.60698700
H	-1.25334500	6.80007600	2.05321700

$A_4^{-\bullet}$
(U)M06-2X(D3)//6-31+G(d)
E = -7893.565628 a.u.

O	3.26822900	6.25223500	3.63432900
N	4.93446400	4.69660400	3.52959800
O	3.02784800	6.45646400	-3.44752100
N	4.69660400	4.93446400	-3.52959800
C	5.03812800	4.64865000	4.98691100
H	6.08449400	4.76002100	5.27458100
H	4.43239100	5.45559700	5.39180200
O	6.45646400	3.02784800	3.44752100
O	6.25223500	3.26822900	-3.63432900
C	4.00490800	5.55531400	2.95223900
C	3.95850000	5.58638100	1.48078000
C	3.82643200	5.66231600	-1.35212600
C	3.79002000	5.73274100	-2.82623200
C	4.84248600	4.78198100	0.71396600
C	4.78198100	4.84248600	-0.71396600
C	5.73274100	3.79002000	2.82623200
C	5.66231600	3.82643200	1.35212600
C	5.58638100	3.95850000	-1.48078000
C	5.55531400	4.00490800	-2.95223900
C	4.64865000	5.03812800	-4.98691100
H	5.45559700	4.43239100	-5.39180200
H	3.68338500	4.67961800	-5.35221500
H	4.76002100	6.08449400	-5.27458100
H	4.67961800	3.68338500	5.35221500
C	2.92826600	6.25777100	0.84062700
C	2.82888500	6.29376700	-0.58908700
C	6.29376700	2.82888500	0.58908700
C	6.25777100	2.92826600	-0.84062700
C	1.56434300	6.80356000	-1.01558400
C	0.72183000	7.07125100	0.03295300
S	1.51723500	6.93908500	1.60401400
H	1.26077100	6.80813200	-2.05395900
C	6.80356000	1.56434300	1.01558400
C	7.07125100	0.72183000	-0.03295300
S	6.93908500	1.51723500	-1.60401400
H	6.80813200	1.26077100	2.05395900
O	6.25223500	-3.26822900	3.63432900
N	4.69660400	-4.93446400	3.52959800
O	6.45646400	-3.02784800	-3.44752100
N	4.93446400	-4.69660400	-3.52959800
C	4.64865000	-5.03812800	4.98691100

H	4.76002100	-6.08449400	5.27458100
H	5.45559700	-4.43239100	5.39180200
O	3.02784800	-6.45646400	3.44752100
O	3.26822900	-6.25223500	-3.63432900
C	5.55531400	-4.00490800	2.95223900
C	5.58638100	-3.95850000	1.48078000
C	5.66231600	-3.82643200	-1.35212600
C	5.73274100	-3.79002000	-2.82623200
C	4.78198100	-4.84248600	0.71396600
C	4.84248600	-4.78198100	-0.71396600
C	3.79002000	-5.73274100	2.82623200
C	3.82643200	-5.66231600	1.35212600
C	3.95850000	-5.58638100	-1.48078000
C	4.00490800	-5.55531400	-2.95223900
C	5.03812800	-4.64865000	-4.98691100
H	4.43239100	-5.45559700	-5.39180200
H	4.67961800	-3.68338500	-5.35221500
H	6.08449400	-4.76002100	-5.27458100
H	3.68338500	-4.67961800	5.35221500
C	6.25777100	-2.92826600	0.84062700
C	6.29376700	-2.82888500	-0.58908700
C	2.82888500	-6.29376700	0.58908700
C	2.92826600	-6.25777100	-0.84062700
C	6.80356000	-1.56434300	-1.01558400
C	7.07125100	-0.72183000	0.03295300
S	6.93908500	-1.51723500	1.60401400
H	6.80813200	-1.26077100	-2.05395900
C	1.56434300	-6.80356000	1.01558400
C	0.72183000	-7.07125100	-0.03295300
S	1.51723500	-6.93908500	-1.60401400
H	1.26077100	-6.80813200	2.05395900
O	-3.26822900	-6.25223500	3.63432900
N	-4.93446400	-4.69660400	3.52959800
O	-3.02784800	-6.45646400	-3.44752100
N	-4.69660400	-4.93446400	-3.52959800
C	-5.03812800	-4.64865000	4.98691100
H	-6.08449400	-4.76002100	5.27458100
H	-4.43239100	-5.45559700	5.39180200
O	-6.45646400	-3.02784800	3.44752100
O	-6.25223500	-3.26822900	-3.63432900
C	-4.00490800	-5.55531400	2.95223900
C	-3.95850000	-5.58638100	1.48078000
C	-3.82643200	-5.66231600	-1.35212600
C	-3.79002000	-5.73274100	-2.82623200
C	-4.84248600	-4.78198100	0.71396600
C	-4.78198100	-4.84248600	-0.71396600
C	-5.73274100	-3.79002000	2.82623200
C	-5.66231600	-3.82643200	1.35212600
C	-5.58638100	-3.95850000	-1.48078000
C	-5.55531400	-4.00490800	-2.95223900
C	-4.64865000	-5.03812800	-4.98691100
H	-5.45559700	-4.43239100	-5.39180200
H	-3.68338500	-4.67961800	-5.35221500
H	-4.76002100	-6.08449400	-5.27458100
H	-4.67961800	-3.68338500	5.35221500
C	-2.92826600	-6.25777100	0.84062700
C	-2.82888500	-6.29376700	-0.58908700

C	-6.29376700	-2.82888500	0.58908700
C	-6.25777100	-2.92826600	-0.84062700
C	-1.56434300	-6.80356000	-1.01558400
C	-0.72183000	-7.07125100	0.03295300
S	-1.51723500	-6.93908500	1.60401400
H	-1.26077100	-6.80813200	-2.05395900
C	-6.80356000	-1.56434300	1.01558400
C	-7.07125100	-0.72183000	-0.03295300
S	-6.93908500	-1.51723500	-1.60401400
H	-6.80813200	-1.26077100	2.05395900
O	-6.25223500	3.26822900	3.63432900
N	-4.69660400	4.93446400	3.52959800
O	-6.45646400	3.02784800	-3.44752100
N	-4.93446400	4.69660400	-3.52959800
C	-4.64865000	5.03812800	4.98691100
H	-4.76002100	6.08449400	5.27458100
H	-5.45559700	4.43239100	5.39180200
O	-3.02784800	6.45646400	3.44752100
O	-3.26822900	6.25223500	-3.63432900
C	-5.55531400	4.00490800	2.95223900
C	-5.58638100	3.95850000	1.48078000
C	-5.66231600	3.82643200	-1.35212600
C	-5.73274100	3.79002000	-2.82623200
C	-4.78198100	4.84248600	0.71396600
C	-4.84248600	4.78198100	-0.71396600
C	-3.79002000	5.73274100	2.82623200
C	-3.82643200	5.66231600	1.35212600
C	-3.95850000	5.58638100	-1.48078000
C	-4.00490800	5.55531400	-2.95223900
C	-5.03812800	4.64865000	-4.98691100
H	-4.43239100	5.45559700	-5.39180200
H	-4.67961800	3.68338500	-5.35221500
H	-6.08449400	4.76002100	-5.27458100
H	-3.68338500	4.67961800	5.35221500
C	-6.25777100	2.92826600	0.84062700
C	-6.29376700	2.82888500	-0.58908700
C	-2.82888500	6.29376700	0.58908700
C	-2.92826600	6.25777100	-0.84062700
C	-6.80356000	1.56434300	-1.01558400
C	-7.07125100	0.72183000	0.03295300
S	-6.93908500	1.51723500	1.60401400
H	-6.80813200	1.26077100	-2.05395900
C	-1.56434300	6.80356000	1.01558400
C	-0.72183000	7.07125100	-0.03295300
S	-1.51723500	6.93908500	-1.60401400
H	-1.26077100	6.80813200	2.05395900

$A_4^{2-\bullet\bullet}$ (OSS)
(U)M06-2X(D3)//6-31+G(d)
E = -7893.633665 a.u.

O	0.47211200	-7.07400400	3.64546600
N	-1.76406000	-6.61881500	3.51591500
O	0.80766300	-7.11049500	-3.44101500
N	-1.41626200	-6.69766000	-3.51686000
C	-1.88397200	-6.63595700	4.96960300
H	-2.71895900	-7.27903600	5.25348000
H	-0.94578900	-7.00521400	5.37702100

O	-3.93492300	-5.98349100	3.44015000
O	-3.61711600	-6.09427900	-3.64619400
C	-0.51559300	-6.86143600	2.94583400
C	-0.45528300	-6.85433700	1.48780800
C	-0.29630000	-6.84189500	-1.35033200
C	-0.22350000	-6.89316200	-2.81093900
C	-1.62504300	-6.60978900	0.71839800
C	-1.53688700	-6.63003700	-0.71925300
C	-2.91642200	-6.25405500	2.81007800
C	-2.82781400	-6.23933900	1.34954300
C	-2.69088500	-6.31933000	-1.48860600
C	-2.63996300	-6.35128400	-2.94664900
C	-1.31655900	-6.76480500	-4.97061600
H	-2.32047000	-6.67188400	-5.37814200
H	-0.67990900	-5.95570300	-5.33792100
H	-0.86004700	-7.71433900	-5.25573800
H	-2.08881600	-5.62749400	5.33824300
C	0.78472500	-6.88428600	0.84366500
C	0.89373300	-6.85795700	-0.57317700
C	-3.89658700	-5.71531200	0.57268600
C	-3.81090900	-5.78671600	-0.84420100
C	2.23214200	-6.61708400	-0.99523500
C	3.09877900	-6.41438500	0.05500500
S	2.33727900	-6.71327100	1.61675200
H	2.48996500	-6.45693100	-2.03432900
C	-4.98185300	-4.89610100	0.99529900
C	-5.66327300	-4.32291600	-0.05459600
S	-5.11882200	-4.93247100	-1.61674100
H	-5.13963000	-4.63758200	2.03459300
O	-7.09034300	-0.49543800	3.62495500
N	-6.60781800	1.73216300	3.52730300
O	-7.12509100	-0.77371200	-3.46082600
N	-6.67646400	1.43957400	-3.52688300
C	-6.62261600	1.84084500	4.98366200
H	-7.30739300	2.63783600	5.27936900
H	-6.94131100	0.88257200	5.38650800
O	-6.00835700	3.90944900	3.46117600
O	-6.10047100	3.64488900	-3.62437400
C	-6.86131100	0.49430700	2.94470900
C	-6.84907800	0.44243200	1.47295700
C	-6.84345200	0.30329800	-1.35716200
C	-6.89168500	0.24534000	-2.82893200
C	-6.62495400	1.62272500	0.71290300
C	-6.64328900	1.54506500	-0.71245100
C	-6.26014200	2.89474000	2.82934500
C	-6.24261600	2.82100000	1.35761800
C	-6.30972300	2.69924100	-1.47246100
C	-6.34358400	2.65844800	-2.94420900
C	-6.73776200	1.34922000	-4.98327600
H	-6.58936100	2.34819300	-5.38598100
H	-5.96020700	0.67161600	-5.34319000
H	-7.70831600	0.94708700	-5.27966200
H	-5.62317900	2.09475500	5.34413400
C	-6.87964500	-0.78386700	0.82924600
C	-6.85564100	-0.88354500	-0.60316700
C	-5.71685900	3.88517500	0.60372700
C	-5.78298200	3.80701400	-0.82867000

C	-6.62998800	-2.22443200	-1.03522000
C	-6.40571100	-3.08893200	0.00900300
S	-6.72136500	-2.34794000	1.58343200
H	-6.47008900	-2.48185500	-2.07349700
C	-4.90955700	4.97928500	1.03593400
C	-4.31875300	5.64908800	-0.00826000
S	-4.93518700	5.13096300	-1.58268300
H	-4.65068900	5.13663900	2.07425300
O	-0.47211200	7.07400400	3.64546600
N	1.76406000	6.61881500	3.51591500
O	-0.80766300	7.11049500	-3.44101500
N	1.41626200	6.69766000	-3.51686000
C	1.88397200	6.63595700	4.96960300
H	2.71895900	7.27903600	5.25348000
H	0.94578900	7.00521400	5.37702100
O	3.93492300	5.98349100	3.44015000
O	3.61711600	6.09427900	-3.64619400
C	0.51559300	6.86143600	2.94583400
C	0.45528300	6.85433700	1.48780800
C	0.29630000	6.84189500	-1.35033200
C	0.22350000	6.89316200	-2.81093900
C	1.62504300	6.60978900	0.71839800
C	1.53688700	6.63003700	-0.71925300
C	2.91642200	6.25405500	2.81007800
C	2.82781400	6.23933900	1.34954300
C	2.69088500	6.31933000	-1.48860600
C	2.63996300	6.35128400	-2.94664900
C	1.31655900	6.76480500	-4.97061600
H	2.32047000	6.67188400	-5.37814200
H	0.67990900	5.95570300	-5.33792100
H	0.86004700	7.71433900	-5.25573800
H	2.08881600	5.62749400	5.33824300
C	-0.78472500	6.88428600	0.84366500
C	-0.89373300	6.85795700	-0.57317700
C	3.89658700	5.71531200	0.57268600
C	3.81090900	5.78671600	-0.84420100
C	-2.23214200	6.61708400	-0.99523500
C	-3.09877900	6.41438500	0.05500500
S	-2.33727900	6.71327100	1.61675200
H	-2.48996500	6.45693100	-2.03432900
C	4.98185300	4.89610100	0.99529900
C	5.66327300	4.32291600	-0.05459600
S	5.11882200	4.93247100	-1.61674100
H	5.13963000	4.63758200	2.03459300
O	7.09034300	0.49543800	3.62495500
N	6.60781800	-1.73216300	3.52730300
O	7.12509100	0.77371200	-3.46082600
N	6.67646400	-1.43957400	-3.52688300
C	6.62261600	-1.84084500	4.98366200
H	7.30739200	-2.63783600	5.27936900
H	6.94131100	-0.88257100	5.38650800
O	6.00835700	-3.90944900	3.46117600
O	6.10047100	-3.64488900	-3.62437400
C	6.86131100	-0.49430700	2.94470900
C	6.84907800	-0.44243200	1.47295700
C	6.84345200	-0.30329800	-1.35716200
C	6.89168500	-0.24534000	-2.82893200

C	6.62495400	-1.62272500	0.71290300
C	6.64328900	-1.54506500	-0.71245100
C	6.26014200	-2.89474000	2.82934500
C	6.24261600	-2.82100000	1.35761800
C	6.30972300	-2.69924100	-1.47246100
C	6.34358400	-2.65844800	-2.94420900
C	6.73776200	-1.34922000	-4.98327600
H	6.58936100	-2.34819300	-5.38598100
H	5.96020700	-0.67161600	-5.34319000
H	7.70831600	-0.94708700	-5.27966200
H	5.62317900	-2.09475500	5.34413400
C	6.87964500	0.78386700	0.82924600
C	6.85564100	0.88354500	-0.60316700
C	5.71685900	-3.88517500	0.60372700
C	5.78298200	-3.80701400	-0.82867000
C	6.62998800	2.22443200	-1.03522000
C	6.40571100	3.08893200	0.00900300
S	6.72136500	2.34794000	1.58343200
H	6.47008900	2.48185500	-2.07349700
C	4.90955700	-4.97928500	1.03593400
C	4.31875300	-5.64908800	-0.00826000
S	4.93518700	-5.13096300	-1.58268300
H	4.65068900	-5.13663900	2.07425300

$A_4^{2-••}$ (triplet)
(U)M06-2X(D3)//6-31+G(d)
E = -7893.630993 a.u.

O	-4.49492200	-5.50682500	-3.62379300
N	-2.39969800	-6.40261300	-3.53002400
O	-4.74357900	-5.38680200	3.46144600
N	-2.67466200	-6.29103600	3.52678000
C	-2.32244900	-6.47764300	-4.98681300
H	-2.04502500	-7.49136100	-5.28071100
H	-3.29638100	-6.20651500	-5.38692900
O	-0.27684300	-7.17195000	-3.46492000
O	-0.54384300	-7.09911500	3.62010400
C	-3.55475200	-5.89265000	-2.94579300
C	-3.58964800	-5.85304600	-1.47223100
C	-3.70113400	-5.77124100	1.35640100
C	-3.77566900	-5.77977000	2.83003700
C	-2.49927000	-6.35658500	-0.71304800
C	-2.57152100	-6.32810400	0.70971800
C	-1.24929400	-6.79057800	-2.83354100
C	-1.29769900	-6.73363700	-1.35979100
C	-1.43582400	-6.71892100	1.46881500
C	-1.48868700	-6.72431800	2.94248500
C	-2.78304200	-6.29070300	4.98356000
H	-1.87637100	-6.73606600	5.38562900
H	-2.89902200	-5.26586800	5.34308200
H	-3.66481500	-6.86151600	5.28062600
H	-1.55516800	-5.79076400	-5.35154100
C	-4.60715100	-5.16975600	-0.82827000
C	-4.67347900	-5.09493600	0.60526600
C	-0.12782100	-6.91695000	-0.60854100
C	-0.22805700	-6.92769400	0.82494500
C	-5.63946700	-4.13378900	1.03970900
C	-6.21156100	-3.45034700	-0.00226000

S	-5.79218200	-4.13401000	-1.57900100
H	-5.75592400	-3.85426600	2.07790900
C	1.23484000	-6.88664900	-1.04249500
C	2.11996100	-6.78948600	0.00011200
S	1.34415000	-6.99991200	1.57591300
H	1.51235300	-6.76109000	-2.08014300
O	5.51759600	-4.46842100	-3.64658300
N	6.42919800	-2.37541100	-3.51252600
O	5.33685700	-4.76949600	3.43901700
N	6.28339100	-2.71438600	3.51675000
C	6.51389600	-2.28519600	-4.96571000
H	7.52675200	-1.99359600	-5.25034700
H	6.25499500	-3.25875500	-5.37525100
O	7.16406300	-0.23564400	-3.43505400
O	7.05838900	-0.56735800	3.65115800
C	5.91070300	-3.53829300	-2.94418200
C	5.86786800	-3.58443900	-1.48844800
C	5.75874700	-3.70958900	1.35074800
C	5.75607300	-3.80075000	2.80957800
C	6.33758000	-2.48814500	-0.71729000
C	6.30148500	-2.57211500	0.72162700
C	6.79605700	-1.22485600	-2.80539300
C	6.73024100	-1.29067000	-1.34678500
C	6.71415500	-1.45302500	1.49273500
C	6.70755300	-1.51391700	2.94833300
C	6.27932200	-2.83556400	4.97014200
H	6.78993700	-1.96658400	5.37848200
H	5.25073300	-2.88000000	5.33784700
H	6.78307400	-3.76116500	5.25434700
H	5.82375400	-1.52053500	-5.33127300
C	5.17584400	-4.61789100	-0.84411500
C	5.08757700	-4.69031100	0.56870700
C	6.91774200	-0.11660800	-0.56566700
C	6.92667000	-0.22809200	0.84725800
C	4.11202800	-5.64227800	0.98923100
C	3.45387100	-6.23591400	-0.06163100
S	4.13955400	-5.78546800	-1.62029400
H	3.82914500	-5.75713100	2.02793100
C	6.86841500	1.24496600	-0.98807700
C	6.79605700	2.12997900	0.06147500
S	6.97709300	1.33264100	1.62184300
H	6.74694100	1.52169100	-2.02771900
O	4.49492200	5.50682500	-3.62379300
N	2.39969800	6.40261300	-3.53002400
O	4.74357900	5.38680200	3.46144600
N	2.67466200	6.29103600	3.52678000
C	2.32244900	6.47764300	-4.98681300
H	2.04502500	7.49136100	-5.28071100
H	3.29638100	6.20651500	-5.38692900
O	0.27684300	7.17195000	-3.46492000
O	0.54384300	7.09911500	3.62010400
C	3.55475200	5.89265000	-2.94579300
C	3.58964800	5.85304600	-1.47223100
C	3.70113400	5.77124100	1.35640100
C	3.77566900	5.77977000	2.83003700
C	2.49927000	6.35658500	-0.71304800
C	2.57152100	6.32810400	0.70971800

C	1.24929400	6.79057800	-2.83354100
C	1.29769900	6.73363700	-1.35979100
C	1.43582400	6.71892100	1.46881500
C	1.48868700	6.72431800	2.94248500
C	2.78304200	6.29070300	4.98356000
H	1.87637100	6.73606600	5.38562900
H	2.89902200	5.26586800	5.34308200
H	3.66481500	6.86151600	5.28062600
H	1.55516800	5.79076400	-5.35154100
C	4.60715100	5.16975600	-0.82827000
C	4.67347900	5.09493600	0.60526600
C	0.12782100	6.91695000	-0.60854100
C	0.22805700	6.92769400	0.82494500
C	5.63946700	4.13378900	1.03970900
C	6.21156100	3.45034700	-0.00226000
S	5.79218200	4.13401000	-1.57900100
H	5.75592400	3.85426600	2.07790900
C	-1.23484000	6.88664900	-1.04249500
C	-2.11996100	6.78948600	0.00011200
S	-1.34415000	6.99991200	1.57591300
H	-1.51235300	6.76109000	-2.08014300
O	-5.51759600	4.46842100	-3.64658300
N	-6.42919800	2.37541100	-3.51252600
O	-5.33685700	4.76949600	3.43901700
N	-6.28339100	2.71438600	3.51675000
C	-6.51389600	2.28519600	-4.96571000
H	-7.52675200	1.99359600	-5.25034700
H	-6.25499500	3.25875500	-5.37525100
O	-7.16406300	0.23564400	-3.43505400
O	-7.05838900	0.56735800	3.65115800
C	-5.91070300	3.53829300	-2.94418200
C	-5.86786800	3.58443900	-1.48844800
C	-5.75874700	3.70958900	1.35074800
C	-5.75607300	3.80075000	2.80957800
C	-6.33758000	2.48814500	-0.71729000
C	-6.30148500	2.57211500	0.72162700
C	-6.79605700	1.22485600	-2.80539300
C	-6.73024100	1.29067000	-1.34678500
C	-6.71415500	1.45302500	1.49273500
C	-6.70755300	1.51391700	2.94833300
C	-6.27932200	2.83556400	4.97014200
H	-6.78993700	1.96658400	5.37848200
H	-5.25073300	2.88000000	5.33784700
H	-6.78307400	3.76116500	5.25434700
H	-5.82375400	1.52053500	-5.33127300
C	-5.17584400	4.61789100	-0.84411500
C	-5.08757700	4.69031100	0.56870700
C	-6.91774200	0.11660800	-0.56566700
C	-6.92667000	0.22809200	0.84725800
C	-4.11202800	5.64227800	0.98923100
C	-3.45387100	6.23591400	-0.06163100
S	-4.13955400	5.78546800	-1.62029400
H	-3.82914500	5.75713100	2.02793100
C	-6.86841500	-1.24496600	-0.98807700
C	-6.79605700	-2.12997900	0.06147500
S	-6.97709300	-1.33264100	1.62184300
H	-6.74694100	-1.52169100	-2.02771900

B_4 (singlet)
M06-2X(D3)//6-31+G(d)
E = -7893.436500 a.u.

O	-3.25793700	6.23057100	3.63383800
N	-4.93410800	4.68641700	3.53716600
O	-3.01509300	6.45193000	-3.44366800
N	-4.68641700	4.93410800	-3.53716600
C	-5.04130000	4.64214400	4.99668500
H	-6.08997300	4.74056000	5.27901100
H	-4.44950100	5.45958800	5.40084400
O	-6.45193000	3.01509300	3.44366800
O	-6.23057100	3.25793700	-3.63383800
C	-4.00239100	5.54075300	2.95901800
C	-3.95824900	5.57913100	1.48121700
C	-3.82204900	5.66113400	-1.35049100
C	-3.78168600	5.72963500	-2.83303100
C	-4.84683700	4.78705200	0.71156400
C	-4.78705200	4.84683700	-0.71156400
C	-5.72963500	3.78168600	2.83303100
C	-5.66113400	3.82204900	1.35049100
C	-5.57913100	3.95824900	-1.48121700
C	-5.54075300	4.00239100	-2.95901800
C	-4.64214400	5.04130000	-4.99668500
H	-5.45958800	4.44950100	-5.40084400
H	-3.68420400	4.66849700	-5.36625500
H	-4.74056000	6.08997300	-5.27901100
H	-4.66849700	3.68420400	5.36625500
C	-2.92937000	6.24910900	0.84425500
C	-2.83240400	6.28472400	-0.58840200
C	-6.28472400	2.83240400	0.58840200
C	-6.24910900	2.92937000	-0.84425500
C	-1.55842700	6.79550200	-1.01539800
C	-0.72723900	7.06004900	0.03335500
S	-1.51778200	6.93191400	1.60698700
H	-1.25334600	6.80007600	-2.05321700
C	-6.79550200	1.55842700	1.01539800
C	-7.06004900	0.72723900	-0.03335500
S	-6.93191400	1.51778200	-1.60698700
H	-6.80007600	1.25334600	2.05321700
O	-6.23057100	-3.25793700	3.63383800
N	-4.68641700	-4.93410800	3.53716600
O	-6.45193000	-3.01509300	-3.44366800
N	-4.93410800	-4.68641700	-3.53716600
C	-4.64214400	-5.04130000	4.99668500
H	-4.74056000	-6.08997300	5.27901100
H	-5.45958800	-4.44950100	5.40084400
O	-3.01509300	-6.45193000	3.44366800
O	-3.25793700	-6.23057100	-3.63383800
C	-5.54075300	-4.00239100	2.95901800
C	-5.57913100	-3.95824900	1.48121700
C	-5.66113400	-3.82204900	-1.35049100
C	-5.72963500	-3.78168600	-2.83303100
C	-4.78705200	-4.84683700	0.71156400
C	-4.84683700	-4.78705200	-0.71156400
C	-3.78168600	-5.72963500	2.83303100
C	-3.82204900	-5.66113400	1.35049100

C	-3.95824900	-5.57913100	-1.48121700
C	-4.00239100	-5.54075300	-2.95901800
C	-5.04130000	-4.64214400	-4.99668500
H	-4.44950100	-5.45958800	-5.40084400
H	-4.66849700	-3.68420400	-5.36625500
H	-6.08997300	-4.74056000	-5.27901100
H	-3.68420400	-4.66849700	5.36625500
C	-6.24910900	-2.92937000	0.84425500
C	-6.28472400	-2.83240400	-0.58840200
C	-2.83240400	-6.28472400	0.58840200
C	-2.92937000	-6.24910900	-0.84425500
C	-6.79550200	-1.55842700	-1.01539800
C	-7.06004900	-0.72723900	0.03335500
S	-6.93191400	-1.51778200	1.60698700
H	-6.80007600	-1.25334600	-2.05321700
C	-1.55842700	-6.79550200	1.01539800
C	-0.72723900	-7.06004900	-0.03335500
S	-1.51778200	-6.93191400	-1.60698700
H	-1.25334600	-6.80007600	2.05321700
O	3.25793700	-6.23057100	3.63383800
N	4.93410800	-4.68641700	3.53716600
O	3.01509300	-6.45193000	-3.44366800
N	4.68641700	-4.93410800	-3.53716600
C	5.04130000	-4.64214400	4.99668500
H	6.08997300	-4.74056000	5.27901100
H	4.44950100	-5.45958800	5.40084400
O	6.45193000	-3.01509300	3.44366800
O	6.23057100	-3.25793700	-3.63383800
C	4.00239100	-5.54075300	2.95901800
C	3.95824900	-5.57913100	1.48121700
C	3.82204900	-5.66113400	-1.35049100
C	3.78168600	-5.72963500	-2.83303100
C	4.84683700	-4.78705200	0.71156400
C	4.78705200	-4.84683700	-0.71156400
C	5.72963500	-3.78168600	2.83303100
C	5.66113400	-3.82204900	1.35049100
C	5.57913100	-3.95824900	-1.48121700
C	5.54075300	-4.00239100	-2.95901800
C	4.64214400	-5.04130000	-4.99668500
H	5.45958800	-4.44950100	-5.40084400
H	3.68420400	-4.66849700	-5.36625500
H	4.74056000	-6.08997300	-5.27901100
H	4.66849700	-3.68420400	5.36625500
C	2.92937000	-6.24910900	0.84425500
C	2.83240400	-6.28472400	-0.58840200
C	6.28472400	-2.83240400	0.58840200
C	6.24910900	-2.92937000	-0.84425500
C	1.55842700	-6.79550200	-1.01539800
C	0.72723900	-7.06004900	0.03335500
S	1.51778200	-6.93191400	1.60698700
H	1.25334600	-6.80007600	-2.05321700
C	6.79550200	-1.55842700	1.01539800
C	7.06004900	-0.72723900	-0.03335500
S	6.93191400	-1.51778200	-1.60698700
H	6.80007600	-1.25334600	2.05321700
O	6.23057100	3.25793700	3.63383800
N	4.68641700	4.93410800	3.53716600

O	6.45193000	3.01509300	-3.44366800
N	4.93410800	4.68641700	-3.53716600
C	4.64214400	5.04130000	4.99668500
H	4.74056000	6.08997300	5.27901100
H	5.45958800	4.44950100	5.40084400
O	3.01509300	6.45193000	3.44366800
O	3.25793700	6.23057100	-3.63383800
C	5.54075300	4.00239100	2.95901800
C	5.57913100	3.95824900	1.48121700
C	5.66113400	3.82204900	-1.35049100
C	5.72963500	3.78168600	-2.83303100
C	4.78705200	4.84683700	0.71156400
C	4.84683700	4.78705200	-0.71156400
C	3.78168600	5.72963500	2.83303100
C	3.82204900	5.66113400	1.35049100
C	3.95824900	5.57913100	-1.48121700
C	4.00239100	5.54075300	-2.95901800
C	5.04130000	4.64214400	-4.99668500
H	4.44950100	5.45958800	-5.40084400
H	4.66849700	3.68420400	-5.36625500
H	6.08997300	4.74056000	-5.27901100
H	3.68420400	4.66849700	5.36625500
C	6.24910900	2.92937000	0.84425500
C	6.28472400	2.83240400	-0.58840200
C	2.83240400	6.28472400	0.58840200
C	2.92937000	6.24910900	-0.84425500
C	6.79550200	1.55842700	-1.01539800
C	7.06004900	0.72723900	0.03335500
S	6.93191400	1.51778200	1.60698700
H	6.80007600	1.25334600	-2.05321700
C	1.55842700	6.79550200	1.01539800
C	0.72723900	7.06004900	-0.03335500
S	1.51778200	6.93191400	-1.60698700
H	1.25334600	6.80007600	2.05321700

$B_4^{-•}$

(U)M06-2X(D3)//6-31+G(d)

E = -7893.565623 a.u.

O	-3.26789800	6.25152000	3.63457000
N	-4.93670200	4.69888000	3.52941000
O	-3.02794900	6.45635200	-3.44793400
N	-4.69888000	4.93670200	-3.52941000
C	-5.04047400	4.65213000	4.98677500
H	-6.08845400	4.75002800	5.27303400
H	-4.44551500	5.46755000	5.39070300
O	-6.45635200	3.02794900	3.44793400
O	-6.25152000	3.26789800	-3.63457000
C	-4.00521700	5.55560400	2.95207100
C	-3.95885800	5.58670600	1.48079000
C	-3.82692700	5.66289500	-1.35223500
C	-3.79105900	5.73378000	-2.82629100
C	-4.84289400	4.78239700	0.71396000
C	-4.78239700	4.84289400	-0.71396000
C	-5.73378000	3.79105900	2.82629100
C	-5.66289500	3.82692700	1.35223500
C	-5.58670600	3.95885800	-1.48079000
C	-5.55560400	4.00521700	-2.95207100

C	-4.65213000	5.04047400	-4.98677500
H	-5.46755000	4.44551500	-5.39070300
H	-3.69255400	4.66922500	-5.35462700
H	-4.75002800	6.08845400	-5.27303400
H	-4.66922500	3.69255400	5.35462700
C	-2.92830700	6.25763900	0.84061200
C	-2.82906900	6.29381600	-0.58910700
C	-6.29381600	2.82906900	0.58910700
C	-6.25763900	2.92830700	-0.84061200
C	-1.56438700	6.80343800	-1.01567700
C	-0.72180900	7.07080800	0.03282500
S	-1.51694900	6.93795700	1.60403400
H	-1.26126900	6.80788700	-2.05418900
C	-6.80343800	1.56438700	1.01567700
C	-7.07080800	0.72180900	-0.03282500
S	-6.93795700	1.51694900	-1.60403400
H	-6.80788700	1.26126900	2.05418900
O	-6.25152000	-3.26789800	3.63457000
N	-4.69888000	-4.93670200	3.52941000
O	-6.45635200	-3.02794900	-3.44793400
N	-4.93670200	-4.69888000	-3.52941000
C	-4.65213000	-5.04047400	4.98677500
H	-4.75002800	-6.08845400	5.27303400
H	-5.46755000	-4.44551500	5.39070300
O	-3.02794900	-6.45635200	3.44793400
O	-3.26789800	-6.25152000	-3.63457000
C	-5.55560400	-4.00521700	2.95207100
C	-5.58670600	-3.95885800	1.48079000
C	-5.66289500	-3.82692700	-1.35223500
C	-5.73378000	-3.79105900	-2.82629100
C	-4.78239700	-4.84289400	0.71396000
C	-4.84289400	-4.78239700	-0.71396000
C	-3.79105900	-5.73378000	2.82629100
C	-3.82692700	-5.66289500	1.35223500
C	-3.95885800	-5.58670600	-1.48079000
C	-4.00521700	-5.55560400	-2.95207100
C	-5.04047400	-4.65213000	-4.98677500
H	-4.44551500	-5.46755000	-5.39070300
H	-4.66922500	-3.69255400	-5.35462700
H	-6.08845400	-4.75002800	-5.27303400
H	-3.69255400	-4.66922500	5.35462700
C	-6.25763900	-2.92830700	0.84061200
C	-6.29381600	-2.82906900	-0.58910700
C	-2.82906900	-6.29381600	0.58910700
C	-2.92830700	-6.25763900	-0.84061200
C	-6.80343800	-1.56438700	-1.01567700
C	-7.07080800	-0.72180900	0.03282500
S	-6.93795700	-1.51694900	1.60403400
H	-6.80788700	-1.26126900	-2.05418900
C	-1.56438700	-6.80343800	1.01567700
C	-0.72180900	-7.07080800	-0.03282500
S	-1.51694900	-6.93795700	-1.60403400
H	-1.26126900	-6.80788700	2.05418900
O	3.26789800	-6.25152000	3.63457000
N	4.93670200	-4.69888000	3.52941000
O	3.02794900	-6.45635200	-3.44793400
N	4.69888000	-4.93670200	-3.52941000

C	5.04047400	-4.65213000	4.98677500
H	6.08845400	-4.75002800	5.27303400
H	4.44551500	-5.46755000	5.39070300
O	6.45635200	-3.02794900	3.44793400
O	6.25152000	-3.26789800	-3.63457000
C	4.00521700	-5.55560400	2.95207100
C	3.95885800	-5.58670600	1.48079000
C	3.82692700	-5.66289500	-1.35223500
C	3.79105900	-5.73378000	-2.82629100
C	4.84289400	-4.78239700	0.71396000
C	4.78239700	-4.84289400	-0.71396000
C	5.73378000	-3.79105900	2.82629100
C	5.66289500	-3.82692700	1.35223500
C	5.58670600	-3.95885800	-1.48079000
C	5.55560400	-4.00521700	-2.95207100
C	4.65213000	-5.04047400	-4.98677500
H	5.46755000	-4.44551500	-5.39070300
H	3.69255400	-4.66922500	-5.35462700
H	4.75002800	-6.08845400	-5.27303400
H	4.66922500	-3.69255400	5.35462700
C	2.92830700	-6.25763900	0.84061200
C	2.82906900	-6.29381600	-0.58910700
C	6.29381600	-2.82906900	0.58910700
C	6.25763900	-2.92830700	-0.84061200
C	1.56438700	-6.80343800	-1.01567700
C	0.72180900	-7.07080800	0.03282500
S	1.51694900	-6.93795700	1.60403400
H	1.26126900	-6.80788700	-2.05418900
C	6.80343800	-1.56438700	1.01567700
C	7.07080800	-0.72180900	-0.03282500
S	6.93795700	-1.51694900	-1.60403400
H	6.80788700	-1.26126900	2.05418900
O	6.25152000	3.26789800	3.63457000
N	4.69888000	4.93670200	3.52941000
O	6.45635200	3.02794900	-3.44793400
N	4.93670200	4.69888000	-3.52941000
C	4.65213000	5.04047400	4.98677500
H	4.75002800	6.08845400	5.27303400
H	5.46755000	4.44551500	5.39070300
O	3.02794900	6.45635200	3.44793400
O	3.26789800	6.25152000	-3.63457000
C	5.55560400	4.00521700	2.95207100
C	5.58670600	3.95885800	1.48079000
C	5.66289500	3.82692700	-1.35223500
C	5.73378000	3.79105900	-2.82629100
C	4.78239700	4.84289400	0.71396000
C	4.84289400	4.78239700	-0.71396000
C	3.79105900	5.73378000	2.82629100
C	3.82692700	5.66289500	1.35223500
C	3.95885800	5.58670600	-1.48079000
C	4.00521700	5.55560400	-2.95207100
C	5.04047400	4.65213000	-4.98677500
H	4.44551500	5.46755000	-5.39070300
H	4.66922500	3.69255400	-5.35462700
H	6.08845400	4.75002800	-5.27303400
H	3.69255400	4.66922500	5.35462700
C	6.25763900	2.92830700	0.84061200

C	6.29381600	2.82906900	-0.58910700
C	2.82906900	6.29381600	0.58910700
C	2.92830700	6.25763900	-0.84061200
C	6.80343800	1.56438700	-1.01567700
C	7.07080800	0.72180900	0.03282500
S	6.93795700	1.51694900	1.60403400
H	6.80788700	1.26126900	-2.05418900
C	1.56438700	6.80343800	1.01567700
C	0.72180900	7.07080800	-0.03282500
S	1.51694900	6.93795700	-1.60403400
H	1.26126900	6.80788700	2.05418900

$B_4^{2-\bullet\bullet}$ (OSS)
(U)M06-2X(D3)//6-31+G(d)
E = -7893.633650 a.u.

O	2.62406500	6.58340300	3.64580900
N	0.35607000	6.83697300	3.51657900
O	2.95430400	6.52005600	-3.44070000
N	0.71108900	6.81021600	-3.51620400
C	0.24721200	6.88840700	4.97032900
H	-0.34992200	7.75649900	5.25523500
H	1.25346300	6.95120700	5.37782300
O	-1.90500500	6.89909900	3.44092700
O	-1.56865000	6.91245900	-3.64542500
C	1.61874300	6.68515200	2.94633400
C	1.67395100	6.66111800	1.48835000
C	1.82129200	6.60232300	-1.34985700
C	1.90630700	6.62955800	-2.81042600
C	0.48554500	6.78817400	0.71903500
C	0.57568500	6.78141000	-0.71862400
C	-0.85266100	6.84441900	2.81074200
C	-0.77281700	6.80461200	1.35020200
C	-0.61803000	6.84081000	-1.48794200
C	-0.55976500	6.85654800	-2.94592300
C	0.82640700	6.84425900	-4.96995300
H	-0.15770400	7.06404800	-5.37709600
H	1.18403200	5.87903500	-5.33781100
H	1.55215100	7.60810000	-5.25480000
H	-0.25743800	5.99113100	5.33784800
C	2.86322100	6.30915200	0.84392600
C	2.95878100	6.25132800	-0.57298500
C	-1.95098700	6.63478000	0.57324100
C	-1.84750700	6.67748300	-0.84368600
C	4.15829800	5.61106900	-0.99535400
C	4.92118600	5.15173600	0.05460200
S	4.28833600	5.66945600	1.61653500
H	4.35449700	5.37969600	-2.03448400
C	-3.23543700	6.18868900	0.99550900
C	-4.06035500	5.85350700	-0.05451600
S	-3.35460600	6.26681600	-1.61650400
H	-3.46519900	5.99063700	2.03467600
O	-6.59420900	2.64991000	3.62476300
N	-6.82054900	0.38188000	3.52698500
O	-6.54453400	2.92656000	-3.46081800
N	-6.79625500	0.68237700	-3.52729900
C	-6.86772100	0.28293900	4.98334400
H	-7.76403200	-0.26532400	5.27924500

H	-6.87672100	1.29277100	5.38619800
O	-6.92048500	-1.87418500	3.46045300
O	-6.92515700	-1.59319700	-3.62512500
C	-6.68091700	1.63775200	2.94451800
C	-6.65380800	1.68358000	1.47271800
C	-6.60654900	1.81474200	-1.35733500
C	-6.63491800	1.88494700	-2.82910900
C	-6.80390600	0.49165900	0.71251600
C	-6.79755400	0.57144700	-0.71281300
C	-6.84771700	-0.83115900	2.82880200
C	-6.80848300	-0.76621400	1.35704300
C	-6.83447100	-0.62933500	-1.47300900
C	-6.85381100	-0.57987900	-2.94482000
C	-6.82658500	0.78728100	-4.98367100
H	-6.99237500	-0.20893900	-5.38641800
H	-5.87830300	1.19303800	-5.34342200
H	-7.62644100	1.46833900	-5.28016000
H	-5.99441200	-0.26549300	5.34368200
C	-6.30612400	2.85998000	0.82910300
C	-6.25311000	2.94779600	-0.60329400
C	-6.63540800	-1.94043500	0.60301700
C	-6.67407000	-1.84544700	-0.82939500
C	-5.62601400	4.15423900	-1.03534000
C	-5.14622400	4.90760600	0.00893500
S	-5.67429000	4.29954200	1.58333600
H	-5.39502000	4.35046200	-2.07359000
C	-6.20367900	-3.22972100	1.03517000
C	-5.84685600	-4.04856300	-0.00902700
S	-6.27411600	-3.36598900	-1.58349400
H	-6.00592400	-3.45930900	2.07345700
O	-2.62406500	-6.58340300	3.64580900
N	-0.35607000	-6.83697300	3.51657900
O	-2.95430400	-6.52005600	-3.44070000
N	-0.71108900	-6.81021600	-3.51620400
C	-0.24721200	-6.88840700	4.97032900
H	0.34992200	-7.75649900	5.25523500
H	-1.25346300	-6.95120700	5.37782300
O	1.90500500	-6.89909900	3.44092700
O	1.56865000	-6.91245900	-3.64542500
C	-1.61874300	-6.68515200	2.94633400
C	-1.67395100	-6.66111800	1.48835000
C	-1.82129200	-6.60232300	-1.34985700
C	-1.90630700	-6.62955800	-2.81042600
C	-0.48554500	-6.78817400	0.71903500
C	-0.57568500	-6.78141000	-0.71862400
C	0.85266100	-6.84441900	2.81074200
C	0.77281700	-6.80461200	1.35020200
C	0.61803000	-6.84081000	-1.48794200
C	0.55976500	-6.85654800	-2.94592300
C	-0.82640700	-6.84425900	-4.96995300
H	0.15770400	-7.06404800	-5.37709600
H	-1.18403200	-5.87903500	-5.33781100
H	-1.55215100	-7.60810000	-5.25480000
H	0.25743800	-5.99113100	5.33784800
C	-2.86322100	-6.30915200	0.84392600
C	-2.95878100	-6.25132800	-0.57298500
C	1.95098700	-6.63478000	0.57324100

C	1.84750700	-6.67748300	-0.84368600
C	-4.15829800	-5.61106900	-0.99535400
C	-4.92118600	-5.15173600	0.05460200
S	-4.28833600	-5.66945600	1.61653500
H	-4.35449700	-5.37969600	-2.03448400
C	3.23543700	-6.18868900	0.99550900
C	4.06035500	-5.85350700	-0.05451600
S	3.35460600	-6.26681600	-1.61650400
H	3.46519900	-5.99063700	2.03467600
O	6.59420900	-2.64991000	3.62476300
N	6.82054900	-0.38188000	3.52698500
O	6.54453400	-2.92656000	-3.46081800
N	6.79625500	-0.68237700	-3.52729900
C	6.86772100	-0.28293900	4.98334400
H	7.76403200	0.26532400	5.27924500
H	6.87672100	-1.29277100	5.38619800
O	6.92048500	1.87418500	3.46045300
O	6.92515700	1.59319700	-3.62512500
C	6.68091700	-1.63775200	2.94451800
C	6.65380800	-1.68358000	1.47271800
C	6.60654900	-1.81474200	-1.35733500
C	6.63491800	-1.88494700	-2.82910900
C	6.80390600	-0.49165900	0.71251600
C	6.79755400	-0.57144700	-0.71281300
C	6.84771700	0.83115900	2.82880200
C	6.80848300	0.76621400	1.35704300
C	6.83447100	0.62933500	-1.47300900
C	6.85381100	0.57987900	-2.94482000
C	6.82658500	-0.78728100	-4.98367100
H	6.99237400	0.20893900	-5.38641800
H	5.87830300	-1.19303900	-5.34342200
H	7.62644100	-1.46833900	-5.28016000
H	5.99441200	0.26549300	5.34368200
C	6.30612400	-2.85998000	0.82910300
C	6.25311000	-2.94779600	-0.60329400
C	6.63540800	1.94043500	0.60301700
C	6.67407000	1.84544700	-0.82939500
C	5.62601400	-4.15423900	-1.03534000
C	5.14622400	-4.90760600	0.00893500
S	5.67429000	-4.29954200	1.58333600
H	5.39502000	-4.35046200	-2.07359000
C	6.20367900	3.22972100	1.03517000
C	5.84685600	4.04856300	-0.00902700
S	6.27411600	3.36598900	-1.58349400
H	6.00592400	3.45930900	2.07345700

$B_4^{2-\bullet\bullet}$ (triplet)
(U)M06-2X(D3)//6-31+G(d)
E = -7893.630929 a.u.

O	6.77650000	2.09393400	-3.65084900
N	6.85411400	-0.18722500	-3.51412500
O	6.73529400	2.44756400	3.43510000
N	6.85052400	0.18794200	3.51478700
C	6.89906400	-0.30280400	-4.96731200
H	7.71370900	-0.97238600	-5.24899100
H	7.04780900	0.69374400	-5.37641100
O	6.73745900	-2.44678600	-3.43467600

O	6.77198400	-2.09321500	3.65136500
C	6.80047200	1.08546700	-2.94702600
C	6.78020500	1.14575400	-1.49135000
C	6.72947000	1.30481800	1.34782100
C	6.76333300	1.39183000	2.80667000
C	6.81100800	-0.04594700	-0.71916200
C	6.81039000	0.04663000	0.71971100
C	6.76549500	-1.39111900	-2.80613900
C	6.73046900	-1.30414900	-1.34735000
C	6.77873300	-1.14503700	1.49192700
C	6.79719000	-1.08474000	2.94759900
C	6.89382300	0.30361500	4.96801000
H	7.03940100	-0.69325500	5.37745200
H	5.95839700	0.73354600	5.33570600
H	7.70985500	0.97108300	5.25078900
H	5.96300500	-0.73012400	-5.33646100
C	6.52116200	2.36256800	-0.84759900
C	6.46891300	2.46387000	0.56518300
C	6.46917200	-2.46316700	-0.56487600
C	6.52037300	-2.36183000	0.84799900
C	5.91697900	3.71027200	0.98565000
C	5.52326600	4.50439600	-0.06505400
S	5.98962500	3.83009000	-1.62423300
H	5.69895200	3.92298200	2.02458100
C	5.91737300	-3.70950400	-0.98564300
C	5.52298400	-4.50365900	0.06484200
S	5.98841600	-3.82926900	1.62431500
H	5.69996400	-3.92216200	-2.02471300
O	2.13351300	-6.78455400	-3.62006500
N	-0.14518400	-6.82509200	-3.52903400
O	2.40211600	-6.75692500	3.46519300
N	0.14523400	-6.82487900	3.52865600
C	-0.24481000	-6.86292500	-4.98593200
H	-0.84342100	-7.72526600	-5.28563300
H	0.76326700	-6.92985500	-5.38763400
O	-2.40208200	-6.75835300	-3.46555800
O	-2.13342900	-6.78425300	3.61969200
C	1.11635500	-6.78846000	-2.94343900
C	1.16193000	-6.76449200	-1.46962000
C	1.29336500	-6.72788800	1.35906600
C	1.35804200	-6.76178400	2.83290600
C	-0.03829000	-6.82514500	-0.71157900
C	0.03829600	-6.82508800	0.71120900
C	-1.35800300	-6.76245600	-2.83328600
C	-1.29336400	-6.72831400	-1.35945000
C	-1.16186700	-6.76470500	1.46923500
C	-1.11627600	-6.78814000	2.94306000
C	0.24481000	-6.86261100	4.98555300
H	-0.76311800	-6.93220800	5.38715000
H	0.73879600	-5.95658700	5.34332800
H	0.84568900	-7.72338500	5.28510700
H	-0.74126500	-5.95816500	-5.34343700
C	2.36021800	-6.51010700	-0.82471000
C	2.44826100	-6.46349900	0.60896600
C	-2.44837100	-6.46419400	-0.60935000
C	-2.36027600	-6.51064500	0.82427900
C	3.70242100	-5.93060900	1.04387100

C	4.48929200	-5.51209000	0.00198000
S	3.84773300	-5.99379600	-1.57452200
H	3.91327300	-5.71203600	2.08182500
C	-3.70262100	-5.93138100	-1.04426900
C	-4.48943800	-5.51276500	-0.00237900
S	-3.84753800	-5.99388500	1.57418600
H	-3.91352600	-5.71302400	-2.08226100
O	-6.77650000	-2.09393400	-3.65084900
N	-6.85411400	0.18722500	-3.51412500
O	-6.73529400	-2.44756400	3.43510000
N	-6.85052400	-0.18794200	3.51478700
C	-6.89906400	0.30280400	-4.96731200
H	-7.71370900	0.97238600	-5.24899100
H	-7.04780900	-0.69374400	-5.37641100
O	-6.73745900	2.44678600	-3.43467600
O	-6.77198400	2.09321500	3.65136500
C	-6.80047200	-1.08546700	-2.94702600
C	-6.78020500	-1.14575400	-1.49135000
C	-6.72947000	-1.30481800	1.34782100
C	-6.76333300	-1.39183000	2.80667000
C	-6.81100800	0.04594700	-0.71916200
C	-6.81039000	-0.04663000	0.71971100
C	-6.76549500	1.39111900	-2.80613900
C	-6.73046900	1.30414900	-1.34735000
C	-6.77873300	1.14503700	1.49192700
C	-6.79719000	1.08474000	2.94759900
C	-6.89382300	-0.30361500	4.96801000
H	-7.03940100	0.69325500	5.37745200
H	-5.95839700	-0.73354600	5.33570600
H	-7.70985500	-0.97108300	5.25078900
H	-5.96300500	0.73012400	-5.33646100
C	-6.52116200	-2.36256800	-0.84759900
C	-6.46891300	-2.46387000	0.56518300
C	-6.46917200	2.46316700	-0.56487600
C	-6.52037300	2.36183000	0.84799900
C	-5.91697900	-3.71027200	0.98565000
C	-5.52326600	-4.50439600	-0.06505400
S	-5.98962500	-3.83009000	-1.62423300
H	-5.69895200	-3.92298200	2.02458100
C	-5.91737300	3.70950400	-0.98564300
C	-5.52298400	4.50365900	0.06484200
S	-5.98841600	3.82926900	1.62431500
H	-5.69996400	3.92216200	-2.02471300
O	-2.13351300	6.78455400	-3.62006500
N	0.14518400	6.82509200	-3.52903400
O	-2.40211600	6.75692500	3.46519300
N	-0.14523400	6.82487900	3.52865600
C	0.24481000	6.86292500	-4.98593200
H	0.84342100	7.72526600	-5.28563300
H	-0.76326700	6.92985500	-5.38763400
O	2.40208200	6.75835300	-3.46555800
O	2.13342900	6.78425300	3.61969200
C	-1.11635500	6.78846000	-2.94343900
C	-1.16193000	6.76449200	-1.46962000
C	-1.29336500	6.72788800	1.35906600
C	-1.35804200	6.76178400	2.83290600
C	0.03829000	6.82514500	-0.71157900

C	-0.03829600	6.82508800	0.71120900
C	1.35800300	6.76245600	-2.83328600
C	1.29336400	6.72831400	-1.35945000
C	1.16186700	6.76470500	1.46923500
C	1.11627600	6.78814000	2.94306000
C	-0.24481000	6.86261100	4.98555300
H	0.76311800	6.93220800	5.38715000
H	-0.73879600	5.95658700	5.34332800
H	-0.84568900	7.72338500	5.28510700
H	0.74126500	5.95816500	-5.34343700
C	-2.36021800	6.51010700	-0.82471000
C	-2.44826100	6.46349900	0.60896600
C	2.44837100	6.46419400	-0.60935000
C	2.36027600	6.51064500	0.82427900
C	-3.70242100	5.93060900	1.04387100
C	-4.48929200	5.51209000	0.00198000
S	-3.84773300	5.99379600	-1.57452200
H	-3.91327300	5.71203600	2.08182500
C	3.70262100	5.93138100	-1.04426900
C	4.48943800	5.51276500	-0.00237900
S	3.84753800	5.99388500	1.57418600
H	3.91352600	5.71302400	-2.08226100

A_2B_2 (singlet)
M06-2X(D3)//6-31+G(d)
E = -7893.429370 a.u.

O	3.04293800	3.77195100	6.09053500
N	4.71104800	3.69918600	4.53645200
O	3.21662200	-3.28794200	6.64937800
N	4.88293200	-3.35741200	5.12481000
C	4.72986500	5.15857800	4.42201000
H	5.76225800	5.50513100	4.47558200
H	4.13760200	5.56604400	5.23750800
O	6.20763000	3.61524600	2.84553500
O	6.42677200	-3.44232700	3.44791100
C	3.82121800	3.10917900	5.42660400
C	3.86461700	1.63537000	5.53469300
C	2.87752600	0.97153200	6.24026000
C	2.86313500	-0.46157900	6.34190700
C	3.89511900	-1.19238000	5.75209700
C	3.94249100	-2.66970300	5.89235500
C	4.79297000	0.88269800	4.77114500
C	4.81828700	-0.53720400	4.90216600
C	5.53616300	3.00015000	3.65420100
C	5.56103500	1.52084400	3.76933900
C	6.22405400	0.74883900	2.81036300
C	6.27389900	-0.67655400	2.98181000
C	5.64858300	-1.30110700	4.04615900
C	5.69915100	-2.77511600	4.16230800
C	4.92494100	-4.81023800	5.30186000
H	5.75779000	-5.19465900	4.71847800
H	3.98590700	-5.25142900	4.96043000
H	5.05152000	-5.03581300	6.36122600
H	4.30919000	5.46086500	3.46020700
C	6.70030100	1.13983800	1.51390000
C	7.01468700	0.06694100	0.73049900
S	6.97718800	-1.46815600	1.59979100

H	6.65223100	2.16042600	1.16111100
C	1.61496100	-0.93766600	6.87294800
C	0.72450300	0.07182300	7.09172500
S	1.42488700	1.68165900	6.89247500
H	1.37124500	-1.99042800	6.92621600
O	-6.42677200	3.44232700	3.44791100
N	-4.88293200	3.35741200	5.12481000
O	-6.20763000	-3.61524600	2.84553500
N	-4.71104800	-3.69918600	4.53645200
C	-4.92494100	4.81023800	5.30186000
H	-5.05152000	5.03581300	6.36122600
H	-5.75779000	5.19465900	4.71847800
O	-3.21662200	3.28794200	6.64937800
O	-3.04293800	-3.77195100	6.09053500
C	-5.69915100	2.77511600	4.16230800
C	-5.64858300	1.30110700	4.04615900
C	-6.27389900	0.67655400	2.98181000
C	-6.22405400	-0.74883900	2.81036300
C	-5.56103500	-1.52084400	3.76933900
C	-5.53616300	-3.00015000	3.65420100
C	-4.81828700	0.53720400	4.90216600
C	-4.79297000	-0.88269800	4.77114500
C	-3.94249100	2.66970300	5.89235500
C	-3.89511900	1.19238000	5.75209700
C	-2.86313500	0.46157900	6.34190700
C	-2.87752600	-0.97153200	6.24026000
C	-3.86461700	-1.63537000	5.53469300
C	-3.82121800	-3.10917900	5.42660400
C	-4.72986500	-5.15857800	4.42201000
H	-4.13760200	-5.56604400	5.23750800
H	-4.30919000	-5.46086500	3.46020700
H	-5.76225800	-5.50513100	4.47558200
H	-3.98590700	5.25142900	4.96043000
C	-1.61496100	0.93766600	6.87294800
C	-0.72450300	-0.07182300	7.09172500
S	-1.42488700	-1.68165900	6.89247500
H	-1.37124500	1.99042800	6.92621600
C	-6.70030100	-1.13983800	1.51390000
C	-7.01468700	-0.06694100	0.73049900
S	-6.97718800	1.46815600	1.59979100
H	-6.65223100	-2.16042600	1.16111100
O	-3.04293800	-3.77195100	-6.09053500
N	-4.71104800	-3.69918600	-4.53645200
O	-3.21662200	3.28794200	-6.64937800
N	-4.88293200	3.35741200	-5.12481000
C	-4.72986500	-5.15857800	-4.42201000
H	-4.30919000	-5.46086500	-3.46020700
H	-4.13760200	-5.56604400	-5.23750800
O	-6.20763000	-3.61524600	-2.84553500
O	-6.42677200	3.44232700	-3.44791100
C	-3.82121800	-3.10917900	-5.42660400
C	-3.86461700	-1.63537000	-5.53469300
C	-2.87752600	-0.97153200	-6.24026000
C	-2.86313500	0.46157900	-6.34190700
C	-3.89511900	1.19238000	-5.75209700
C	-3.94249100	2.66970300	-5.89235500
C	-4.79297000	-0.88269800	-4.77114500

C	-4.81828700	0.53720400	-4.90216600
C	-5.53616300	-3.00015000	-3.65420100
C	-5.56103500	-1.52084400	-3.76933900
C	-6.22405400	-0.74883900	-2.81036300
C	-6.27389900	0.67655400	-2.98181000
C	-5.64858300	1.30110700	-4.04615900
C	-5.69915100	2.77511600	-4.16230800
C	-4.92494100	4.81023800	-5.30186000
H	-3.98590700	5.25142900	-4.96043000
H	-5.75779000	5.19465900	-4.71847800
H	-5.05152000	5.03581300	-6.36122600
H	-5.76225800	-5.50513100	-4.47558200
C	-6.70030100	-1.13983800	-1.51390000
C	-7.01468700	-0.06694100	-0.73049900
S	-6.97718800	1.46815600	-1.59979100
H	-6.65223100	-2.16042600	-1.16111100
C	-1.61496100	0.93766600	-6.87294800
C	-0.72450300	-0.07182300	-7.09172500
S	-1.42488700	-1.68165900	-6.89247500
H	-1.37124500	1.99042800	-6.92621600
O	6.42677200	-3.44232700	-3.44791100
N	4.88293200	-3.35741200	-5.12481000
O	6.20763000	3.61524600	-2.84553500
N	4.71104800	3.69918600	-4.53645200
C	4.92494100	-4.81023800	-5.30186000
H	3.98590700	-5.25142900	-4.96043000
H	5.75779000	-5.19465900	-4.71847800
O	3.21662200	-3.28794200	-6.64937800
O	3.04293800	3.77195100	-6.09053500
C	5.69915100	-2.77511600	-4.16230800
C	5.64858300	-1.30110700	-4.04615900
C	6.27389900	-0.67655400	-2.98181000
C	6.22405400	0.74883900	-2.81036300
C	5.56103500	1.52084400	-3.76933900
C	5.53616300	3.00015000	-3.65420100
C	4.81828700	-0.53720400	-4.90216600
C	4.79297000	0.88269800	-4.77114500
C	3.94249100	-2.66970300	-5.89235500
C	3.89511900	-1.19238000	-5.75209700
C	2.86313500	-0.46157900	-6.34190700
C	2.87752600	0.97153200	-6.24026000
C	3.86461700	1.63537000	-5.53469300
C	3.82121800	3.10917900	-5.42660400
C	4.72986500	5.15857800	-4.42201000
H	4.13760200	5.56604400	-5.23750800
H	5.76225800	5.50513100	-4.47558200
H	4.30919000	5.46086500	-3.46020700
H	5.05152000	-5.03581300	-6.36122600
C	1.61496100	-0.93766600	-6.87294800
C	0.72450300	0.07182300	-7.09172500
S	1.42488700	1.68165900	-6.89247500
H	1.37124500	-1.99042800	-6.92621600
C	6.70030100	1.13983800	-1.51390000
C	7.01468700	0.06694100	-0.73049900
S	6.97718800	-1.46815600	-1.59979100
H	6.65223100	2.16042600	-1.16111100

$A_2B_2^{-\bullet}$

(U)M06-2X(D3)//6-31+G(d)

E = -7893.559200 a.u.

O	-3.05356900	-3.77407900	6.11261900
N	-4.70842000	-3.69473500	4.54333500
O	-3.22875100	3.29225600	6.64071200
N	-4.90228300	3.34600300	5.12264100
C	-4.72173600	-5.15158500	4.42501100
H	-5.75112200	-5.50574700	4.49403100
H	-4.11245100	-5.55770700	5.22869200
O	-6.20525600	-3.62446400	2.85132800
O	-6.46511100	3.43486700	3.46219700
C	-3.82331700	-3.10462300	5.43942700
C	-3.86488600	-1.63631300	5.53851100
C	-2.87694100	-0.96806100	6.24514800
C	-2.85949600	0.46239100	6.34327600
C	-3.90097700	1.19288000	5.74646200
C	-3.95324500	2.66149900	5.88708200
C	-4.78870000	-0.88691400	4.76214600
C	-4.81582600	0.53775000	4.89372200
C	-5.53546800	-2.99766400	3.65746000
C	-5.56095800	-1.52640600	3.76919400
C	-6.23368400	-0.75441300	2.80337900
C	-6.29042000	0.66759700	2.97999100
C	-5.66327000	1.29608300	4.04502300
C	-5.72510400	2.76263900	4.16498200
C	-4.94339700	4.79678400	5.29677400
H	-5.77007100	5.18113300	4.70440800
H	-3.99998100	5.23622300	4.96450900
H	-5.07797400	5.02635900	6.35475800
H	-4.31764100	-5.44897100	3.45438000
C	-6.70783200	-1.14506000	1.51583800
C	-7.03503300	-0.07223500	0.72458400
S	-7.00281900	1.45838400	1.60310100
H	-6.65343900	-2.16496700	1.16174300
C	-1.62001600	0.93818600	6.87060000
C	-0.71912600	-0.07069000	7.09623400
S	-1.42568200	-1.67804600	6.89867800
H	-1.37680900	1.99131500	6.92040800
O	6.46511100	-3.43486700	3.46219700
N	4.90228300	-3.34600300	5.12264100
O	6.20525600	3.62446400	2.85132800
N	4.70842000	3.69473500	4.54333500
C	4.94339700	-4.79678400	5.29677400
H	5.07797400	-5.02635900	6.35475800
H	5.77007100	-5.18113300	4.70440800
O	3.22875100	-3.29225600	6.64071200
O	3.05356900	3.77407900	6.11261900
C	5.72510400	-2.76263900	4.16498200
C	5.66327000	-1.29608300	4.04502300
C	6.29042000	-0.66759700	2.97999100
C	6.23368400	0.75441300	2.80337900
C	5.56095800	1.52640600	3.76919400
C	5.53546800	2.99766400	3.65746000
C	4.81582600	-0.53775000	4.89372200
C	4.78870000	0.88691400	4.76214600
C	3.95324500	-2.66149900	5.88708200

C	3.90097700	-1.19288000	5.74646200
C	2.85949600	-0.46239100	6.34327600
C	2.87694100	0.96806100	6.24514800
C	3.86488600	1.63631300	5.53851100
C	3.82331700	3.10462300	5.43942700
C	4.72173600	5.15158500	4.42501100
H	4.11245100	5.55770700	5.22869200
H	4.31764100	5.44897100	3.45438000
H	5.75112200	5.50574700	4.49403100
H	3.99998100	-5.23622300	4.96450900
C	1.62001600	-0.93818600	6.87060000
C	0.71912600	0.07069000	7.09623400
S	1.42568200	1.67804600	6.89867800
H	1.37680900	-1.99131500	6.92040800
C	6.70783200	1.14506000	1.51583800
C	7.03503300	0.07223500	0.72458400
S	7.00281900	-1.45838400	1.60310100
H	6.65343900	2.16496700	1.16174300
O	3.05356900	3.77407900	-6.11261900
N	4.70842000	3.69473500	-4.54333500
O	3.22875100	-3.29225600	-6.64071200
N	4.90228300	-3.34600300	-5.12264100
C	4.72173600	5.15158500	-4.42501100
H	4.31764100	5.44897100	-3.45438000
H	4.11245100	5.55770700	-5.22869200
O	6.20525600	3.62446400	-2.85132800
O	6.46511100	-3.43486700	-3.46219700
C	3.82331700	3.10462300	-5.43942700
C	3.86488600	1.63631300	-5.53851100
C	2.87694100	0.96806100	-6.24514800
C	2.85949600	-0.46239100	-6.34327600
C	3.90097700	-1.19288000	-5.74646200
C	3.95324500	-2.66149900	-5.88708200
C	4.78870000	0.88691400	-4.76214600
C	4.81582600	-0.53775000	-4.89372200
C	5.53546800	2.99766400	-3.65746000
C	5.56095800	1.52640600	-3.76919400
C	6.23368400	0.75441300	-2.80337900
C	6.29042000	-0.66759700	-2.97999100
C	5.66327000	-1.29608300	-4.04502300
C	5.72510400	-2.76263900	-4.16498200
C	4.94339700	-4.79678400	-5.29677400
H	3.99998100	-5.23622300	-4.96450900
H	5.77007100	-5.18113300	-4.70440800
H	5.07797400	-5.02635900	-6.35475800
H	5.75112200	5.50574700	-4.49403100
C	6.70783200	1.14506000	-1.51583800
C	7.03503300	0.07223500	-0.72458400
S	7.00281900	-1.45838400	-1.60310100
H	6.65343900	2.16496700	-1.16174300
C	1.62001600	-0.93818600	-6.87060000
C	0.71912600	0.07069000	-7.09623400
S	1.42568200	1.67804600	-6.89867800
H	1.37680900	-1.99131500	-6.92040800
O	-6.46511100	3.43486700	-3.46219700
N	-4.90228300	3.34600300	-5.12264100
O	-6.20525600	-3.62446400	-2.85132800

N	-4.70842000	-3.69473500	-4.54333500
C	-4.94339700	4.79678400	-5.29677400
H	-3.99998100	5.23622300	-4.96450900
H	-5.77007100	5.18113300	-4.70440800
O	-3.22875100	3.29225600	-6.64071200
O	-3.05356900	-3.77407900	-6.11261900
C	-5.72510400	2.76263900	-4.16498200
C	-5.66327000	1.29608300	-4.04502300
C	-6.29042000	0.66759700	-2.97999100
C	-6.23368400	-0.75441300	-2.80337900
C	-5.56095800	-1.52640600	-3.76919400
C	-5.53546800	-2.99766400	-3.65746000
C	-4.81582600	0.53775000	-4.89372200
C	-4.78870000	-0.88691400	-4.76214600
C	-3.95324500	2.66149900	-5.88708200
C	-3.90097700	1.19288000	-5.74646200
C	-2.85949600	0.46239100	-6.34327600
C	-2.87694100	-0.96806100	-6.24514800
C	-3.86488600	-1.63631300	-5.53851100
C	-3.82331700	-3.10462300	-5.43942700
C	-4.72173600	-5.15158500	-4.42501100
H	-4.11245100	-5.55770700	-5.22869200
H	-5.75112200	-5.50574700	-4.49403100
H	-4.31764100	-5.44897100	-3.45438000
H	-5.07797400	5.02635900	-6.35475800
C	-1.62001600	0.93818600	-6.87060000
C	-0.71912600	-0.07069000	-7.09623400
S	-1.42568200	-1.67804600	-6.89867800
H	-1.37680900	1.99131500	-6.92040800
C	-6.70783200	-1.14506000	-1.51583800
C	-7.03503300	-0.07223500	-0.72458400
S	-7.00281900	1.45838400	-1.60310100
H	-6.65343900	-2.16496700	-1.16174300

$A_2B_2^{2-\bullet\bullet}$ (OSS)
(U)M06-2X(D3)//6-31+G(d)
E = -7893.627699 a.u.

O	-5.67532500	3.87908600	-3.77521700
N	-3.89029900	5.30338400	-3.68096300
O	-6.14697200	4.10520500	3.29912800
N	-4.42501100	5.57330200	3.33526800
C	-3.77506500	5.30425700	-5.13514000
H	-3.69945000	6.33421400	-5.48799300
H	-4.65632800	4.81205800	-5.53970100
O	-2.00116900	6.54854100	-3.62601400
O	-2.56985500	6.90451800	3.43853100
C	-4.89973200	4.54417000	-3.09244100
C	-4.98133100	4.59040200	-1.63590600
C	-5.81812500	3.69931500	-0.95865500
C	-5.90943700	3.68804100	0.46002800
C	-5.16751100	4.64790000	1.19949500
C	-5.30457400	4.72359000	2.65476900
C	-4.06966900	5.38367200	-0.88751900
C	-4.19408000	5.42812000	0.54714200
C	-2.89098600	5.99523000	-2.98583300
C	-2.99183600	6.02526600	-1.52677600
C	-1.93282700	6.56721100	-0.74641000

C	-2.10087900	6.65253800	0.66370600
C	-3.24897400	6.17443700	1.30108800
C	-3.36117700	6.26064400	2.75356000
C	-4.59288700	5.64091200	4.78270500
H	-3.90115900	6.38847500	5.16375900
H	-4.38234800	4.66507000	5.22797100
H	-5.62677700	5.90493000	5.01222700
H	-2.86961100	4.77178600	-5.43798800
C	-0.59569100	6.84932100	-1.13777500
C	0.24064800	7.08516200	-0.06815200
S	-0.64040900	7.16634000	1.45696300
H	-0.25629300	6.73784800	-2.15867700
C	-6.58374600	2.52791000	0.93638800
C	-6.94964600	1.66580300	-0.07226900
S	-6.65465100	2.34771600	-1.67240500
H	-6.65412600	2.29324000	1.99093800
O	-4.35316900	-5.98027700	-3.42723000
N	-5.76395200	-4.18971300	-3.33609900
O	-3.69266300	-5.78695200	3.63242800
N	-5.14757200	-4.05938400	3.69574400
C	-5.94704800	-4.20859600	-4.78486800
H	-7.01373100	-4.24603000	-5.01371900
H	-5.43306200	-5.08376600	-5.17473300
O	-7.05992600	-2.33971100	-3.28672300
O	-6.50137000	-2.22486800	3.78059400
C	-4.93060600	-5.13936800	-2.75319400
C	-4.78930400	-5.08330900	-1.28836700
C	-3.81601900	-5.84533700	-0.66296700
C	-3.62681300	-5.81039200	0.76065200
C	-4.49732800	-5.01463400	1.53016900
C	-4.38790500	-5.00703600	2.99884300
C	-5.51553400	-4.12469800	-0.53127000
C	-5.37765700	-4.11350200	0.89019800
C	-6.39783100	-3.14624100	-2.65141800
C	-6.24080800	-3.10413600	-1.18687900
C	-6.68683500	-1.98556900	-0.46126500
C	-6.58858500	-2.01597700	0.97091600
C	-6.02199400	-3.09001300	1.63808600
C	-5.92470800	-3.06694400	3.10708000
C	-5.02610700	-4.08667900	5.15086600
H	-5.69970400	-3.33553000	5.55605500
H	-3.99470500	-3.87087800	5.43916800
H	-5.28362300	-5.08268100	5.51552200
H	-5.53365200	-3.29594300	-5.22011300
C	-7.04412700	-0.68951500	-0.93872500
C	-7.13270700	0.24255200	0.06673900
S	-7.03712100	-0.48575700	1.67637300
H	-7.06012300	-0.44187400	-1.99160300
C	-2.41764400	-6.45442600	1.14990800
C	-1.67003000	-6.87907000	0.07575900
S	-2.54655500	-6.73812100	-1.45309600
H	-2.05604700	-6.44651800	2.16839900
O	5.67532400	-3.87908600	3.77521700
N	3.89029900	-5.30338400	3.68096300
O	6.14697200	-4.10520500	-3.29912800
N	4.42501100	-5.57330200	-3.33526800
C	3.77506500	-5.30425600	5.13514000

H	2.86961000	-4.77178500	5.43798800
H	4.65632800	-4.81205800	5.53970100
O	2.00116900	-6.54854100	3.62601400
O	2.56985500	-6.90451800	-3.43853100
C	4.89973200	-4.54417000	3.09244100
C	4.98133100	-4.59040200	1.63590600
C	5.81812500	-3.69931500	0.95865500
C	5.90943700	-3.68804100	-0.46002700
C	5.16751100	-4.64790000	-1.19949500
C	5.30457400	-4.72359100	-2.65476800
C	4.06966900	-5.38367200	0.88751900
C	4.19408000	-5.42812000	-0.54714200
C	2.89098600	-5.99523000	2.98583300
C	2.99183500	-6.02526600	1.52677600
C	1.93282700	-6.56721100	0.74641000
C	2.10087900	-6.65253800	-0.66370600
C	3.24897400	-6.17443700	-1.30108800
C	3.36117700	-6.26064400	-2.75356000
C	4.59288700	-5.64091300	-4.78270400
H	4.38234800	-4.66507000	-5.22797100
H	3.90115900	-6.38847600	-5.16375900
H	5.62677700	-5.90493000	-5.01222700
H	3.69945000	-6.33421300	5.48799300
C	0.59569100	-6.84932100	1.13777500
C	-0.24064800	-7.08516200	0.06815200
S	0.64040900	-7.16634000	-1.45696300
H	0.25629300	-6.73784800	2.15867700
C	6.58374600	-2.52791000	-0.93638800
C	6.94964600	-1.66580300	0.07226900
S	6.65465000	-2.34771600	1.67240500
H	6.65412600	-2.29324100	-1.99093800
O	4.35316900	5.98027700	3.42723000
N	5.76395200	4.18971300	3.33609900
O	3.69266300	5.78695100	-3.63242800
N	5.14757200	4.05938400	-3.69574400
C	5.94704800	4.20859600	4.78486800
H	5.53365200	3.29594300	5.22011300
H	5.43306300	5.08376700	5.17473300
O	7.05992600	2.33971100	3.28672300
O	6.50137000	2.22486800	-3.78059400
C	4.93060600	5.13936900	2.75319400
C	4.78930400	5.08330900	1.28836700
C	3.81601900	5.84533700	0.66296700
C	3.62681300	5.81039200	-0.76065300
C	4.49732800	5.01463400	-1.53016900
C	4.38790500	5.00703500	-2.99884300
C	5.51553400	4.12469800	0.53126900
C	5.37765700	4.11350200	-0.89019800
C	6.39783200	3.14624100	2.65141800
C	6.24080800	3.10413600	1.18687900
C	6.68683500	1.98556900	0.46126400
C	6.58858500	2.01597700	-0.97091600
C	6.02199400	3.09001300	-1.63808600
C	5.92470800	3.06694300	-3.10708000
C	5.02610700	4.08667900	-5.15086700
H	5.69970400	3.33553000	-5.55605600
H	5.28362300	5.08268100	-5.51552200

H	3.99470500	3.87087800	-5.43916800
H	7.01373200	4.24603100	5.01371800
C	7.04412700	0.68951500	0.93872400
C	7.13270700	-0.24255200	-0.06673900
S	7.03712100	0.48575700	-1.67637300
H	7.06012400	0.44187400	1.99160300
C	2.41764400	6.45442600	-1.14990800
C	1.67003000	6.87907000	-0.07575900
S	2.54655500	6.73812100	1.45309600
H	2.05604700	6.44651700	-2.16839900

$A_2B_2^{2-\bullet\bullet}$ (triplet)
(U)M06-2X(D3)//6-31+G(d)
E = -7893.624848 a.u.

O	3.07567000	3.79676900	6.14957400
N	4.73426500	3.70532800	4.57838700
O	3.25403900	-3.27695800	6.65255200
N	4.94084900	-3.30788900	5.14282900
C	4.74806800	5.15937500	4.46605300
H	5.77824300	5.51396000	4.53033900
H	4.14049300	5.56174700	5.27327400
O	6.22667700	3.65588000	2.87647600
O	6.50874800	-3.40932300	3.48140300
C	3.84432400	3.11435200	5.47404000
C	3.88341400	1.66032800	5.56158600
C	2.88586600	0.97801400	6.27009000
C	2.86608200	-0.43657200	6.35738700
C	3.91974400	-1.17651900	5.75366600
C	3.97928000	-2.63015900	5.90039300
C	4.79263700	0.91399400	4.76575300
C	4.82348400	-0.52213500	4.89383600
C	5.55753600	3.01301000	3.68248200
C	5.57510800	1.55542500	3.78553100
C	6.25690800	0.77392200	2.80984200
C	6.32250600	-0.63219400	2.98624600
C	5.69338100	-1.27388700	4.06092900
C	5.76460500	-2.72409700	4.18106900
C	4.98800200	-4.75508400	5.31731700
H	5.82175900	-5.13424200	4.73104200
H	4.04962500	-5.20193100	4.97853400
H	5.11194000	-4.98518700	6.37709800
H	4.34230100	5.46306300	3.49735200
C	6.71000700	1.16743400	1.51804900
C	7.05994000	0.10047200	0.72301100
S	7.02305400	-1.42429700	1.60475600
H	6.63930800	2.18852600	1.16788400
C	1.62093500	-0.91785400	6.86117500
C	0.71689200	0.08737500	7.10732100
S	1.42650900	1.68812800	6.90586800
H	1.38268000	-1.97353800	6.89604900
O	-6.52452800	3.40240500	3.49790800
N	-4.93849800	3.32277700	5.13487700
O	-6.22813000	-3.65732500	2.88670000
N	-4.69919300	-3.71214500	4.54874800
C	-4.98747700	4.77200000	5.31120900
H	-5.18676800	5.00239800	6.35950100
H	-5.77565600	5.15955400	4.67037800

O	-3.29037700	3.27922800	6.67885900
O	-3.06547400	-3.78576100	6.13898900
C	-5.76581700	2.73429400	4.18373300
C	-5.68672300	1.26732900	4.05376100
C	-6.30898600	0.63881300	2.98878300
C	-6.24563400	-0.78629100	2.80993300
C	-5.57330900	-1.54976800	3.77789000
C	-5.54546700	-3.02060600	3.67363100
C	-4.83656600	0.51403000	4.90623900
C	-4.80091700	-0.90455300	4.77330700
C	-3.99246600	2.64228200	5.90988600
C	-3.92218100	1.17574900	5.76094400
C	-2.87287000	0.45746500	6.35078400
C	-2.88168800	-0.97588100	6.24958200
C	-3.86672800	-1.64720600	5.54501400
C	-3.82439300	-3.11809400	5.45257700
C	-4.70322200	-5.16773400	4.42596100
H	-4.02924100	-5.56794300	5.17938900
H	-4.37224100	-5.45329600	3.42499300
H	-5.71898400	-5.53996300	4.57172900
H	-4.02337600	5.20774200	5.03999400
C	-1.63566400	0.94206000	6.88106300
C	-0.72177400	-0.05757300	7.09099500
S	-1.42102800	-1.67216100	6.90005400
H	-1.39636800	1.99577200	6.92866900
C	-6.72397400	-1.18010900	1.52320400
C	-7.04255300	-0.10923700	0.72552200
S	-7.02448100	1.42281900	1.60790100
H	-6.66506200	-2.19883900	1.16732400
O	-3.07567000	-3.79676900	-6.14957400
N	-4.73426500	-3.70532800	-4.57838700
O	-3.25403900	3.27695800	-6.65255200
N	-4.94084900	3.30788900	-5.14282900
C	-4.74806800	-5.15937500	-4.46605300
H	-4.34230100	-5.46306300	-3.49735200
H	-4.14049300	-5.56174700	-5.27327400
O	-6.22667700	-3.65588000	-2.87647600
O	-6.50874800	3.40932300	-3.48140300
C	-3.84432400	-3.11435200	-5.47404000
C	-3.88341400	-1.66032800	-5.56158600
C	-2.88586600	-0.97801400	-6.27009000
C	-2.86608200	0.43657200	-6.35738700
C	-3.91974400	1.17651900	-5.75366600
C	-3.97928000	2.63015900	-5.90039300
C	-4.79263700	-0.91399400	-4.76575300
C	-4.82348400	0.52213500	-4.89383600
C	-5.55753600	-3.01301000	-3.68248200
C	-5.57510800	-1.55542500	-3.78553100
C	-6.25690800	-0.77392200	-2.80984200
C	-6.32250600	0.63219400	-2.98624600
C	-5.69338100	1.27388700	-4.06092900
C	-5.76460500	2.72409700	-4.18106900
C	-4.98800200	4.75508400	-5.31731700
H	-4.04962500	5.20193100	-4.97853400
H	-5.82175900	5.13424200	-4.73104200
H	-5.11194000	4.98518700	-6.37709800
H	-5.77824300	-5.51396000	-4.53033900

C	-6.71000700	-1.16743400	-1.51804900
C	-7.05994000	-0.10047200	-0.72301100
S	-7.02305400	1.42429700	-1.60475600
H	-6.63930800	-2.18852600	-1.16788400
C	-1.62093500	0.91785400	-6.86117500
C	-0.71689200	-0.08737500	-7.10732100
S	-1.42650900	-1.68812800	-6.90586800
H	-1.38268000	1.97353800	-6.89604900
O	6.52452800	-3.40240500	-3.49790800
N	4.93849800	-3.32277700	-5.13487700
O	6.22813000	3.65732500	-2.88670000
N	4.69919300	3.71214500	-4.54874800
C	4.98747700	-4.77200000	-5.31120900
H	4.02337600	-5.20774200	-5.03999400
H	5.77565600	-5.15955400	-4.67037800
O	3.29037700	-3.27922800	-6.67885900
O	3.06547400	3.78576100	-6.13898900
C	5.76581700	-2.73429400	-4.18373300
C	5.68672300	-1.26732900	-4.05376100
C	6.30898600	-0.63881300	-2.98878300
C	6.24563400	0.78629100	-2.80993300
C	5.57330900	1.54976800	-3.77789000
C	5.54546700	3.02060600	-3.67363100
C	4.83656600	-0.51403000	-4.90623900
C	4.80091700	0.90455300	-4.77330700
C	3.99246600	-2.64228200	-5.90988600
C	3.92218100	-1.17574900	-5.76094400
C	2.87287000	-0.45746500	-6.35078400
C	2.88168800	0.97588100	-6.24958200
C	3.86672800	1.64720600	-5.54501400
C	3.82439300	3.11809400	-5.45257700
C	4.70322200	5.16773400	-4.42596100
H	4.02924100	5.56794300	-5.17938900
H	5.71898400	5.53996300	-4.57172900
H	4.37224100	5.45329600	-3.42499300
H	5.18676800	-5.00239800	-6.35950100
C	1.63566400	-0.94206000	-6.88106300
C	0.72177400	0.05757300	-7.09099500
S	1.42102800	1.67216100	-6.90005400
H	1.39636800	-1.99577200	-6.92866900
C	6.72397400	1.18010900	-1.52320400
C	7.04255300	0.10923700	-0.72552200
S	7.02448100	-1.42281900	-1.60790100
H	6.66506200	2.19883900	-1.16732400

ABAB (singlet)
M06-2X(D3)//6-31+G(d)
E = -7893.423537 a.u.

O	-4.29464300	6.16459200	3.46955400
N	-2.20511300	7.03430800	3.18287700
O	-4.69731200	4.76357200	-3.46285000
N	-2.63465000	5.64038500	-3.74861000
C	-2.10128800	7.37257400	4.60358700
H	-1.38539000	6.70730100	5.09172400
H	-3.08669400	7.25746800	5.04805800
O	-0.06076100	7.70316700	2.93670900
O	-0.47195000	6.31681100	-4.00273800

C	-3.37123500	6.43504800	2.72160300
C	-3.44382600	6.14413700	1.27326800
C	-4.48046100	5.37139800	0.78122300
C	-4.57427400	5.04262700	-0.61395100
C	-3.61639500	5.54915600	-1.49473900
C	-3.71773200	5.27363400	-2.94965300
C	-2.37060600	6.48870600	0.41319500
C	-2.47229900	6.20330500	-0.98083100
C	-1.06429500	7.25777800	2.41010100
C	-1.14949500	6.95009200	0.96065300
C	0.00024500	6.97270800	0.16520800
C	-0.12748500	6.71158600	-1.24251800
C	-1.34914300	6.41272200	-1.81795600
C	-1.42513300	6.12744500	-3.26729400
C	-2.77022400	5.38319100	-5.18342500
H	-1.91318500	5.82519400	-5.68536800
H	-3.70250400	5.82510600	-5.53591600
H	-2.80215100	4.30661000	-5.36631100
H	-1.74534500	8.39874800	4.69866900
C	-5.54915600	4.01574800	-0.84892300
C	-6.11177900	3.55803500	0.30641100
S	-5.66108400	4.50265700	1.72682900
H	-5.67314100	3.54307100	-1.81296100
C	1.37815300	7.00613500	0.57093600
C	2.22485500	6.69076900	-0.45147200
S	1.42389600	6.57660300	-2.01991900
H	1.68226700	7.12107300	1.60207200
O	6.16459200	4.29464300	-3.46955400
N	7.03430800	2.20511300	-3.18287700
O	4.76357200	4.69731200	3.46285000
N	5.64038500	2.63465000	3.74861000
C	7.37257400	2.10128800	-4.60358700
H	8.39874800	1.74534500	-4.69866900
H	7.25746800	3.08669400	-5.04805800
O	7.70316700	0.06076100	-2.93670900
O	6.31681100	0.47195000	4.00273800
C	6.43504800	3.37123500	-2.72160300
C	6.14413700	3.44382600	-1.27326800
C	5.37139800	4.48046100	-0.78122300
C	5.04262700	4.57427400	0.61395100
C	5.54915600	3.61639500	1.49473900
C	5.27363400	3.71773200	2.94965300
C	6.48870600	2.37060600	-0.41319500
C	6.20330500	2.47229900	0.98083100
C	7.25777800	1.06429500	-2.41010100
C	6.95009200	1.14949500	-0.96065300
C	6.97270800	-0.00024500	-0.16520800
C	6.71158600	0.12748500	1.24251800
C	6.41272200	1.34914300	1.81795600
C	6.12744500	1.42513300	3.26729400
C	5.38319100	2.77022400	5.18342500
H	4.30661000	2.80215100	5.36631100
H	5.82510600	3.70250400	5.53591600
H	5.82519400	1.91318500	5.68536800
H	6.70730100	1.38539000	-5.09172400
C	4.01574800	5.54915600	0.84892300
C	3.55803500	6.11177900	-0.30641100

S	4.50265700	5.66108400	-1.72682900
H	3.54307100	5.67314100	1.81296100
C	7.00613500	-1.37815300	-0.57093600
C	6.69076900	-2.22485500	0.45147200
S	6.57660300	-1.42389600	2.01991900
H	7.12107300	-1.68226700	-1.60207200
O	4.29464300	-6.16459200	3.46955400
N	2.20511300	-7.03430800	3.18287700
O	4.69731200	-4.76357200	-3.46285000
N	2.63465000	-5.64038500	-3.74861000
C	2.10128800	-7.37257400	4.60358700
H	1.38539000	-6.70730100	5.09172400
H	3.08669400	-7.25746800	5.04805800
O	0.06076100	-7.70316700	2.93670900
O	0.47195000	-6.31681100	-4.00273800
C	3.37123500	-6.43504800	2.72160300
C	3.44382600	-6.14413700	1.27326800
C	4.48046100	-5.37139800	0.78122300
C	4.57427400	-5.04262700	-0.61395100
C	3.61639500	-5.54915600	-1.49473900
C	3.71773200	-5.27363400	-2.94965300
C	2.37060600	-6.48870600	0.41319500
C	2.47229900	-6.20330500	-0.98083100
C	1.06429500	-7.25777800	2.41010100
C	1.14949500	-6.95009200	0.96065300
C	-0.00024500	-6.97270800	0.16520800
C	0.12748500	-6.71158600	-1.24251800
C	1.34914300	-6.41272200	-1.81795600
C	1.42513300	-6.12744500	-3.26729400
C	2.77022400	-5.38319100	-5.18342500
H	1.91318500	-5.82519400	-5.68536800
H	3.70250400	-5.82510600	-5.53591600
H	2.80215100	-4.30661000	-5.36631100
H	1.74534500	-8.39874800	4.69866900
C	5.54915600	-4.01574800	-0.84892300
C	6.11177900	-3.55803500	0.30641100
S	5.66108400	-4.50265700	1.72682900
H	5.67314100	-3.54307100	-1.81296100
C	-1.37815300	-7.00613500	0.57093600
C	-2.22485500	-6.69076900	-0.45147200
S	-1.42389600	-6.57660300	-2.01991900
H	-1.68226700	-7.12107300	1.60207200
O	-6.16459200	-4.29464300	-3.46955400
N	-7.03430800	-2.20511300	-3.18287700
O	-4.76357200	-4.69731200	3.46285000
N	-5.64038500	-2.63465000	3.74861000
C	-7.37257400	-2.10128800	-4.60358700
H	-8.39874800	-1.74534500	-4.69866900
H	-7.25746800	-3.08669400	-5.04805800
O	-7.70316700	-0.06076100	-2.93670900
O	-6.31681100	-0.47195000	4.00273800
C	-6.43504800	-3.37123500	-2.72160300
C	-6.14413700	-3.44382600	-1.27326800
C	-5.37139800	-4.48046100	-0.78122300
C	-5.04262700	-4.57427400	0.61395100
C	-5.54915600	-3.61639500	1.49473900
C	-5.27363400	-3.71773200	2.94965300

C	-6.48870600	-2.37060600	-0.41319500
C	-6.20330500	-2.47229900	0.98083100
C	-7.25777800	-1.06429500	-2.41010100
C	-6.95009200	-1.14949500	-0.96065300
C	-6.97270800	0.00024500	-0.16520800
C	-6.71158600	-0.12748500	1.24251800
C	-6.41272200	-1.34914300	1.81795600
C	-6.12744500	-1.42513300	3.26729400
C	-5.38319100	-2.77022400	5.18342500
H	-5.82519400	-1.91318500	5.68536800
H	-4.30661000	-2.80215100	5.36631100
H	-5.82510600	-3.70250400	5.53591600
H	-6.70730100	-1.38539000	-5.09172400
C	-4.01574800	-5.54915600	0.84892300
C	-3.55803500	-6.11177900	-0.30641100
S	-4.50265700	-5.66108400	-1.72682900
H	-3.54307100	-5.67314100	1.81296100
C	-7.00613500	1.37815300	-0.57093600
C	-6.69076900	2.22485500	0.45147200
S	-6.57660300	1.42389600	2.01991900
H	-7.12107300	1.68226700	-1.60207200

ABAB$^{-\bullet}$
(U)M06-2X(D3)//6-31+G(d)
E = -7893.553627 a.u.

O	-2.84521400	-6.39189200	3.77743700
N	-4.65132900	-5.02571400	3.49652100
O	-2.12648100	-6.87399900	-3.25953600
N	-3.94281200	-5.55354200	-3.51542500
C	-4.85086400	-4.92450500	4.94101300
H	-4.60248200	-3.91601300	5.28024800
H	-4.20298100	-5.65464800	5.41974400
O	-6.31691400	-3.51944300	3.24179600
O	-5.64774700	-4.06312400	-3.79557800
C	-3.60408400	-5.80646900	3.01909500
C	-3.46083200	-5.89644100	1.55634500
C	-2.32756200	-6.48161900	1.01254400
C	-2.13103400	-6.57080700	-0.40534200
C	-3.14043300	-6.08554000	-1.25706400
C	-3.00158200	-6.21436300	-2.72063100
C	-4.37170900	-5.22456300	0.69945500
C	-4.21325600	-5.34333500	-0.71829500
C	-5.48707500	-4.23488800	2.70249500
C	-5.32224700	-4.33167900	1.23923100
C	-5.99867500	-3.43622000	0.39035900
C	-5.86095700	-3.59821900	-1.02797400
C	-5.05183000	-4.58258500	-1.57455500
C	-4.92450500	-4.69222400	-3.03741300
C	-3.79357600	-5.71590000	-4.96039600
H	-4.64393800	-5.23753100	-5.44010100
H	-3.75480800	-6.77986400	-5.19729900
H	-2.86103700	-5.25286700	-5.29197500
H	-5.89945300	-5.11813600	5.17080200
C	-0.79675700	-6.95728700	-0.72957600
C	0.00353300	-7.07801100	0.37880000
S	-0.90193500	-6.95917300	1.88993200
H	-0.43133100	-6.98358800	-1.74654100

C	-6.65413000	-2.21300100	0.72031700
C	-6.92821300	-1.44503300	-0.38367500
S	-6.61388800	-2.29323900	-1.89983900
H	-6.76458200	-1.87041400	1.73959600
O	-6.39189200	2.84521400	-3.77743700
N	-5.02571400	4.65132900	-3.49652100
O	-6.87399900	2.12648100	3.25953600
N	-5.55354200	3.94281200	3.51542500
C	-4.92450500	4.85086400	-4.94101300
H	-5.11813600	5.89945300	-5.17080200
H	-5.65464800	4.20298100	-5.41974400
O	-3.51944300	6.31691400	-3.24179600
O	-4.06312400	5.64774700	3.79557800
C	-5.80646900	3.60408400	-3.01909500
C	-5.89644100	3.46083200	-1.55634500
C	-6.48161900	2.32756200	-1.01254400
C	-6.57080700	2.13103400	0.40534200
C	-6.08554000	3.14043300	1.25706400
C	-6.21436300	3.00158200	2.72063100
C	-5.22456300	4.37170900	-0.69945500
C	-5.34333500	4.21325600	0.71829500
C	-4.23488800	5.48707500	-2.70249500
C	-4.33167900	5.32224700	-1.23923100
C	-3.43622000	5.99867500	-0.39035900
C	-3.59821900	5.86095700	1.02797400
C	-4.58258500	5.05183000	1.57455500
C	-4.69222400	4.92450500	3.03741300
C	-5.71590000	3.79357600	4.96039600
H	-5.25286700	2.86103700	5.29197500
H	-6.77986400	3.75480800	5.19729900
H	-5.23753100	4.64393800	5.44010100
H	-3.91601300	4.60248200	-5.28024800
C	-6.95728700	0.79675700	0.72957600
C	-7.07801100	-0.00353300	-0.37880000
S	-6.95917300	0.90193500	-1.88993200
H	-6.98358800	0.43133100	1.74654100
C	-2.21300100	6.65413000	-0.72031700
C	-1.44503300	6.92821300	0.38367500
S	-2.29323900	6.61388800	1.89983900
H	-1.87041400	6.76458200	-1.73959600
O	2.84521400	6.39189200	3.77743700
N	4.65132900	5.02571400	3.49652100
O	2.12648100	6.87399900	-3.25953600
N	3.94281200	5.55354200	-3.51542500
C	4.85086400	4.92450500	4.94101300
H	4.60248200	3.91601300	5.28024800
H	4.20298100	5.65464800	5.41974400
O	6.31691400	3.51944300	3.24179600
O	5.64774700	4.06312400	-3.79557800
C	3.60408400	5.80646900	3.01909500
C	3.46083200	5.89644100	1.55634500
C	2.32756200	6.48161900	1.01254400
C	2.13103400	6.57080700	-0.40534200
C	3.14043300	6.08554000	-1.25706400
C	3.00158200	6.21436300	-2.72063100
C	4.37170900	5.22456300	0.69945500
C	4.21325600	5.34333500	-0.71829500

C	5.48707500	4.23488800	2.70249500
C	5.32224700	4.33167900	1.23923100
C	5.99867500	3.43622000	0.39035900
C	5.86095700	3.59821900	-1.02797400
C	5.05183000	4.58258500	-1.57455500
C	4.92450500	4.69222400	-3.03741300
C	3.79357600	5.71590000	-4.96039600
H	4.64393800	5.23753100	-5.44010100
H	3.75480800	6.77986400	-5.19729900
H	2.86103700	5.25286700	-5.29197500
H	5.89945300	5.11813600	5.17080200
C	0.79675700	6.95728700	-0.72957600
C	-0.00353300	7.07801100	0.37880000
S	0.90193500	6.95917300	1.88993200
H	0.43133100	6.98358800	-1.74654100
C	6.65413000	2.21300100	0.72031700
C	6.92821300	1.44503300	-0.38367500
S	6.61388800	2.29323900	-1.89983900
H	6.76458200	1.87041400	1.73959600
O	6.39189200	-2.84521400	-3.77743700
N	5.02571400	-4.65132900	-3.49652100
O	6.87399900	-2.12648100	3.25953600
N	5.55354200	-3.94281200	3.51542500
C	4.92450500	-4.85086400	-4.94101300
H	5.11813600	-5.89945300	-5.17080200
H	5.65464800	-4.20298100	-5.41974400
O	3.51944300	-6.31691400	-3.24179600
O	4.06312400	-5.64774700	3.79557800
C	5.80646900	-3.60408400	-3.01909500
C	5.89644100	-3.46083200	-1.55634500
C	6.48161900	-2.32756200	-1.01254400
C	6.57080700	-2.13103400	0.40534200
C	6.08554000	-3.14043300	1.25706400
C	6.21436300	-3.00158200	2.72063100
C	5.22456300	-4.37170900	-0.69945500
C	5.34333500	-4.21325600	0.71829500
C	4.23488800	-5.48707500	-2.70249500
C	4.33167900	-5.32224700	-1.23923100
C	3.43622000	-5.99867500	-0.39035900
C	3.59821900	-5.86095700	1.02797400
C	4.58258500	-5.05183000	1.57455500
C	4.69222400	-4.92450500	3.03741300
C	5.71590000	-3.79357600	4.96039600
H	5.23753100	-4.64393800	5.44010100
H	5.25286700	-2.86103700	5.29197500
H	6.77986400	-3.75480800	5.19729900
H	3.91601300	-4.60248200	-5.28024800
C	6.95728700	-0.79675700	0.72957600
C	7.07801100	0.00353300	-0.37880000
S	6.95917300	-0.90193500	-1.88993200
H	6.98358800	-0.43133100	1.74654100
C	2.21300100	-6.65413000	-0.72031700
C	1.44503300	-6.92821300	0.38367500
S	2.29323900	-6.61388800	1.89983900
H	1.87041400	-6.76458200	-1.73959600

ABAB$^{2-\bullet\bullet}$(OSS)

(U)M06-2X(D3)//6-31+G(d)
E = -7893.622479 a.u.

O	-6.33420400	-3.00698500	3.79067600
N	-6.78931200	-0.78914500	3.50987900
O	-6.16587000	-3.83857000	-3.25069200
N	-6.66051600	-1.64773300	-3.49657200
C	-6.86169300	-0.58036700	4.95347800
H	-6.03753100	0.06030000	5.27586400
H	-6.79775400	-1.55313700	5.43493700
O	-7.09696100	1.43562700	3.26317700
O	-7.01558600	0.58823100	-3.77776300
C	-6.51581300	-2.06622000	3.03088100
C	-6.46689300	-2.22540800	1.56786600
C	-5.99230200	-3.40695400	1.02162000
C	-5.91153600	-3.60459600	-0.39881400
C	-6.37478100	-2.57760700	-1.24307000
C	-6.37722400	-2.76651600	-2.70376500
C	-6.72794500	-1.11926600	0.71420100
C	-6.69827900	-1.31340300	-0.70096000
C	-6.92260300	0.35682400	2.71647700
C	-6.86475600	0.17878800	1.25557900
C	-6.79738000	1.30184000	0.40926800
C	-6.81437400	1.08655300	-1.01079300
C	-6.84842400	-0.18713700	-1.55505000
C	-6.84944800	-0.35533300	-3.01789200
C	-6.66578100	-1.87048400	-4.93988100
H	-6.95151600	-0.93787100	-5.42027300
H	-7.37156900	-2.66799000	-5.17857300
H	-5.67146100	-2.18090500	-5.26943700
H	-7.80099700	-0.08153700	5.19847600
C	-5.15892000	-4.76782100	-0.72964400
C	-4.60846300	-5.37783300	0.37341500
S	-5.21175900	-4.69904100	1.89031900
H	-4.90912000	-5.02834500	-1.74838100
C	-6.49738500	2.65535000	0.73614800
C	-6.20429400	3.41839500	-0.37007400
S	-6.54302300	2.56924300	-1.88347500
H	-6.34573700	2.98766800	1.75333700
O	-3.01471300	6.32893600	-3.79198100
N	-0.79711700	6.80063900	-3.50168400
O	-3.83207200	6.14049000	3.25354300
N	-1.64542800	6.67420700	3.48823200
C	-0.59186300	6.87496500	-4.94397100
H	-0.05445400	7.79435700	-5.18289200
H	-1.56876700	6.85366400	-5.42119700
O	1.43753700	7.06926100	-3.26585500
O	0.59627600	7.01259200	3.77907100
C	-2.07553900	6.52067200	-3.02311000
C	-2.23000000	6.47479600	-1.57256100
C	-3.41932100	5.99622200	-1.01602800
C	-3.60936500	5.90972600	0.39106000
C	-2.57218600	6.37079900	1.24798700
C	-2.76124500	6.37140400	2.69888800
C	-1.11804800	6.71258600	-0.72054900
C	-1.31030800	6.68343800	0.70739200
C	0.35293500	6.91426300	-2.71158000
C	0.17410000	6.85811500	-1.26046000

C	1.30585400	6.79609500	-0.40150700
C	1.09569700	6.82113100	1.00511400
C	-0.18669100	6.85692900	1.55962900
C	-0.34940100	6.85681400	3.01002600
C	-1.86552000	6.68289400	4.93021400
H	-2.13519200	5.68027300	5.27224000
H	-2.69198100	7.35666200	5.16291600
H	-0.94394000	7.00995700	5.40591100
H	0.01350000	6.02842500	-5.27840900
C	-4.75903700	5.14217300	0.72366000
C	-5.39079300	4.61235900	-0.38005800
S	-4.70518000	5.21379600	-1.88905600
H	-5.00689700	4.88567000	1.74470800
C	2.65189900	6.47704300	-0.72995500
C	3.42790800	6.21077300	0.37675800
S	2.57309100	6.54534500	1.88221700
H	2.97482200	6.31470200	-1.74933700
O	6.33420400	3.00698600	3.79067600
N	6.78931200	0.78914500	3.50987900
O	6.16587000	3.83857000	-3.25069200
N	6.66051600	1.64773300	-3.49657200
C	6.86169400	0.58036700	4.95347800
H	6.03753100	-0.06030000	5.27586400
H	6.79775400	1.55313700	5.43493700
O	7.09696100	-1.43562700	3.26317700
O	7.01558600	-0.58823100	-3.77776300
C	6.51581300	2.06622000	3.03088100
C	6.46689400	2.22540800	1.56786600
C	5.99230200	3.40695400	1.02162000
C	5.91153600	3.60459600	-0.39881400
C	6.37478100	2.57760700	-1.24307000
C	6.37722300	2.76651600	-2.70376500
C	6.72794500	1.11926600	0.71420100
C	6.69827900	1.31340300	-0.70096000
C	6.92260300	-0.35682400	2.71647700
C	6.86475600	-0.17878800	1.25557900
C	6.79738000	-1.30184000	0.40926800
C	6.81437400	-1.08655300	-1.01079300
C	6.84842400	0.18713700	-1.55505000
C	6.84944800	0.35533300	-3.01789200
C	6.66578100	1.87048400	-4.93988100
H	6.95151600	0.93787100	-5.42027300
H	7.37156900	2.66799000	-5.17857300
H	5.67146000	2.18090400	-5.26943700
H	7.80099700	0.08153700	5.19847600
C	5.15892000	4.76782100	-0.72964400
C	4.60846300	5.37783300	0.37341500
S	5.21175900	4.69904100	1.89031900
H	4.90912000	5.02834500	-1.74838100
C	6.49738500	-2.65535000	0.73614800
C	6.20429400	-3.41839500	-0.37007400
S	6.54302300	-2.56924300	-1.88347500
H	6.34573700	-2.98766800	1.75333700
O	3.01471300	-6.32893600	-3.79198100
N	0.79711700	-6.80063900	-3.50168400
O	3.83207200	-6.14049100	3.25354300
N	1.64542800	-6.67420700	3.48823200

C	0.59186300	-6.87496500	-4.94397100
H	0.05445400	-7.79435700	-5.18289200
H	1.56876700	-6.85366400	-5.42119700
O	-1.43753700	-7.06926100	-3.26585500
O	-0.59627600	-7.01259200	3.77907100
C	2.07553900	-6.52067200	-3.02311000
C	2.23000000	-6.47479600	-1.57256100
C	3.41932200	-5.99622200	-1.01602700
C	3.60936500	-5.90972600	0.39106000
C	2.57218600	-6.37079900	1.24798700
C	2.76124500	-6.37140500	2.69888800
C	1.11804800	-6.71258600	-0.72054900
C	1.31030800	-6.68343800	0.70739200
C	-0.35293500	-6.91426300	-2.71158000
C	-0.17410000	-6.85811500	-1.26046000
C	-1.30585400	-6.79609500	-0.40150700
C	-1.09569700	-6.82113100	1.00511400
C	0.18669100	-6.85692900	1.55962900
C	0.34940100	-6.85681400	3.01002600
C	1.86552000	-6.68289400	4.93021400
H	0.94394000	-7.00995700	5.40591100
H	2.13519200	-5.68027300	5.27224000
H	2.69198000	-7.35666200	5.16291600
H	-0.01350000	-6.02842500	-5.27840900
C	4.75903700	-5.14217300	0.72366000
C	5.39079300	-4.61235900	-0.38005800
S	4.70518000	-5.21379600	-1.88905600
H	5.00689700	-4.88567000	1.74470800
C	-2.65189800	-6.47704300	-0.72995500
C	-3.42790800	-6.21077300	0.37675800
S	-2.57309100	-6.54534500	1.88221700
H	-2.97482200	-6.31470200	-1.74933700

ABAB$^{2-\bullet\bullet}$ (triplet)
(U)M06-2X(D3)//6-31+G(d)
E = -7893.619519 a.u.

O	-1.82280800	6.78884100	3.78210600
N	0.44000500	6.83977700	3.50059700
O	-2.68358300	6.74985500	-3.25675700
N	-0.44006200	6.82567700	-3.50786600
C	0.66045100	6.88087700	4.94400500
H	1.17227700	5.97054700	5.26407100
H	-0.30994300	6.96292900	5.42741400
O	2.68358300	6.76409000	3.24978200
O	1.82275400	6.77437700	-3.78918200
C	-0.86560400	6.79607200	3.02283900
C	-1.03375600	6.76970900	1.55795800
C	-2.28081400	6.50993000	1.01546400
C	-2.49184500	6.46210300	-0.40564200
C	-1.40333500	6.73096500	-1.25023600
C	-1.59054700	6.76049500	-2.71296800
C	0.09867400	6.83172000	0.70238000
C	-0.09874300	6.82883700	-0.70962000
C	1.59054700	6.77222600	2.70593600
C	1.40334900	6.73669300	1.24337800
C	2.49198000	6.46485300	0.39987000
C	2.28087400	6.50680500	-1.02144700

C	1.03371600	6.76386700	-1.56496100
C	0.86556000	6.78420000	-3.02993400
C	-0.66047800	6.86110200	-4.95142900
H	0.30979600	6.94334400	-5.43505500
H	-1.29533700	7.71382100	-5.19907100
H	-1.17040300	5.94858100	-5.26831700
H	1.29358400	7.73577900	5.18858500
C	-3.77499300	5.92698700	-0.73322200
C	-4.46607200	5.49433100	0.37056600
S	-3.68991700	5.97054000	1.88657100
H	-4.07750100	5.72337900	-1.75052100
C	3.77551500	5.93199700	0.72957600
C	4.46670100	5.49500400	-0.37246800
S	3.69003600	5.96434100	-1.89045200
H	4.07852000	5.73339600	1.74772700
O	6.78822800	1.83410700	-3.78394300
N	6.86221200	-0.43216700	-3.48906800
O	6.71326600	2.68103800	3.26074200
N	6.84722000	0.43341600	3.49651600
C	6.90959900	-0.64762000	-4.93070300
H	7.71480700	-1.34704100	-5.16216000
H	7.07439300	0.31636500	-5.40633300
O	6.72734200	-2.67979300	-3.25389600
O	6.77194800	-1.83285500	3.79105100
C	6.80868900	0.87670800	-3.01202200
C	6.78378100	1.03785500	-1.56418200
C	6.52071400	2.29654700	-1.00777900
C	6.46164900	2.49822200	0.39493000
C	6.72544400	1.39680900	1.25719000
C	6.75260100	1.58479500	2.70639000
C	6.81254400	-0.09665000	-0.71106800
C	6.80948200	0.09785600	0.71831000
C	6.76427400	-1.58355800	-2.69938100
C	6.73090600	-1.39560200	-1.25028200
C	6.46360100	-2.49706800	-0.38915300
C	6.51667200	-2.29535700	1.01377300
C	6.77715000	-1.03659200	1.57129000
C	6.79578500	-0.87543500	3.01924300
C	6.88826200	0.64888600	4.93834300
H	5.94555500	1.08642500	5.27760800
H	7.69272600	1.34795800	5.17340700
H	7.05052000	-0.31516200	5.41471200
H	5.96823100	-1.08474400	-5.27420900
C	5.90205900	3.76603200	0.72508500
C	5.50262200	4.48145800	-0.37965600
S	5.97724200	3.69815700	-1.88478300
H	5.68532300	4.05252300	1.74541800
C	5.90579900	-3.76512600	-0.72164500
C	5.50170300	-4.48043400	0.38143200
S	5.96968500	-3.69690200	1.88855100
H	5.69409900	-4.05201900	-1.74292600
O	1.82280800	-6.78884100	3.78210600
N	-0.44000500	-6.83977700	3.50059700
O	2.68358300	-6.74985500	-3.25675700
N	0.44006200	-6.82567700	-3.50786600
C	-0.66045100	-6.88087700	4.94400500
H	-1.17227700	-5.97054700	5.26407100

H	0.30994300	-6.96292900	5.42741400
O	-2.68358300	-6.76409000	3.24978200
O	-1.82275400	-6.77437700	-3.78918200
C	0.86560400	-6.79607200	3.02283900
C	1.03375600	-6.76970900	1.55795800
C	2.28081400	-6.50993000	1.01546400
C	2.49184500	-6.46210300	-0.40564200
C	1.40333500	-6.73096500	-1.25023600
C	1.59054700	-6.76049500	-2.71296800
C	-0.09867400	-6.83172000	0.70238000
C	0.09874300	-6.82883700	-0.70962000
C	-1.59054700	-6.77222600	2.70593600
C	-1.40334900	-6.73669300	1.24337800
C	-2.49198000	-6.46485300	0.39987000
C	-2.28087400	-6.50680500	-1.02144700
C	-1.03371600	-6.76386700	-1.56496100
C	-0.86556000	-6.78420000	-3.02993400
C	0.66047800	-6.86110200	-4.95142900
H	-0.30979600	-6.94334400	-5.43505500
H	1.29533700	-7.71382100	-5.19907100
H	1.17040300	-5.94858100	-5.26831700
H	-1.29358400	-7.73577900	5.18858500
C	3.77499300	-5.92698700	-0.73322200
C	4.46607200	-5.49433100	0.37056600
S	3.68991700	-5.97054000	1.88657100
H	4.07750100	-5.72337900	-1.75052100
C	-3.77551500	-5.93199700	0.72957600
C	-4.46670100	-5.49500400	-0.37246800
S	-3.69003600	-5.96434100	-1.89045200
H	-4.07852000	-5.73339600	1.74772700
O	-6.78822800	-1.83410700	-3.78394300
N	-6.86221200	0.43216700	-3.48906800
O	-6.71326600	-2.68103800	3.26074200
N	-6.84722000	-0.43341600	3.49651600
C	-6.90959900	0.64762000	-4.93070300
H	-7.71480700	1.34704100	-5.16216000
H	-7.07439300	-0.31636500	-5.40633300
O	-6.72734200	2.67979300	-3.25389600
O	-6.77194800	1.83285500	3.79105100
C	-6.80868900	-0.87670800	-3.01202200
C	-6.78378100	-1.03785500	-1.56418200
C	-6.52071400	-2.29654700	-1.00777900
C	-6.46164900	-2.49822200	0.39493000
C	-6.72544400	-1.39680900	1.25719000
C	-6.75260100	-1.58479500	2.70639000
C	-6.81254400	0.09665000	-0.71106800
C	-6.80948200	-0.09785600	0.71831000
C	-6.76427400	1.58355800	-2.69938100
C	-6.73090600	1.39560200	-1.25028200
C	-6.46360100	2.49706800	-0.38915300
C	-6.51667200	2.29535700	1.01377300
C	-6.77715000	1.03659200	1.57129000
C	-6.79578500	0.87543500	3.01924300
C	-6.88826200	-0.64888600	4.93834300
H	-7.05052000	0.31516200	5.41471200
H	-5.94555500	-1.08642500	5.27760800
H	-7.69272600	-1.34795800	5.17340700

H	-5.96823100	1.08474400	-5.27420900
C	-5.90205900	-3.76603200	0.72508500
C	-5.50262200	-4.48145800	-0.37965600
S	-5.97724200	-3.69815700	-1.88478300
H	-5.68532300	-4.05252300	1.74541800
C	-5.90579900	3.76512600	-0.72164500
C	-5.50170300	4.48043400	0.38143200
S	-5.96968500	3.69690200	1.88855100
H	-5.69409900	4.05201900	-1.74292600

A_3B (singlet)
M06-2X(D3)//6-31+G(d)
E = -7893.429678 a.u.

O	-3.93501300	1.45937200	6.69737800
N	-3.45762200	-0.76765400	6.81725500
O	2.98706300	2.97552400	6.72223700
N	3.45762200	0.76765400	6.81725500
C	-4.87876000	-1.11672300	6.86150700
H	-5.44329100	-0.20209100	7.02441900
H	-5.04397800	-1.82914000	7.67021500
O	-2.98706300	-2.97552400	6.72223700
O	3.93501300	-1.45937200	6.69737800
C	-3.09960400	0.57351400	6.74955500
C	-1.65244500	0.87897200	6.75053300
C	-1.22497800	2.16892500	6.49202300
C	0.17108400	2.50780000	6.46128500
C	1.11329000	1.51027200	6.72676700
C	2.56128300	1.83487700	6.74825900
C	-0.69463100	-0.16253400	6.82811200
C	0.69463100	0.16253400	6.82811200
C	-2.56128300	-1.83487700	6.74825900
C	-1.11329000	-1.51027200	6.72676700
C	-0.17108400	-2.50780000	6.46128500
C	1.22497800	-2.16892500	6.49202300
C	1.65244500	-0.87897200	6.75053300
C	3.09960400	-0.57351400	6.74955500
C	4.87876000	1.11672300	6.86150700
H	5.17561800	1.58258100	5.91908400
H	5.44329100	0.20209100	7.02441900
H	5.04397800	1.82914000	7.67021500
H	-5.17561800	-1.58258100	5.91908400
C	0.38275400	3.82191200	5.92418700
C	-0.77646700	4.39943500	5.49382700
S	-2.22028600	3.48625200	5.93863100
H	1.36972900	4.22279100	5.74104600
C	-0.38275400	-3.82191200	5.92418700
C	0.77646700	-4.39943500	5.49382700
S	2.22028600	-3.48625200	5.93863100
H	-1.36972900	-4.22279100	5.74104600
O	4.42006800	-6.48925900	1.94704100
N	4.23487900	-6.59808300	-0.32386600
O	-2.63231400	-6.79444000	2.56775300
N	-2.81081100	-6.93772800	0.31978700
C	5.68925400	-6.57284100	-0.49126000
H	5.98371300	-7.37865000	-1.16438800
H	6.14126700	-6.69836300	0.48939200
O	4.04233100	-6.51320700	-2.57356100

O	-3.00632500	-6.86217800	-1.95147600
C	3.70730700	-6.56483000	0.96157500
C	2.23415800	-6.63038900	1.08111800
C	1.63572700	-6.39831100	2.30658800
C	0.20798700	-6.42229600	2.46220300
C	-0.58785800	-6.72654000	1.35372400
C	-2.06295200	-6.81258400	1.49133800
C	1.42030000	-6.74484700	-0.07265400
C	0.00299500	-6.81100100	0.07156600
C	3.47850500	-6.56462400	-1.49576700
C	2.00110600	-6.61471800	-1.35691000
C	1.18147400	-6.39648800	-2.46513000
C	-0.24270200	-6.50872400	-2.31221300
C	-0.81691000	-6.78754300	-1.08538200
C	-2.28863900	-6.86249800	-0.96627700
C	-4.26088400	-7.05180400	0.48578300
H	-4.47461900	-7.85446000	1.19202200
H	-4.69394800	-7.26240600	-0.48886500
H	-4.66349900	-6.11812700	0.88530800
H	5.99415200	-5.62083900	-0.93140500
C	-0.19143600	-5.89197400	3.73431200
C	0.86728200	-5.42843200	4.46079900
S	2.43129900	-5.82318900	3.74569300
H	-1.22778500	-5.73046000	3.99515000
C	1.52959700	-5.83906800	-3.74353400
C	0.43226100	-5.49108400	-4.47490300
S	-1.08923400	-6.03540700	-3.76126500
H	2.54651100	-5.57806100	-4.00457000
O	3.79424500	-1.79627000	-6.71426400
N	3.51064400	0.46554600	-6.79695300
O	-3.23205500	-2.70488000	-6.68044700
N	-3.51064400	-0.46554600	-6.79695300
C	4.95640300	0.69236200	-6.84095700
H	5.18248900	1.38768000	-7.64996100
H	5.44098600	-0.26704000	-7.00361500
O	3.23205500	2.70488000	-6.68044700
O	-3.79424500	1.79627000	-6.71426400
C	3.03816900	-0.84097100	-6.74807700
C	1.57027600	-1.02060400	-6.74225700
C	1.03442200	-2.27054600	-6.48963000
C	-0.38554800	-2.48122100	-6.44033800
C	-1.23904900	-1.40697800	-6.69849400
C	-2.71012300	-1.60549300	-6.71618600
C	0.70563600	0.10068500	-6.80749900
C	-0.70563600	-0.10068500	-6.80749900
C	2.71012300	1.60549300	-6.71618600
C	1.23904900	1.40697800	-6.69849400
C	0.38554800	2.48122100	-6.44033800
C	-1.03442200	2.27054600	-6.48963000
C	-1.57027600	1.02060400	-6.74225700
C	-3.03816900	0.84097100	-6.74807700
C	-4.95640300	-0.69236200	-6.84095700
H	-5.44098600	0.26704000	-7.00361500
H	-5.29079500	-1.13212900	-5.89866500
H	-5.18248900	-1.38768000	-7.64996100
H	5.29079500	1.13212900	-5.89866500
C	-0.70563600	-3.77359500	-5.89808100

C	0.40454400	-4.46090300	-5.50345200
S	1.91771800	-3.68431300	-5.97932000
H	-1.72147000	-4.07674200	-5.68220800
C	0.70563600	3.77359500	-5.89808100
C	-0.40454400	4.46090300	-5.50345200
S	-1.91771800	3.68431300	-5.97932000
H	1.72147000	4.07674200	-5.68220800
O	3.00632500	6.86217800	-1.95147600
N	2.81081100	6.93772800	0.31978700
O	-4.04233100	6.51320700	-2.57356100
N	-4.23487900	6.59808300	-0.32386600
C	4.26088400	7.05180400	0.48578300
H	4.47461900	7.85446000	1.19202200
H	4.69394800	7.26240600	-0.48886500
O	2.63231400	6.79444000	2.56775300
O	-4.42006800	6.48925900	1.94704100
C	2.28863900	6.86249800	-0.96627700
C	0.81691000	6.78754300	-1.08538200
C	0.24270200	6.50872400	-2.31221300
C	-1.18147400	6.39648800	-2.46513000
C	-2.00110600	6.61471800	-1.35691000
C	-3.47850500	6.56462400	-1.49576700
C	-0.00299500	6.81100100	0.07156600
C	-1.42030000	6.74484700	-0.07265400
C	2.06295200	6.81258400	1.49133800
C	0.58785800	6.72654000	1.35372400
C	-0.20798700	6.42229600	2.46220300
C	-1.63572700	6.39831100	2.30658800
C	-2.23415800	6.63038900	1.08111800
C	-3.70730700	6.56483000	0.96157500
C	-5.68925400	6.57284100	-0.49126000
H	-6.14126700	6.69836300	0.48939200
H	-5.99415200	5.62083900	-0.93140500
H	-5.98371300	7.37865000	-1.16438800
H	4.66349900	6.11812700	0.88530800
C	-1.52959700	5.83906800	-3.74353400
C	-0.43226100	5.49108400	-4.47490300
S	1.08923400	6.03540700	-3.76126500
H	-2.54651100	5.57806100	-4.00457000
C	0.19143600	5.89197400	3.73431200
C	-0.86728200	5.42843200	4.46079900
S	-2.43129900	5.82318900	3.74569300
H	1.22778500	5.73046000	3.99515000

$A_3B^{-\bullet}$
(U)M06-2X(D3)//6-31+G(d)
E = -7893.559488 a.u.

O	3.70742100	-1.98035700	6.73426300
N	3.51965400	0.29261700	6.82144400
O	-3.36097500	-2.54422100	6.69756900
N	-3.51965400	-0.29261700	6.82144400
C	4.97189800	0.44987600	6.86172300
H	5.40974000	-0.53140200	7.02763600
H	5.23361300	1.13936800	7.66539700
O	3.36097500	2.54422100	6.69756900
O	-3.70742100	1.98035700	6.73426300
C	2.98798800	-0.99153100	6.76617600

C	1.52155500	-1.09725000	6.75435300
C	0.92298100	-2.32318500	6.49796800
C	-0.50112300	-2.47240500	6.45110800
C	-1.30650900	-1.34510200	6.71458400
C	-2.77441400	-1.47258600	6.73558700
C	0.71353000	0.06920100	6.80792900
C	-0.71353000	-0.06920100	6.80792900
C	2.77441400	1.47258600	6.73558700
C	1.30650900	1.34510200	6.71458400
C	0.50112300	2.47240500	6.45110800
C	-0.92298100	2.32318500	6.49796800
C	-1.52155500	1.09725000	6.75435300
C	-2.98798800	0.99153100	6.76617600
C	-4.97189800	-0.44987600	6.86172300
H	-5.32700800	-0.86904900	5.91708600
H	-5.40974000	0.53140200	7.02763600
H	-5.23361300	-1.13936800	7.66539700
H	5.32700800	0.86904900	5.91708600
C	-0.88200900	-3.73607600	5.91550200
C	0.19431300	-4.48270800	5.49970000
S	1.73883000	-3.76462900	5.96442700
H	-1.91268600	-3.99691500	5.71935600
C	0.88200900	3.73607600	5.91550200
C	-0.19431300	4.48270800	5.49970000
S	-1.73883000	3.76462900	5.96442700
H	1.91268600	3.99691500	5.71935600
O	-3.54147800	7.02729700	1.95662100
N	-3.33617200	7.08669600	-0.31512400
O	3.49987500	6.42294700	2.56490400
N	3.68183900	6.53779600	0.31470000
C	-4.78006900	7.24094100	-0.48348800
H	-4.97509300	8.08650000	-1.14475700
H	-5.21529500	7.40421800	0.49930900
O	-3.16582600	6.97551100	-2.56551800
O	3.87360100	6.44692600	-1.95753500
C	-2.81743300	6.99621800	0.97327200
C	-1.35479600	6.86783000	1.08972300
C	-0.78841800	6.56334400	2.31633900
C	0.63047100	6.40299400	2.47440700
C	1.45981100	6.60573500	1.35552600
C	2.92470400	6.50930900	1.49109300
C	-0.53560300	6.86364300	-0.07126600
C	0.88332600	6.75018300	0.07418900
C	-2.59101600	6.95397700	-1.48873700
C	-1.12629200	6.81362400	-1.35092900
C	-0.33891800	6.49451700	-2.47020300
C	1.08282100	6.42919500	-2.31675400
C	1.69113400	6.63399700	-1.08662900
C	3.15550000	6.53019200	-0.97154800
C	5.13208000	6.46207100	0.48077200
H	5.45230200	7.24008000	1.17489000
H	5.58802000	6.59631100	-0.49707300
H	5.40812800	5.49072900	0.89822500
H	-5.19740700	6.34059700	-0.94029600
C	0.95869600	5.83687700	3.73930000
C	-0.15249000	5.50376800	4.47637100
S	-1.65056300	6.09960000	3.75581300

H	1.96660600	5.54796100	4.00174100
C	-0.75507400	5.98763000	-3.74151400
C	0.28916000	5.50324600	-4.48380200
S	1.86381700	5.85056300	-3.76422000
H	-1.79736300	5.85570000	-4.00005200
O	-3.52500800	2.28595800	-6.74559300
N	-3.53523100	0.00517900	-6.80462500
O	3.56278700	2.25161400	-6.69368500
N	3.53523100	-0.00517900	-6.80462500
C	-4.99643900	-0.03022400	-6.84255000
H	-5.31762600	-0.67137700	-7.66465300
H	-5.35189700	0.98810900	-6.97883800
O	-3.56278700	-2.25161400	-6.69368500
O	3.52500800	-2.28595800	-6.74559300
C	-2.89561900	1.23962200	-6.76422900
C	-1.42132900	1.22216600	-6.75042400
C	-0.72404800	2.39197900	-6.49764400
C	0.70977700	2.41565400	-6.44428700
C	1.41413700	1.23051000	-6.70339000
C	2.89217500	1.23293600	-6.72459400
C	-0.71421300	-0.00747000	-6.80592100
C	0.71421300	0.00747000	-6.80592100
C	-2.89217500	-1.23293600	-6.72459400
C	-1.41413700	-1.23051000	-6.70339000
C	-0.70977700	-2.41565400	-6.44428700
C	0.72404800	-2.39197900	-6.49764400
C	1.42132900	-1.22216600	-6.75042400
C	2.89561900	-1.23962200	-6.76422900
C	4.99643900	0.03022400	-6.84255000
H	5.35189700	-0.98810900	-6.97883800
H	5.38039500	0.44614800	-5.90823500
H	5.31762600	0.67137700	-7.66465300
H	-5.38039500	-0.44614800	-5.90823500
C	1.19550300	3.64873800	-5.90482000
C	0.18544800	4.48314000	-5.50598500
S	-1.41413700	3.90957900	-5.98909400
H	2.24172900	3.81437500	-5.68499400
C	-1.19550300	-3.64873800	-5.90482000
C	-0.18544800	-4.48314000	-5.50598500
S	1.41413700	-3.90957900	-5.98909400
H	-2.24172900	-3.81437500	-5.68499400
O	-3.87360100	-6.44692600	-1.95753500
N	-3.68183900	-6.53779600	0.31470000
O	3.16582600	-6.97551100	-2.56551800
N	3.33617200	-7.08669600	-0.31512400
C	-5.13208000	-6.46207100	0.48077200
H	-5.45230200	-7.24008000	1.17489000
H	-5.58802000	-6.59631100	-0.49707300
O	-3.49987500	-6.42294700	2.56490400
O	3.54147800	-7.02729700	1.95662100
C	-3.15550000	-6.53019200	-0.97154800
C	-1.69113400	-6.63399700	-1.08662900
C	-1.08282100	-6.42919500	-2.31675400
C	0.33891800	-6.49451700	-2.47020300
C	1.12629200	-6.81362400	-1.35092900
C	2.59101600	-6.95397700	-1.48873700
C	-0.88332600	-6.75018300	0.07418900

C	0.53560300	-6.86364300	-0.07126600
C	-2.92470400	-6.50930900	1.49109300
C	-1.45981100	-6.60573500	1.35552600
C	-0.63047100	-6.40299400	2.47440700
C	0.78841800	-6.56334400	2.31633900
C	1.35479600	-6.86783000	1.08972300
C	2.81743300	-6.99621800	0.97327200
C	4.78006900	-7.24094100	-0.48348800
H	5.21529500	-7.40421800	0.49930900
H	5.19740700	-6.34059700	-0.94029600
H	4.97509300	-8.08650000	-1.14475700
H	-5.40812800	-5.49072900	0.89822500
C	0.75507400	-5.98763000	-3.74151400
C	-0.28916000	-5.50324600	-4.48380200
S	-1.86381700	-5.85056300	-3.76422000
H	1.79736300	-5.85570000	-4.00005200
C	-0.95869600	-5.83687700	3.73930000
C	0.15249000	-5.50376800	4.47637100
S	1.65056300	-6.09960000	3.75581300
H	-1.96660600	-5.54796100	4.00174100

$A_3B^{2-\bullet}$ (OSS)
(U)M06-2X(D3)//6-31+G(d)
E = -7893.627987 a.u.

O	1.24929600	6.96543900	3.70348200
N	3.30677300	5.99733600	3.51844600
O	0.73722900	7.17041400	-3.36743600
N	2.79541700	6.25086300	-3.51842000
C	3.46528500	5.96260700	4.96980700
H	2.62564300	6.49492700	5.41001600
H	4.41292500	6.43179500	5.24036900
O	5.28087800	4.90878100	3.36744500
O	4.80844000	5.19336500	-3.70330200
C	2.13820600	6.53104300	2.98482600
C	2.03424500	6.55392700	1.51617800
C	0.82414600	6.86760000	0.91857400
C	0.66857100	6.88879100	-0.50922100
C	1.79858500	6.62788800	-1.30741400
C	1.70170800	6.71041700	-2.77469000
C	3.10682200	6.08294600	0.71116500
C	2.98456400	6.14366100	-0.71103000
C	4.33264000	5.40143400	2.77473300
C	4.20825900	5.42876000	1.30750400
C	5.09748300	4.68426500	0.50931700
C	4.98678600	4.79559400	-0.91851000
C	4.00696500	5.57188400	-1.51609200
C	3.92591700	5.64072000	-2.98472300
C	2.67210900	6.35621500	-4.96977900
H	1.83305800	5.74711800	-5.31394700
H	3.60262500	6.00610000	-5.40993300
H	2.47626500	7.39530000	-5.24061000
H	3.48401400	4.92604800	5.31426200
C	-0.70080300	6.97512500	-0.89167600
C	-1.55807700	6.92259300	0.18263000
S	-0.70844200	7.03171400	1.72940300
H	-1.02269500	6.91342300	-1.92161500
C	5.99169300	3.64359500	0.89184100

C	6.46647800	2.92781800	-0.18242700
S	6.04167700	3.67188900	-1.72933200
H	6.13623900	3.34948800	1.92179600
O	7.20124100	-1.38512900	-3.53775300
N	6.24001700	-3.44776000	-3.32202400
O	6.90450800	-0.58288100	3.50673500
N	6.01540900	-2.65750300	3.67185600
C	6.30814100	-3.66526600	-4.76280500
H	6.75625900	-4.64123700	-4.95777900
H	6.90502200	-2.86440200	-5.19279200
O	5.11940900	-5.40847700	-3.17495000
O	4.93240000	-4.65990700	3.87634200
C	6.73014300	-2.25053100	-2.80344900
C	6.65589200	-2.09187200	-1.35463300
C	6.92823400	-0.84860700	-0.77725300
C	6.84611100	-0.63826400	0.62705000
C	6.52246100	-1.74220100	1.46404500
C	6.50509400	-1.58386100	2.91831400
C	6.11163200	-3.12310400	-0.54257200
C	6.07339000	-2.94495700	0.88665900
C	5.58284800	-4.43799700	-2.58274200
C	5.51088000	-4.25130600	-1.13289500
C	4.71642600	-5.12088500	-0.33792100
C	4.73354100	-4.96199800	1.07454800
C	5.47126100	-3.94890100	1.69292600
C	5.43701800	-3.81067900	3.14530900
C	6.02350600	-2.47889300	5.11957400
H	7.03058500	-2.20852800	5.44169900
H	5.70177800	-3.41372700	5.57251300
H	5.34586300	-1.66793100	5.39918300
H	5.30119400	-3.66043300	-5.18809100
C	6.88451600	0.74365000	0.95808700
C	6.93671000	1.56228300	-0.14909900
S	7.14554900	0.64664000	-1.64095900
H	6.72969200	1.10032000	1.96728900
C	3.69225000	-6.01743100	-0.75584000
C	2.92659700	-6.48411800	0.28859200
S	3.56618800	-5.99257200	1.85689800
H	3.45788300	-6.17987200	-1.80016600
O	-0.98178600	-7.09946900	-3.52531800
N	-3.04028100	-6.11583000	-3.52905800
O	-0.98906800	-7.03329400	3.56527700
N	-3.04887800	-6.10559500	3.53131700
C	-3.09166700	-6.13486800	-4.98852600
H	-4.01054300	-6.62789300	-5.31135100
H	-2.21525900	-6.66977700	-5.34616900
O	-5.02137300	-5.03019600	-3.56328700
O	-5.07420900	-5.05549000	3.52773300
C	-1.91782700	-6.63456200	-2.89097200
C	-1.92148400	-6.60226600	-1.41862000
C	-0.75995400	-6.89523700	-0.72284300
C	-0.71187300	-6.85889200	0.71197100
C	-1.89393500	-6.56501300	1.41440900
C	-1.90549800	-6.59357100	2.88775400
C	-3.04847300	-6.09982100	-0.71258300
C	-3.03315400	-6.10625000	0.71482800
C	-4.11815400	-5.49629000	-2.88565600

C	-4.10146800	-5.46796300	-1.41236100
C	-5.04802500	-4.70100200	-0.71034800
C	-5.04732100	-4.75968400	0.72456100
C	-4.11285600	-5.50877200	1.42078400
C	-4.13969100	-5.52322800	2.89323200
C	-3.03186200	-6.15589200	4.99084700
H	-3.98610700	-5.77723000	5.34882400
H	-2.21004400	-5.54460500	5.37052400
H	-2.87243600	-7.18595200	5.31516800
H	-3.09817700	-5.11131400	-5.36995700
C	0.62806600	-6.92855600	1.19706000
C	1.56004600	-6.93185900	0.18741100
S	0.82665800	-7.11290200	-1.41209300
H	0.87505400	-6.80558900	2.24293900
C	-5.91131100	-3.67429400	-1.19621900
C	-6.47539600	-2.93160400	-0.18713000
S	-6.17775800	-3.62468200	1.41290000
H	-5.96222000	-3.40397500	-2.24232500
O	-6.69072900	1.12842100	-3.87777200
N	-5.74349100	3.19840600	-3.67279300
O	-7.40443900	0.82774000	3.17349000
N	-6.51279700	2.90259200	3.32081600
C	-5.60497200	3.31238800	-5.12044200
H	-5.99518400	4.27927700	-5.44281400
H	-6.15739200	2.49273400	-5.57380500
O	-4.62251100	5.15749900	-3.50713200
O	-5.44457000	4.91184200	3.53668200
C	-6.31637200	2.04234300	-3.14646700
C	-6.44829100	1.98660200	-1.69411300
C	-6.81347300	0.78774900	-1.07570400
C	-6.93088100	0.67875800	0.33674900
C	-6.71523000	1.83675400	1.13163600
C	-6.90789900	1.78181400	2.58143600
C	-6.00964600	3.07196600	-0.88778100
C	-6.17567400	2.99551900	0.54138600
C	-5.18134800	4.23577600	-2.91903100
C	-5.31930600	4.15454400	-1.46486300
C	-4.63323800	5.07772600	-0.62762400
C	-4.85128700	5.01698200	0.77652500
C	-5.68028800	4.05099500	1.35356400
C	-5.85188900	4.01471000	2.80233600
C	-6.72700500	2.82578800	4.76165300
H	-6.44663000	3.78441400	5.19171900
H	-6.11673700	2.02459400	5.18650800
H	-7.77598000	2.59603900	4.95703500
H	-4.54931100	3.25958500	-5.39948700
C	-7.03023000	-0.67864700	0.75495500
C	-6.94129400	-1.57127400	-0.28916800
S	-6.93328800	-0.76499400	-1.85770700
H	-7.01912000	-0.96328100	1.79935800
C	-3.55276800	5.94030200	-0.95830600
C	-2.93131100	6.47522800	0.14907300
S	-3.78864200	6.09094400	1.64052600
H	-3.17423200	6.03112800	-1.96732000

$A_3B^{2-\bullet\bullet}$ (triplet)
(U)M06-2X(D3)//6-31+G(d)

E = -7893.625006 a.u.

O	-3.70224100	1.99976400	6.78243300
N	-3.50868000	-0.27720700	6.86359800
O	3.37316000	2.53351900	6.73725600
N	3.50868000	0.27720700	6.86359800
C	-4.95856200	-0.42843400	6.90809400
H	-5.39171600	0.55689800	7.06342700
H	-5.22322500	-1.11010300	7.71833100
O	-3.37316000	-2.53351900	6.73725600
O	3.70224100	-1.99976400	6.78243300
C	-2.97375900	1.00901200	6.80760900
C	-1.52008400	1.10556700	6.78685000
C	-0.90734100	2.33822400	6.52491400
C	0.50314900	2.47805700	6.47245600
C	1.31461900	1.33934600	6.73901300
C	2.77065300	1.46276400	6.77112100
C	-0.71836600	-0.06549500	6.81965800
C	0.71836600	0.06549500	6.81965800
C	-2.77065300	-1.46276400	6.77112100
C	-1.31461900	-1.33934600	6.73901300
C	-0.50314900	-2.47805700	6.47245600
C	0.90734100	-2.33822400	6.52491400
C	1.52008400	-1.10556700	6.78685000
C	2.97375900	-1.00901200	6.80760900
C	4.95856200	0.42843400	6.90809400
H	5.31805500	0.85806000	5.96928100
H	5.39171600	-0.55689800	7.06342700
H	5.22322500	1.11010300	7.71833100
H	-5.31805500	-0.85806000	5.96928100
C	0.89172600	3.73133000	5.91708800
C	-0.17859000	4.49319900	5.51071900
S	-1.71952000	3.77536900	5.97516800
H	1.92530300	3.97682300	5.71270600
C	-0.89172600	-3.73133000	5.91708800
C	0.17859000	-4.49319900	5.51071900
S	1.71952000	-3.77536900	5.97516800
H	-1.92530300	-3.97682300	5.71270600
O	3.51928500	-7.09299200	1.96942400
N	3.32340400	-7.12938400	-0.30263500
O	-3.51981800	-6.45804700	2.55165300
N	-3.68894000	-6.53107400	0.30028600
C	4.76584500	-7.29164100	-0.46641500
H	4.96317100	-8.17069900	-1.08278100
H	5.20367000	-7.40244500	0.52256300
O	3.16506700	-7.05653000	-2.55466900
O	-3.87726600	-6.47812200	-1.97226000
C	2.80012700	-7.04079400	0.98325200
C	1.33684400	-6.89229200	1.09411400
C	0.76976000	-6.58189600	2.31846400
C	-0.64925500	-6.41374800	2.47227700
C	-1.47044800	-6.61664900	1.35158700
C	-2.93688800	-6.52290000	1.48093000
C	0.52349800	-6.89023200	-0.07008600
C	-0.88963200	-6.76806300	0.07008900
C	2.58196500	-7.00704900	-1.48345700
C	1.12090700	-6.84732100	-1.35307600
C	0.34609400	-6.51590600	-2.47337200

C	-1.08132400	-6.44107600	-2.32236500
C	-1.69216200	-6.64278100	-1.09648200
C	-3.15902200	-6.54139700	-0.98592300
C	-5.13804500	-6.44549400	0.46374200
H	-5.47888100	-7.26621500	1.09751500
H	-5.58913100	-6.50138600	-0.52392000
H	-5.39870900	-5.50315100	0.95082000
H	5.17889800	-6.41633100	-0.97266200
C	-0.98092600	-5.84454200	3.73950700
C	0.12708400	-5.50580200	4.47504800
S	1.62618500	-6.11418900	3.76198500
H	-1.98752200	-5.54772300	3.99739500
C	0.76963400	-6.01628500	-3.74475800
C	-0.26571400	-5.50727300	-4.48535900
S	-1.84865500	-5.85726400	-3.77560100
H	1.81251200	-5.88743500	-4.00072100
O	3.53823900	-2.27905800	-6.78447000
N	3.51928500	0.00643300	-6.84981100
O	-3.55654000	-2.26646800	-6.72119300
N	-3.51928500	-0.00643300	-6.84981100
C	4.97646700	0.04692100	-6.89462300
H	5.29272600	0.70039700	-7.70974400
H	5.33329600	-0.96960600	-7.04276400
O	3.55654000	2.26646800	-6.72119300
O	-3.53823900	2.27905800	-6.78447000
C	2.88722600	-1.23563000	-6.80240300
C	1.43036000	-1.22088900	-6.77996800
C	0.72505800	-2.40332100	-6.52161500
C	-0.69101600	-2.43075800	-6.46148800
C	-1.41306000	-1.23314700	-6.72237900
C	-2.87442900	-1.24477900	-6.75501000
C	0.72079200	0.00923900	-6.80685500
C	-0.72079200	-0.00923900	-6.80685500
C	2.87442900	1.24477900	-6.75501000
C	1.41306000	1.23314700	-6.72237900
C	0.69101600	2.43075800	-6.46148800
C	-0.72505800	2.40332100	-6.52161500
C	-1.43036000	1.22088900	-6.77996800
C	-2.88722600	1.23563000	-6.80240300
C	-4.97646700	-0.04692100	-6.89462300
H	-5.33329600	0.96960600	-7.04276400
H	-5.36684800	-0.45533000	-5.95877900
H	-5.29272600	-0.70039700	-7.70974400
H	5.36684800	0.45533000	-5.95877900
C	-1.17282400	-3.65266700	-5.90509800
C	-0.16308400	-4.50167900	-5.51850400
S	1.42667900	-3.90961700	-5.99581400
H	-2.22039000	-3.81076500	-5.68171800
C	1.17282400	3.65266700	-5.90509800
C	0.16308400	4.50167900	-5.51850400
S	-1.42667900	3.90961700	-5.99581400
H	2.22039000	3.81076500	-5.68171800
O	3.87726600	6.47812200	-1.97226000
N	3.68894000	6.53107400	0.30028600
O	-3.16506700	7.05653000	-2.55466900
N	-3.32340400	7.12938400	-0.30263500
C	5.13804500	6.44549400	0.46374200

H	5.47888100	7.26621500	1.09751500
H	5.58913100	6.50138600	-0.52392000
O	3.51981800	6.45804700	2.55165300
O	-3.51928500	7.09299200	1.96942400
C	3.15902200	6.54139700	-0.98592300
C	1.69216200	6.64278100	-1.09648200
C	1.08132400	6.44107600	-2.32236500
C	-0.34609400	6.51590600	-2.47337200
C	-1.12090700	6.84732100	-1.35307600
C	-2.58196500	7.00704900	-1.48345700
C	0.88963200	6.76806300	0.07008900
C	-0.52349800	6.89023200	-0.07008600
C	2.93688800	6.52290000	1.48093000
C	1.47044800	6.61664900	1.35158700
C	0.64925500	6.41374800	2.47227700
C	-0.76976000	6.58189600	2.31846400
C	-1.33684400	6.89229200	1.09411400
C	-2.80012700	7.04079400	0.98325200
C	-4.76584500	7.29164100	-0.46641500
H	-5.20367000	7.40244500	0.52256300
H	-5.17889800	6.41633100	-0.97266200
H	-4.96317100	8.17069900	-1.08278100
H	5.39870900	5.50315100	0.95082000
C	-0.76963400	6.01628500	-3.74475800
C	0.26571400	5.50727300	-4.48535900
S	1.84865500	5.85726400	-3.77560100
H	-1.81251200	5.88743500	-4.00072100
C	0.98092600	5.84454200	3.73950700
C	-0.12708400	5.50580200	4.47504800
S	-1.62618500	6.11418900	3.76198500
H	1.98752200	5.54772300	3.99739500

AB$_3$ (singlet)
M06-2X(D3)//6-31+G(d)
E = -7893.429694 a.u.

O	3.76075600	1.86283900	6.69455000
N	3.51895200	-0.40171500	6.81797900
O	-3.28204600	2.64680500	6.72556600
N	-3.51895200	0.40171500	6.81797900
C	4.96880300	-0.60019700	6.86237800
H	5.43464100	0.36888100	7.02264600
H	5.20784900	-1.28924400	7.67290100
O	3.28204600	-2.64680500	6.72556600
O	-3.76075600	-1.86283900	6.69455000
C	3.02256800	0.89455000	6.74849400
C	1.55137900	1.04691500	6.75008900
C	0.99119400	2.28499000	6.49140600
C	-0.43265300	2.47632700	6.46197700
C	-1.26520900	1.38564500	6.72761300
C	-2.73922900	1.55694000	6.74997900
C	0.70782600	-0.08902500	6.82830500
C	-0.70782600	0.08902500	6.82830500
C	2.73922900	-1.55694000	6.74997900
C	1.26520900	-1.38564500	6.72761300
C	0.43265300	-2.47632700	6.46197700
C	-0.99119400	-2.28499000	6.49140600
C	-1.55137900	-1.04691500	6.75008900

C	-3.02256800	-0.89455000	6.74849400
C	-4.96880300	0.60019700	6.86237800
H	-5.31247900	1.03503000	5.92104000
H	-5.43464100	-0.36888100	7.02264600
H	-5.20784900	1.28924400	7.67290100
H	5.31247900	-1.03503000	5.92104000
C	-0.78080300	3.76149300	5.92573700
C	0.31145200	4.45658700	5.49404700
S	1.84313300	3.69862500	5.93668600
H	-1.80444400	4.05771300	5.74459100
C	0.78080300	-3.76149300	5.92573700
C	-0.31145200	-4.45658700	5.49404700
S	-1.84313300	-3.69862500	5.93668600
H	1.80444400	-4.05771300	5.74459100
O	-3.71748800	-6.92030100	1.95062400
N	-3.52397400	-7.00671500	-0.32059900
O	3.32841500	-6.47921100	2.56481100
N	3.51895200	-6.60297200	0.31675000
C	-4.97308900	-7.13439800	-0.48681700
H	-5.18167100	-7.96484600	-1.16208900
H	-5.40826400	-7.30963700	0.49382500
O	-3.34362700	-6.89920300	-2.57035100
O	3.70333800	-6.50991700	-1.95490300
C	-3.00166800	-6.91909600	0.96446400
C	-1.52968000	-6.82906100	1.08272000
C	-0.95791000	-6.53554100	2.30776000
C	0.46451800	-6.40824900	2.46186200
C	1.28700000	-6.62706300	1.35267100
C	2.76307900	-6.55727700	1.48894100
C	-0.70937700	-6.85661700	-0.07184700
C	0.70715800	-6.77346100	0.07109000
C	-2.77644800	-6.89267500	-1.49311800
C	-1.30184000	-6.78761200	-1.35553600
C	-0.51062800	-6.48448500	-2.46442300
C	0.91764900	-6.44746800	-2.31290700
C	1.51905400	-6.66436900	-1.08658700
C	2.99064400	-6.58456100	-0.96890400
C	4.97308900	-6.56359300	0.48183900
H	5.27163200	-7.34341300	1.18297100
H	5.42522000	-6.72109800	-0.49417200
H	5.27439600	-5.59498500	0.88731100
H	-5.37718300	-6.21858900	-0.92389300
C	0.80692200	-5.83819000	3.73340200
C	-0.29417600	-5.48967700	4.46121100
S	-1.80825600	-6.04840100	3.74813400
H	1.82046100	-5.56689600	3.99274700
C	-0.91630000	-5.96621900	-3.74241400
C	0.13814600	-5.50601800	-4.47491700
S	1.70874400	-5.88873400	-3.76282800
H	-1.95512300	-5.81257800	-4.00248000
O	-3.58899600	-2.17773300	-6.71394100
N	-3.53992200	0.10123000	-6.79785000
O	3.49364000	-2.35739900	-6.68237100
N	3.53992200	-0.10123000	-6.79785000
C	-5.00135500	0.17776800	-6.84229400
H	-5.29766100	0.84602300	-7.65143300
H	-5.38438600	-0.82650800	-7.00496600

O	-3.49364000	2.35739900	-6.68237100
O	3.58899600	2.17773300	-6.71394100
C	-2.93535400	-1.14963700	-6.74810500
C	-1.45674800	-1.17707800	-6.74241400
C	-0.79490400	-2.36511600	-6.48964600
C	0.63926800	-2.42819800	-6.44105700
C	1.37744800	-1.27172600	-6.69936400
C	2.86117000	-1.31763100	-6.71745200
C	-0.71225700	0.02735300	-6.80799400
C	0.71225700	-0.02735300	-6.80799400
C	-2.86117000	1.31763100	-6.71745200
C	-1.37744800	1.27172600	-6.69936400
C	-0.63926800	2.42819800	-6.44105700
C	0.79490400	2.36511600	-6.48964600
C	1.45674800	1.17707800	-6.74241400
C	2.93535400	1.14963700	-6.74810500
C	5.00135500	-0.17776800	-6.84229400
H	5.38438600	0.82650800	-7.00496600
H	5.37963900	-0.58077200	-5.90014700
H	5.29766100	-0.84602300	-7.65143300
H	-5.37963900	0.58077200	-5.90014700
C	1.09121900	-3.68061900	-5.89905200
C	0.05808600	-4.47867800	-5.50362200
S	-1.52739100	-3.86242600	-5.97862100
H	2.13300700	-3.87725900	-5.68368100
C	-1.09121900	3.68061900	-5.89905200
C	-0.05808600	4.47867800	-5.50362200
S	1.52739100	3.86242600	-5.97862100
H	-2.13300700	3.87725900	-5.68368100
O	-3.70333800	6.50991700	-1.95490300
N	-3.51895200	6.60297200	0.31675000
O	3.34362700	6.89920300	-2.57035100
N	3.52397400	7.00671500	-0.32059900
C	-4.97308900	6.56359300	0.48183900
H	-5.27163200	7.34341300	1.18297100
H	-5.42522000	6.72109800	-0.49417200
O	-3.32841500	6.47921100	2.56481100
O	3.71748800	6.92030100	1.95062400
C	-2.99064400	6.58456100	-0.96890400
C	-1.51905400	6.66436900	-1.08658700
C	-0.91764900	6.44746800	-2.31290700
C	0.51062800	6.48448500	-2.46442300
C	1.30184000	6.78761200	-1.35553600
C	2.77644800	6.89267500	-1.49311800
C	-0.70715800	6.77346100	0.07109000
C	0.70937700	6.85661700	-0.07184700
C	-2.76307900	6.55727700	1.48894100
C	-1.28700000	6.62706300	1.35267100
C	-0.46451800	6.40824900	2.46186200
C	0.95791000	6.53554100	2.30776000
C	1.52968000	6.82906100	1.08272000
C	3.00166800	6.91909600	0.96446400
C	4.97308900	7.13439800	-0.48681700
H	5.40826400	7.30963700	0.49382500
H	5.37718300	6.21858900	-0.92389300
H	5.18167100	7.96484600	-1.16208900
H	-5.27439600	5.59498500	0.88731100

C	0.91630000	5.96621900	-3.74241400
C	-0.13814600	5.50601800	-4.47491700
S	-1.70874400	5.88873400	-3.76282800
H	1.95512300	5.81257800	-4.00248000
C	-0.80692200	5.83819000	3.73340200
C	0.29417600	5.48967700	4.46121100
S	1.80825600	6.04840100	3.74813400
H	-1.82046100	5.56689600	3.99274700

$AB_3^{-\bullet}$
(U)M06-2X(D3)//6-31+G(d)
E = -7893.559483 a.u.

O	3.70152400	1.99095100	6.74047600
N	3.52062700	-0.28276400	6.82212500
O	-3.36816600	2.53464200	6.69414200
N	-3.52062700	0.28276400	6.82212500
C	4.97336900	-0.43607200	6.86160800
H	5.40872400	0.54618900	7.02819300
H	5.23741200	-1.12558500	7.66450700
O	3.36816600	-2.53464200	6.69414200
O	-3.70152400	-1.99095100	6.74047600
C	2.98515100	0.99993900	6.76956100
C	1.51829600	1.10139200	6.75693900
C	0.91648000	2.32556200	6.50059600
C	-0.50812700	2.47060400	6.45123900
C	-1.31034500	1.34149100	6.71474000
C	-2.77875200	1.46471700	6.73462200
C	0.71361400	-0.06725500	6.80977500
C	-0.71361400	0.06725500	6.80977500
C	2.77875200	-1.46471700	6.73462200
C	1.31034500	-1.34149100	6.71474000
C	0.50812700	-2.47060400	6.45123900
C	-0.91648000	-2.32556200	6.50059600
C	-1.51829600	-1.10139200	6.75693900
C	-2.98515100	-0.99993900	6.76956100
C	-4.97336900	0.43607200	6.86160800
H	-5.32901000	0.85347300	5.91639700
H	-5.40872400	-0.54618900	7.02819300
H	-5.23741200	1.12558500	7.66450700
H	5.32901000	-0.85347300	5.91639700
C	-0.89163900	3.73318300	5.91413800
C	0.18331800	4.48263500	5.50059900
S	1.72897000	3.76968500	5.96936600
H	-1.92268000	3.99062300	5.71530300
C	0.89163900	-3.73318300	5.91413800
C	-0.18331800	-4.48263500	5.50059900
S	-1.72897000	-3.76968500	5.96936600
H	1.92268000	-3.99062300	5.71530300
O	-3.53495300	-7.02861600	1.96423700
N	-3.33427900	-7.08882300	-0.30787200
O	3.50709400	-6.42055800	2.55783800
N	3.68460900	-6.53811000	0.30738500
C	-4.77847100	-7.24403800	-0.47267100
H	-4.97373400	-8.08229600	-1.14293900
H	-5.20977100	-7.41927700	0.50980900
O	-3.16945200	-6.97276700	-2.55861600
O	3.87187500	-6.44820200	-1.96523900

C	-2.81292700	-6.99737400	0.97933700
C	-1.35020100	-6.86815700	1.09261800
C	-0.78129100	-6.56326200	2.31812400
C	0.63756600	-6.40224000	2.47327700
C	1.46464300	-6.60475600	1.35267700
C	2.92992900	-6.50840200	1.48518200
C	-0.53364000	-6.86341300	-0.06995700
C	0.88552900	-6.74975100	0.07265600
C	-2.59199400	-6.95341500	-1.48314900
C	-1.12713100	-6.81328600	-1.34852300
C	-0.34209300	-6.49359900	-2.46929600
C	1.08016900	-6.42899900	-2.31865200
C	1.69098900	-6.63395400	-1.08992100
C	3.15558100	-6.53087000	-0.97791900
C	5.13524200	-6.46259800	0.47030600
H	5.45630200	-7.23848300	1.16636300
H	5.58921700	-6.60026000	-0.50797300
H	5.41272800	-5.48999500	0.88392800
H	-5.19976300	-6.33938400	-0.91739000
C	0.96806800	-5.83564600	3.73773900
C	-0.14181500	-5.50384600	4.47701800
S	-1.64105300	-6.09992500	3.75901400
H	1.97642500	-5.54622600	3.99801400
C	-0.76045500	-5.98669100	-3.73973100
C	0.28271500	-5.50278200	-4.48406000
S	1.85862300	-5.85129400	-3.76759100
H	-1.80328100	-5.85429300	-3.99603500
O	-3.52951200	-2.27934100	-6.74408000
N	-3.53495300	0.00128200	-6.80888000
O	3.55876500	-2.25804300	-6.69626700
N	3.53495300	-0.00128200	-6.80888000
C	-4.99607100	0.03865600	-6.84827100
H	-5.31483400	0.69079500	-7.66249400
H	-5.35242200	-0.97749400	-6.99787400
O	-3.55876500	2.25804300	-6.69626700
O	3.52951200	2.27934100	-6.74408000
C	-2.89782600	-1.23433000	-6.76491200
C	-1.42371900	-1.21966600	-6.75126300
C	-0.72837500	-2.39069700	-6.49793400
C	0.70531000	-2.41698800	-6.44611900
C	1.41186300	-1.23296800	-6.70575200
C	2.88974800	-1.23820100	-6.72738600
C	-0.71427000	0.00867900	-6.80727700
C	0.71427000	-0.00867900	-6.80727700
C	-2.88974800	1.23820100	-6.72738600
C	-1.41186300	1.23296800	-6.70575200
C	-0.70531000	2.41698800	-6.44611900
C	0.72837500	2.39069700	-6.49793400
C	1.42371900	1.21966600	-6.75126300
C	2.89782600	1.23433000	-6.76491200
C	4.99607100	-0.03865600	-6.84827100
H	5.35242200	0.97749400	-6.99787400
H	5.38154400	-0.44260500	-5.90921600
H	5.31483400	-0.69079500	-7.66249400
H	-5.38154400	0.44260500	-5.90921600
C	1.18955200	-3.65058500	-5.90707700
C	0.17853500	-4.48314200	-5.50634700

S	-1.42070000	-3.90661800	-5.98755200
H	2.23587900	-3.81767100	-5.68884000
C	-1.18955200	3.65058500	-5.90707700
C	-0.17853500	4.48314200	-5.50634700
S	1.42070000	3.90661800	-5.98755200
H	-2.23587900	3.81767100	-5.68884000
O	-3.87187500	6.44820200	-1.96523900
N	-3.68460900	6.53811000	0.30738500
O	3.16945200	6.97276700	-2.55861600
N	3.33427900	7.08882300	-0.30787200
C	-5.13524200	6.46259800	0.47030600
H	-5.45630200	7.23848300	1.16636300
H	-5.58921700	6.60026000	-0.50797300
O	-3.50709400	6.42055800	2.55783800
O	3.53495300	7.02861600	1.96423700
C	-3.15558100	6.53087000	-0.97791900
C	-1.69098900	6.63395400	-1.08992100
C	-1.08016900	6.42899900	-2.31865200
C	0.34209300	6.49359900	-2.46929600
C	1.12713100	6.81328600	-1.34852300
C	2.59199400	6.95341500	-1.48314900
C	-0.88552900	6.74975100	0.07265600
C	0.53364000	6.86341300	-0.06995700
C	-2.92992900	6.50840200	1.48518200
C	-1.46464300	6.60475600	1.35267700
C	-0.63756600	6.40224000	2.47327700
C	0.78129100	6.56326200	2.31812400
C	1.35020100	6.86815700	1.09261800
C	2.81292700	6.99737400	0.97933700
C	4.77847100	7.24403800	-0.47267100
H	5.20977100	7.41927700	0.50980900
H	5.19976300	6.33938400	-0.91739000
H	4.97373400	8.08229600	-1.14293900
H	-5.41272800	5.48999500	0.88392800
C	0.76045500	5.98669100	-3.73973100
C	-0.28271500	5.50278200	-4.48406000
S	-1.85862300	5.85129400	-3.76759100
H	1.80328100	5.85429300	-3.99603500
C	-0.96806800	5.83564600	3.73773900
C	0.14181500	5.50384600	4.47701800
S	1.64105300	6.09992500	3.75901400
H	-1.97642500	5.54622600	3.99801400

$AB_3^{2-\bullet\bullet}$ (OSS)
(U)M06-2X(D3)//6-31+G(d)
E = -7893.627977 a.u.

O	6.74081700	2.15552500	3.70316800
N	5.50947300	4.06726900	3.51884800
O	7.01197200	1.67735200	-3.36772700
N	5.82938300	3.59661400	-3.51818500
C	5.45370800	4.21918000	4.97024700
H	6.09244500	3.45723800	5.41029400
H	5.79310000	5.22053800	5.24128700
O	4.16979800	5.88035000	3.36835100
O	4.51500500	5.45220600	-3.70261500
C	6.19319100	2.97981500	2.98486300
C	6.22981900	2.88037800	1.51618800

C	6.70079000	1.72253300	0.91832200
C	6.74230800	1.57156600	-0.50954500
C	6.33430700	2.65736600	-1.30747500
C	6.42899600	2.57274500	-2.77477200
C	5.62107300	3.88142800	0.71139000
C	5.69748100	3.76875600	-0.71082400
C	4.78331000	5.00561200	2.77538100
C	4.82684300	4.88641700	1.30804900
C	3.97144400	5.66974900	0.51009700
C	4.09637700	5.57509000	-0.91774800
C	4.99551300	4.70678400	-1.51558100
C	5.07485300	4.63622500	-2.98424600
C	5.95039700	3.48892400	-4.96956100
H	5.45578000	2.57796000	-5.31450400
H	5.48223200	4.36615300	-5.40938900
H	7.00623900	3.43016700	-5.23996000
H	4.42369100	4.10031300	5.31435700
C	7.00871300	0.22567000	-0.89241500
C	7.07001700	-0.63131000	0.18161100
S	7.06614400	0.22497700	1.72874500
H	6.98982400	-0.10122700	-1.92244500
C	2.82167400	6.41847000	0.89279100
C	2.04952000	6.79481500	-0.18140900
S	2.84300300	6.47241900	-1.72829600
H	2.51107800	6.52275600	1.92277000
O	-2.32260300	6.95434200	-3.53622400
N	-4.24138700	5.73099800	-3.32073100
O	-1.48683900	6.76211400	3.50784500
N	-3.42653600	5.60798200	3.67290900
C	-4.46575700	5.77032400	-4.76153300
H	-5.49254300	6.08492000	-4.95652500
H	-3.75112200	6.46838300	-5.19111000
O	-6.03819100	4.36287200	-3.17403000
O	-5.26946100	4.27161200	3.87735100
C	-3.11867800	6.37359700	-2.80202300
C	-2.95151500	6.32031500	-1.35326300
C	-1.75476700	6.75370300	-0.77596400
C	-1.53523800	6.69915800	0.62824700
C	-2.58657400	6.23220900	1.46521200
C	-2.42674400	6.23479500	2.91941900
C	-3.90206000	5.64480800	-0.54137100
C	-3.72003700	5.62936800	0.88782000
C	-5.13667300	4.94919200	-2.58168800
C	-4.94183500	4.90158600	-1.13192800
C	-5.69947100	3.99946200	-0.33726200
C	-5.54384500	4.03647300	1.07523100
C	-4.63607100	4.90026800	1.69392600
C	-4.49401000	4.88353600	3.14628100
C	-3.25007000	5.63880700	5.12060400
H	-3.11440300	6.67254700	5.44317900
H	-4.13428100	5.19675200	5.57370900
H	-2.35692200	5.07361700	5.39970800
H	-4.32708700	4.77316200	-5.18733200
C	-0.17047500	6.91958200	0.95925700
C	0.63375600	7.08040100	-0.14798200
S	-0.30149000	7.16721900	-1.63961300
H	0.20367700	6.81245400	1.96831900

C	-6.45401900	2.86677200	-0.75560500
C	-6.81584400	2.04588500	0.28845500
S	-6.41228600	2.74372600	1.85709000
H	-6.58440400	2.61352000	-1.80002400
O	-6.90579300	-1.90714000	-3.52734800
N	-5.65956200	-3.81808200	-3.53067000
O	-6.84642100	-1.90938100	3.56345300
N	-5.65546100	-3.82904800	3.52966600
C	-5.67021800	-3.87075600	-4.99016400
H	-6.03589300	-4.84703900	-5.31380700
H	-6.31691900	-3.07332400	-5.34793600
O	-4.32231600	-5.63884800	-3.56450400
O	-4.34687300	-5.69786700	3.52637900
C	-6.32239800	-2.77411700	-2.89274200
C	-6.29142800	-2.77425100	-1.42032400
C	-6.73598800	-1.66202100	-0.72439100
C	-6.70764000	-1.61026700	0.71046400
C	-6.26093200	-2.74353900	1.41273000
C	-6.28927000	-2.75963400	2.88604200
C	-5.64519800	-3.82540700	-0.71422100
C	-5.65505500	-3.81187300	0.71319600
C	-4.90369000	-4.80500200	-2.88704400
C	-4.87918200	-4.78542900	-1.41368000
C	-3.99441300	-5.62256300	-0.71111400
C	-4.05383300	-5.63018600	0.72370900
C	-4.92085300	-4.80345900	1.41941300
C	-4.93323400	-4.83287400	2.89179800
C	-5.70941800	-3.81990500	4.98912300
H	-5.20990800	-4.71686700	5.34699900
H	-5.21105100	-2.92569500	5.37022700
H	-6.75191000	-3.79644700	5.31205200
H	-4.65459100	-3.74039900	-5.37060200
C	-6.95394100	-0.29150700	1.19596500
C	-7.07959000	0.63233300	0.18662300
S	-7.16144500	-0.11785100	-1.41325000
H	-6.86537000	-0.03083000	2.24200300
C	-2.86196800	-6.34212200	-1.19619600
C	-2.05194000	-6.80314300	-0.18658000
S	-2.77928500	-6.59996400	1.41296800
H	-2.58649500	-6.35661300	-2.24208000
O	1.99962400	-6.48105700	-3.87700400
N	3.92725400	-5.26999700	-3.67286400
O	1.79780200	-7.22814800	3.17414000
N	3.73788700	-6.07203900	3.32080700
C	4.02136000	-5.11759500	-5.12056600
H	5.03072100	-5.37821000	-5.44358900
H	3.28055400	-5.77229100	-5.57350700
O	5.72170000	-3.90083700	-3.50793100
O	5.58933400	-4.74893200	3.53612700
C	2.85689900	-5.99005900	-3.14611000
C	2.81950600	-6.12802000	-1.69377900
C	1.67935800	-6.64788100	-1.07500400
C	1.58704700	-6.77838600	0.33748400
C	2.70681300	-6.41220300	1.13206000
C	2.67827000	-6.61064900	2.58181400
C	3.83787800	-5.55035000	-0.88774800
C	3.78434300	-5.72498800	0.54143900

C	4.88203200	-4.57638200	-2.91945600
C	4.82009900	-4.72365100	-1.46525100
C	5.64532300	-3.92216900	-0.62829900
C	5.61413000	-4.14632200	0.77590800
C	4.76581500	-5.09517000	1.35329100
C	4.75320500	-5.27047300	2.80203400
C	3.69085900	-6.29531800	4.76155600
H	4.60511600	-5.89270300	5.19117300
H	2.81733900	-5.79499300	5.18757400
H	3.59996800	-7.36541800	4.95623900
H	3.83099900	-4.07784300	-5.39934000
C	0.25464400	-7.05574100	0.75594900
C	-0.64192200	-7.08530900	-0.28813600
S	0.15599100	-6.97149600	-1.85682700
H	-0.02882400	-7.08223200	1.80042000
C	6.35857900	-2.73789700	-0.95924200
C	6.80756800	-2.05150000	0.14797500
S	6.53939500	-2.95183400	1.63967400
H	6.39858900	-2.35071100	-1.96828000

$AB_3^{2-\bullet\bullet}$ (triplet)
(U)M06-2X(D3)//6-31+G(d)
E = -7893.625001 a.u.

O	3.93354800	1.49447500	6.78411600
N	3.44158600	-0.73694300	6.86558100
O	-3.01023000	2.95568200	6.73772300
N	-3.44158600	0.73694300	6.86558100
C	4.85897300	-1.07758500	6.90986100
H	5.41769400	-0.15844000	7.07002600
H	5.03079300	-1.79194400	7.71688700
O	3.01023000	-2.95568200	6.73772300
O	-3.93354800	-1.49447500	6.78411600
C	3.08070100	0.60846500	6.80966000
C	1.65242000	0.89573400	6.78893500
C	1.20738100	2.19826000	6.52671400
C	-0.17244400	2.52261000	6.47365500
C	-1.12683500	1.50086100	6.74065400
C	-2.55390500	1.81498800	6.77248100
C	0.70345600	-0.15954800	6.82174500
C	-0.70345600	0.15954800	6.82174500
C	2.55390500	-1.81498800	6.77248100
C	1.12683500	-1.50086100	6.74065400
C	0.17244400	-2.52261000	6.47365500
C	-1.20738100	-2.19826000	6.52671400
C	-1.65242000	-0.89573400	6.78893500
C	-3.08070100	-0.60846500	6.80966000
C	-4.85897300	1.07758500	6.90986100
H	-5.16005100	1.54613700	5.96908400
H	-5.41769400	0.15844000	7.07002600
H	-5.03079300	1.79194400	7.71688700
H	5.16005100	-1.54613700	5.96908400
C	-0.39239800	3.81580100	5.91749600
C	0.76905400	4.42995300	5.51122700
S	2.20179300	3.51557700	5.97626500
H	-1.38454100	4.19500700	5.71230900
C	0.39239800	-3.81580100	5.91749600
C	-0.76905400	-4.42995300	5.51122700

S	-2.20179300	-3.51557700	5.97626500
H	1.38454100	-4.19500700	5.71230900
O	-4.42373100	-6.56347900	1.96950300
N	-4.23463400	-6.62443600	-0.30268100
O	2.63760200	-6.86469700	2.55087700
N	2.79517700	-6.96135900	0.29960900
C	-5.68582100	-6.59335900	-0.46654200
H	-5.99941900	-7.44245900	-1.07680500
H	-6.13432000	-6.63779300	0.52286400
O	-4.06821700	-6.57308500	-2.55473800
O	2.98828300	-6.93659100	-1.97313000
C	-3.70419200	-6.60679300	0.98320000
C	-2.23411500	-6.65324300	1.09387400
C	-1.63084200	-6.42088700	2.31819800
C	-0.20199900	-6.44158300	2.47184500
C	0.58505600	-6.75133600	1.35111300
C	2.05100000	-6.85249900	1.48026600
C	-1.42772500	-6.75870400	-0.07040900
C	-0.01090200	-6.82500200	0.06971800
C	-3.48357700	-6.60167200	-1.48361600
C	-2.01424100	-6.63719300	-1.35338600
C	-1.20233800	-6.41198700	-2.47384900
C	0.22236000	-6.52741400	-2.32284200
C	0.80102600	-6.80802500	-1.09695700
C	2.26835700	-6.90273700	-0.98661500
C	4.24288900	-7.06776300	0.46316900
H	4.47298600	-7.92919100	1.09284500
H	4.68309200	-7.17783100	-0.52484000
H	4.62459100	-6.17015200	0.95480500
H	-5.97776700	-5.67426500	-0.97933400
C	0.20199900	-5.92132900	3.73906500
C	-0.85155600	-5.43994200	4.47508900
S	-2.41785000	-5.84500000	3.76213700
H	1.23898400	-5.75993900	3.99680500
C	-1.55582800	-5.86106200	-3.74541900
C	-0.46209300	-5.49437000	-4.48629500
S	1.06040500	-6.05115900	-3.77637900
H	-2.57234300	-5.59479000	-4.00136100
O	-3.80668300	-1.79475400	-6.78679100
N	-3.48793900	0.46856000	-6.85042000
O	3.22815200	-2.71378300	-6.71875600
N	3.48793900	-0.46856000	-6.85042000
C	-4.92721900	0.69999300	-6.89480600
H	-5.15408500	1.39500100	-7.70524700
H	-5.41424400	-0.25988500	-7.04989100
O	-3.22815200	2.71378300	-6.71875600
O	3.80668300	1.79475400	-6.78679100
C	-3.02445500	-0.84575100	-6.80375600
C	-1.57828000	-1.02238000	-6.78101200
C	-1.03455900	-2.28714100	-6.52256400
C	0.36558200	-2.50047000	-6.46167400
C	1.23879900	-1.40807800	-6.72233300
C	2.68607600	-1.61148200	-6.75445300
C	-0.71333400	0.10394000	-6.80736100
C	0.71333400	-0.10394000	-6.80736100
C	-2.68607600	1.61148200	-6.75445300
C	-1.23879900	1.40807800	-6.72233300

C	-0.36558200	2.50047000	-6.46167400
C	1.03455900	2.28714100	-6.52256400
C	1.57828000	1.02238000	-6.78101200
C	3.02445500	0.84575100	-6.80375600
C	4.92721900	-0.69999300	-6.89480600
H	5.41424400	0.25988500	-7.04989100
H	5.26174400	-1.14934000	-5.95604500
H	5.15408500	-1.39500100	-7.70524700
H	-5.26174400	1.14934000	-5.95604500
C	0.68219100	-3.77511600	-5.90498300
C	-0.43069600	-4.48408200	-5.51945500
S	-1.92850700	-3.68831000	-5.99792700
H	1.69970900	-4.06944500	-5.68052100
C	-0.68219100	3.77511600	-5.90498300
C	0.43069600	4.48408200	-5.51945500
S	1.92850700	3.68831000	-5.99792700
H	-1.69970900	4.06944500	-5.68052100
O	-2.98828300	6.93659100	-1.97313000
N	-2.79517700	6.96135900	0.29960900
O	4.06821700	6.57308500	-2.55473800
N	4.23463400	6.62443600	-0.30268100
C	-4.24288900	7.06776300	0.46316900
H	-4.47298600	7.92919100	1.09284500
H	-4.68309200	7.17783100	-0.52484000
O	-2.63760200	6.86469700	2.55087700
O	4.42373100	6.56347900	1.96950300
C	-2.26835700	6.90273700	-0.98661500
C	-0.80102600	6.80802500	-1.09695700
C	-0.22236000	6.52741400	-2.32284200
C	1.20233800	6.41198700	-2.47384900
C	2.01424100	6.63719300	-1.35338600
C	3.48357700	6.60167200	-1.48361600
C	0.01090200	6.82500200	0.06971800
C	1.42772500	6.75870400	-0.07040900
C	-2.05100000	6.85249900	1.48026600
C	-0.58505600	6.75133600	1.35111300
C	0.20199900	6.44158300	2.47184500
C	1.63084200	6.42088700	2.31819800
C	2.23411500	6.65324300	1.09387400
C	3.70419200	6.60679300	0.98320000
C	5.68582100	6.59335900	-0.46654200
H	6.13432000	6.63779300	0.52286400
H	5.97776700	5.67426500	-0.97933400
H	5.99941900	7.44245900	-1.07680500
H	-4.62459100	6.17015200	0.95480500
C	1.55582800	5.86106200	-3.74541900
C	0.46209300	5.49437000	-4.48629500
S	-1.06040500	6.05115900	-3.77637900
H	2.57234300	5.59479000	-4.00136100
C	-0.20199900	5.92132900	3.73906500
C	0.85155600	5.43994200	4.47508900
S	2.41785000	5.84500000	3.76213700
H	-1.23898400	5.75993900	3.99680500

VIII. Reference

1. (a) Zhang, L.; Zhang, G.; Qu, H.; Todarwal, Y.; Wang, Y.; Norman, P.; Linares, M.; Surin, M.; Zhang, H.-J.; Lin, J.; Jiang, Y.-B. *Angew. Chem. Int. Ed.* **2021**, *60*, 24543. (b) Y. Pan, J. Lin, *J. Xiamen Univ. (Nat. Sci.)* **2025**, *64*, 775.
2. Frisch, M. J.; Trucks, G. W.; Schlegel, H. B.; Scuseria, G. E.; Robb, M. A.; Cheeseman, J. R.; Scalmani, G.; Barone, V.; Mennucci, B.; Petersson, G. A.; Nakatsuji, H.; Caricato, M.; Li, X.; Hratchian, H. P.; Izmaylov, A. F.; Bloino, J.; Zheng, G.; Sonnenberg, J. L.; Hada, M.; Ehara, M.; Toyota, K.; Fukuda, R.; Hasegawa, J.; Ishida, M.; Nakajima, T.; Honda, Y.; Kitao, O.; Nakai, H.; Vreven, T.; Montgomery, J. A., Jr.; Peralta, J. E.; Ogliaro, F.; Bearpark, M.; Heyd, J. J.; Brothers, E.; Kudin, K. N.; Staroverov, V. N.; Kobayashi, R.; Normand, J.; Raghavachari, K.; Rendell, A.; Burant, J. C.; Iyengar, S. S.; Tomasi, J.; Cossi, M.; Rega, N.; Millam, J. M.; Klene, M.; Knox, J. E.; Cross, J. B.; Bakken, V.; Adamo, C.; Jaramillo, J.; Gomperts, R.; Stratmann, R. E.; Yazyev, O.; Austin, A. J.; Cammi, R.; Pomelli, C.; Ochterski, J. W.; Martin, R. L.; Morokuma, K.; Zakrzewski, V. G.; Voth, G. A.; Salvador, P.; Dannenberg, J. J.; Dapprich, S.; Daniels, A. D.; Farkas, O.; Foresman, J. B.; Ortiz, J. V.; Cioslowski, J.; Fox, D. J., Gaussian 16, Revision A.03; Gaussian, Inc., Wallingford CT, 2016.
3. Zhao Y., Truhlar D. G. The M06 suite of density functionals for main group thermochemistry, thermochemical kinetics, noncovalent interactions, excited states, and transition elements: two new functionals and systematic testing of four M06-class functionals and 12 other functionals. *Theor. Chem. Account.* **2008**, *120*, 215.
4. Grimme, S.; Antony, J.; Ehrlich, S.; Krieg, H. *J. Chem. Phys.* **2010**, *132*, 154104.
5. (a) Ditchfield, R.; Hehre, W. J.; Pople, J. A. *J. Chem. Phys.* **1971**, *54*, 724. (b) Hehre, W. J.; Ditchfield, R.; Pople, J. A. *J. Chem. Phys.* **1972**, *56*, 2257. (c) Hariharan, P. C.; Pople, J. A. *Theor. Chim. Acta* **1973**, *28*, 213.
6. Glendening, E. D.; Badenhoop, J. K.; Reed, A. E.; Carpenter, J. E.; Bohmann, J. A.; Morales, C. M.; Landis, C. R.; Weinhold, F. NBO 7.0; Theoretical Chemistry Institute, University of Wisconsin: Madison, WI, 2013. http://nbo7.chem.wisc.edu/.
7. Chen, Z.; Wannere, C. S.; Corminboeuf, C.; Puchta, R.; Schleyer, P. v. R. *Chem. Rev.* **2005**, *105*, 3842.
8. Yanai, T.; Tew, D. P.; Handy, N. C. *Chem. Phys. Lett.* **2004**, *393*, 51.
9. Geuenich, D.; Hess, K.; Köhler, K.; Herges, R. *Chem. Rev.* **2005**, *105*, 3758.
10. (a) Szczepanik, D. W.; Andrzejak, M.; Dyduch, K.; Zak, E.; Makowski, M.; Mazur, G.; Mrozek, J. *Phys. Chem. Chem. Phys.* **2014**, *16*, 20514. (b) Szczepanik, D. W.; Andrzejak, M.; Dominikowska, J.; Pawelek, B.; Krygowski, T. M.; Szatylowicz, H.; Solà, M. *Phys. Chem. Chem. Phys.* **2017**, *19*, 28970.
11. Legault, C. Y. CYLview, 1.0b ed.; Université de Sherbrooke: Sherbrooke, Québec, Canada, 2009; http://www.cylview.org.
12. Lu, T.; Chen, F. *J. Comput. Chem.* **2012**, *33*, 580.
13. Humphrey, W.; Dalke, A.; Schulten, K. *J. Mol. Graphics* **1996**, *14*, 33.
14. Yamaguchi, K. *Chem. Phys. Lett.* **1975**, *33*, 330.
15. Nakano, M.; Kishi, R.; Nitta, T.; Kubo, T.; Nakasuji, K.; Kamada, K.; Ohta, K.; Champagne, B.; Botek, E.; Yamaguchi, K. *J. Phys. Chem. A* **2005**, *109*, 885.
16. (a) Nakano, M.; Champagne, B. *WIREs Comput. Mol. Sci.* **2016**, *6*,198. (b) Nakano, M. *Top*

Curr. Chem. (Z) **2017**, *375*, 47.
17. Guo, J.; Yang, Y.; Dou, C.; Wang, Y. *J. Am. Chem. Soc.* **2021**, *143*, 18272.
